# Consequences of Pore Polarity and Solvent Structure on Epoxide Ring-Opening in Lewis and Brønsted Acid Zeolites

**DOI:** 10.1021/jacsau.4c00398

**Published:** 2024-07-09

**Authors:** David S. Potts, Jessica K. Komar, Matthew A. Jacobson, Huston Locht, David W. Flaherty

**Affiliations:** † Department of Chemical and Biomolecular Engineering, University of Illinois Urbana-Champaign, Urbana, Illinois 61801, United States; ‡ School of Chemical and Biomolecular Engineering, Georgia Institute of Technology, Atlanta, Georgia 30332, United States

**Keywords:** solvent structure, solid–liquid interfaces, ring opening, calorimetry, structure function relationship, acidic zeolites, regioselectivity, zeolite polarity

## Abstract

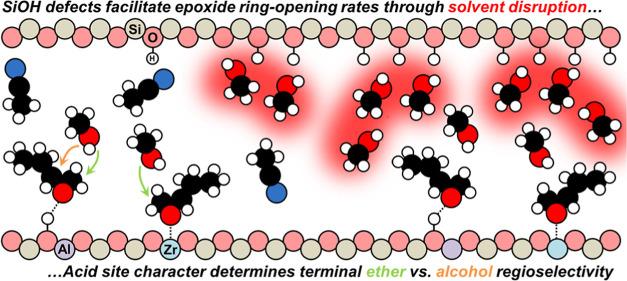

The structure of solvent molecules within zeolite pores influences the rates and selectivities of catalytic reactions by altering the free energies of reactive species. Here, we examine the consequences of these effects on the kinetics and thermodynamics of 1,2-epoxybutane (C_4_H_8_O) ring-opening with methanol (CH_3_OH) in acetonitrile (CH_3_CN) cosolvent over Lewis acidic (Zr-BEA) and Brønsted acidic (Al-BEA) zeolites of varying (SiOH)_*x*_ density. Despite ostensibly identical reaction mechanisms across materials, turnover rates depend differently on (SiOH)_*x*_ density between acid types. (SiOH)_*x*_-rich Zr-BEA (Zr-BEA-OH) provides ∼10 times greater rates than a (SiOH)_*x*_-poor material (Zr-BEA-F), while Al-BEA-OH and Al-BEA-F give turnover rates within a factor of 2. Zr-BEA-OH shows more positive activation enthalpies and entropies than Zr-BEA-F across the range of [CH_3_OH], which reflect the displacement of solvent molecules and lead to greater rates in Zr-BEA-OH due to the dominant role of entropic gains. Measurements of the density and composition of solvent within the pores show that the (SiOH)_*x*_ nests within Zr-BEA-OH promote hydrogen-bonded solvent structures distinct from Zr-BEA-F, while the Brønsted acid sites confer interactions similar to (SiOH)_*x*_ nests and give solvent structures within Al-BEA-F that resemble those within Al-BEA-OH. Correlations between apparent activation enthalpies and C_4_H_8_O adsorption enthalpies show that interactions with solvent molecules give proportional changes to both C_4_H_8_O adsorption and ring-opening transition state formation. The differences in intrapore environment carry consequences for both rates and regioselectivities of epoxide ring-opening, as demonstrated by product regioselectivities that increase by a factor of 3 in response to changes in solvent composition and the type of acid site in the *BEA structure (i.e., Lewis or Brønsted). These results demonstrate the ability to control rates, regioselectivities, and adsorption thermodynamics relevant for industrially relevant liquid-phase reactions through the design of noncovalent interactions among solvating molecules, reactive species, and (SiOH)_*x*_ functions.

## 1 Introduction

Zeolites serve as useful materials for catalysis and adsorption because the subnanometer pores within zeolites can stabilize guest molecules of selected shapes and sizes.^1,2^ The zeolite pore walls stabilize these adsorbate molecules through nonspecific van der Waals interactions.^3,4^ The introduction of a condensed intrapore phase adds further complexity to systems due to specific interactions (e.g., hydrogen bonds) between reactants, spectator molecules (e.g., solvent), and pore functions.^5−8^ These interactions introduce thermodynamic nonidealities that alter the excess free energies (*G*^ε^) of adsorbates and transition states.^9^ Altering the surface functions within zeolite pores leads to differences in the arrangement of solvent and reactant molecules near active sites, which provides an opportunity to influence the rates and selectivities of catalytic and separation processes.

The presence of intrapore silanol ((SiOH)_*x*_) functions strongly affects both adsorption and catalysis in zeolites. Vega-Vila and Gounder demonstrated that hydrophobic Sn-incorporated zeolites stabilize 2–5 times greater densities of methanol molecules than hydrophilic materials, which contain (SiOH)_*x*_ groups that promote extended hydrogen-bonded structures of methanol (CH_3_OH) that pack the pores less densely.^10^ In other reports, hydrophilic zeolites adsorb 5 to 100-fold more water (H_2_O) than hydrophobic zeolites because (SiOH)_*x*_ groups stabilize networks of H_2_O molecules.^11−14^ Pore polarity can also influence the selectivity of adsorption from binary mixtures, as Zhang et al. found that the selectivity for ethanol adsorption from ethanol and H_2_O mixtures within hydrophobic pores exceeds that of hydrophilic pores by 5 times.^15^ The differences in the structure of intrapore liquids between (SiOH)_*x*_-rich and (SiOH)_*x*_-poor materials also affect rates and selectivities of zeolite-catalyzed reactions. For example, hydrophilic Ti- and Sn-BEA zeolites give 5–10 times lower turnover rates than the hydrophobic variants for aqueous-phase glucose isomerization.^10,14,16−18^ Measured activation enthalpies and entropies suggest that the H_2_O networks in hydrophilic pores provide a significant entropic destabilization to the hydrophilic transition state for isomerization, which exceeds the corresponding enthalpic stabilization and leads to lower rates. Conversely, hydrophilic Ti-incorporated zeolites provide greater turnover rates than hydrophobic materials for alkene epoxidations with aqueous hydrogen peroxide (H_2_O_2_) in acetonitrile (CH_3_CN),^11,13,19−22^ CH_3_OH,^11,19,20,23^ and gamma-butyrolactone^11^ (C_4_H_6_O_2_) solvents. Trends of rates and activation barriers indicate that the hydrophobic alkyl tail of the epoxidation transition state disrupts hydrogen bonds between H_2_O molecules within hydrophilic pores, leading to entropic gains that increase turnover rates. The examples discussed here demonstrate that solvent molecules influence catalysis and adsorption in zeolites through changes in the excess free energy of adsorbates (*G*^ε^).

**Scheme 1:**
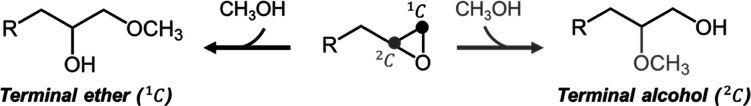
Primary Reaction Products Formed by Epoxide Ring-Opening with a CH_3_OH Nucleophile The nucleophile can attack at the primary (^1^C) or secondary (^2^C) carbon on the epoxide ring.

Beyond epoxidation, manipulating the intrapore solvent environment in zeolites provides opportunities to influence the rates and regioselectivities of the subsequent epoxide ring-opening reaction (Scheme 1). An early study found that ion-exchanged FAU zeolites show greater selectivities but lower turnover rates to secondary alcohols (from ^2^C attack) in polar solvents compared to nonpolar solvents for the reaction between 1,2-epoxyoctane and sodium azide (NaN_3_), although the authors do not provide clear hypotheses for these trends.^24^ Brunelli and co-workers found that the postsynthetic fluoride treatment of Sn-BEA increases the rate of epichlorohydrin ring-opening with CH_3_OH by a factor of 2 compared to an untreated Sn-BEA material.^25^ They attributed the rate increases to a decrease in the density of (SiOH)_*x*_ groups that leads to entropic gains for ring-opening. More recent work used site quantification, rate measurements, and ^15^N NMR of adsorbed pyridine to show that Sn sites adjacent to (SiOH)_*x*_ nests provide greater turnover rates for epoxide ring-opening than Sn sites adjacent to a point SiOH defect.^26^ These findings indicate that the (SiOH)_*x*_ structure near Sn atoms influences ring-opening kinetics, which may stem from differences in local solvent structure. Inspired by these findings, our group examined the mechanism of ring-opening 1,2-epoxybutane (C_4_H_8_O) with CH_3_OH over hydrophilic M-BEA zeolites and demonstrated that addition of an aprotic cosolvent (CH_3_CN) increases the preference to form the terminal ether product (from ^1^C attack).^27^ Measurements of activation barriers provide evidence that differences in regioselectivity arise from differences in the solvent composition near active sites that affect the stability of transition states for each regioisomers to different extents. To summarize, the acid site and (SiOH)_*x*_ density significantly influence epoxide ring-opening and other liquid-phase chemistries. While industrial ring-opening processes commonly utilize Brønsted^28−30^ and Lewis^31−33^ acid catalysts, the literature lacks comparisons of how these different acid types affect the solvent environment during liquid-phase catalysis. The ability of Brønsted acidic protons (H^+^) to detach from the zeolite framework and form ionic solvent clusters intuitively leads to different solvent structures near catalytic active sites than for Lewis acidic metal atoms that remain bound to the framework.^5,13,34^ Still, the effect of the interplay between (SiOH)_*x*_ functions and Lewis and Brønsted acid sites on the intrapore solvent environment in zeolites remains unclear. The strong influence of the choice of solvent and zeolite hydrophilicity shown in previous ring-opening studies underscores the importance of establishing these connections.

Here, we reveal the impact of varying the (SiOH)_*x*_ density of Lewis acidic Zr- and Brønsted acidic Al-BEA zeolites on the intrapore solvent environment and, consequently, rates and regioselectivities of C_4_H_8_O ring-opening with CH_3_OH in CH_3_CN cosolvent. Despite apparently identical reaction mechanisms, turnover rates over (SiOH)_*x*_-rich Zr-BEA (Zr-BEA-OH) exceed those over a (SiOH)_*x*_-poor material (Zr-BEA-F) by ∼10 times. In contrast, turnover rates over Al-BEA-OH and Al-BEA-F materials differ by less than two times. Measurements of liquid and vapor solvent uptake provide evidence that Zr-BEA-OH adsorbs greater quantities of CH_3_OH and promotes greater intrapore ratios of CH_3_OH to CH_3_CN than Zr-BEA-F, while Al-BEA-OH and Al-BEA-F show more similar solvent uptakes. The Al-BEA materials show nearly identical activation enthalpies and entropies for ring-opening and adsorption enthalpies for C_4_H_8_O, which likely lead to similar rates between the catalysts. In contrast, Zr-BEA-OH shows more positive activation enthalpy, entropy, and C_4_H_8_O adsorption enthalpy values than Zr-BEA-F. This trend provides strong evidence that the entropic gain from the disruption of hydrogen-bonded solvent molecules in the Zr-BEA-OH pores leads to greater ring-opening rates than Zr-BEA-F. Reaction regioselectivities depend strongly on the active metal choice and solvent composition but weakly on (SiOH)_*x*_ density, demonstrating that pore polarity influences rates and regioselectivities differently. Cumulatively, these findings show that the intrapore solvent structure depends intimately on the (SiOH)_*x*_ density and active site structure within the *BEA pores, which provides opportunities to control the kinetics and thermodynamics of catalytic reactions (i.e., epoxide ring-opening).

## 2 Methods and Materials

### 2.1 Catalyst Synthesis

M-BEA-OH (M = Al, Zr) were prepared by postsynthetic modification of a commercial Al-BEA material (TOSOH, lot no. 94HA6X02Y; Si/Al = 20). For Zr-BEA-OH, the commercial Al-BEA was treated in refluxing HNO_3_ (Macron Chemicals, 68–70 wt %, 20 cm^3^ g^–1^) at 433 K for ∼24 h to remove Al atoms from the *BEA framework by forming aqueous (AlNO_3_)_3_ complexes. The HNO_3_ treatment was repeated three times to give a thoroughly dealuminated material denoted Si-BEA-OH (Si:Al > 1200 from EDXRF, >3500 from ICP-OES, vide infra). Between acid treatments, the catalyst sample was washed with deionized H_2_O (18.2 MΩ cm, Elga Purelab Flex 2, 50 cm^3^ g_zeolite_^–1^) and recovered with vacuum filtration. For Al-BEA-OH, the parent Al-BEA material was treated in dilute HNO_3_ (1 M) for ∼3 h to partially remove Al atoms from the framework. After the acid treatments, the dealuminated *BEA materials were loaded into quartz boats and placed in a three-zone furnace (Applied Test Systems, 3210), which was heated to 823 K (5 K min^–1^) in flowing air (200 cm^3^ min^–1^; Airgas, Ultra Zero grade) and held at 823 K for 6 h to remove organic residues. The heat treatment resulted in white powder Al-BEA-OH and Si-BEA-OH samples.

Zr-BEA-OH was synthesized by the liquid-phase incorporation of Zr atoms into the Si-BEA-OH material, based on previously reported procedures.^13,35^ Si-BEA-OH was first heated in a round-bottom flask under vacuum (<5 Pa, 473 K) for 2 h to create a moisture-free environment. A rubber seal was then removed from the side arm of the flask, and isopropanol solvent (C_2_H_7_OH, Fisher Chemicals, 20 cm^3^ g_zeolite_^–1^) was poured into the flask under a high flow of Ar (500 cm^3^ min^–1^) to minimize the adsorption of moisture from the air. Zr atoms were incorporated by then adding solid ZrCl_4_ powder (Sigma-Aldrich, ≥99.9% trace metals basis) to the Si-BEA-OH and solvent mixture under flowing Ar (500 cm^3^ min^–1^). The rubber seal was placed back onto the flask side arm, and the mixture was then heated and left overnight under an inert atmosphere (373 K, refluxing solvent, ∼16 h, 50 cm^3^ min^–1^ Ar, Airgas, Ultra High Purity). The resulting material was recovered by vacuum filtration and was heated to 823 K (5 K min^–1^) in flowing air (200 cm^3^ min^–1^) and held at 823 K for 6 h, resulting in a white powder Zr-BEA-OH sample.

M-BEA-F (M = Al, Zr) were synthesized by adapting previously published procedures for hydrothermal synthesis of Ti-BEA-F materials.^16,36^ For each M-BEA-F, 20 g of tetraethylammonium fluoride hydrate (TEAF, Sigma-Aldrich, 98%) was dissolved in 32.1 cm^3^ of deionized H_2_O (18.2 MΩ cm, Elga Purelab Flex 2) in a polypropylene container. Next, 47.4 cm^3^ of tetraethylorthosilicate (TEOS, Sigma-Aldrich, >98 wt %) was added, and the mixture was stirred for 30 min at room temperature. Either 0.455 g of aluminum(III) isopropoxide (Al[OCH(CH_3_)_2_]_3_, AIPO, Sigma-Aldrich, ≥98%) or 0.921 cm^3^ of zirconium(IV) *n*-propoxide in *n*-propanol (Zr[OCH(CH_3_)_2_]_4_, ZrPO, Alfa Aesar, 70 wt % in *n*-propanol) was added, and the solutions were covered and stirred for an additional 16 h to produce white, opaque homogeneous solutions. The covers were removed, and the mixtures were stirred for 12–24 h to evaporate the alcohols formed from the hydrolysis of AIPO or ZrPO (isopropanol or n-propanol) and TEOS (ethanol). The mass of alcohols formed was estimated by assuming complete and stoichiometric hydrolysis of all AIPO, ZrPO, and TEOS added. The solutions were left uncovered to allow the alcohols to evaporate until the mass of the solutions decreased by an amount equal to ∼115% of the calculated mass of the alcohols to increase the likelihood of complete evaporation of these alcohols. The alcohols are assumed to evaporate preferentially before water based on their greater volatility. A mass of deionized H_2_O equivalent to the difference between the total mass evaporated (42–47 g) and the calculated mass of alcohols formed (40–41 g) was added to account for the H_2_O lost during evaporation. The evaporation procedure yielded gels with an approximate molar composition of 1 Si:0.006 Al or Zr:0.56 TEAF:8.89 H_2_O. The gels were subsequently transferred to Teflon liners (Parr Instruments, 125 cm^3^, Model 4748). A small amount of Si-BEA-OH seeds (3% by mass relative to SiO_2_ in the synthesis gel) from a previous synthesis was added to each gel to promote crystallization. The Teflon liners were loaded into a stainless-steel autoclave (Parr Instruments, 125 cm^3^, Model 4748) and heated to 413 K at dynamic conditions (60 rpm) in a convection oven (Yamato, DKN602C) for 22 days. The resulting materials were recovered by centrifugation, washed with H_2_O, and dried in an oven for 12 h at 343 K. The dried solids were heated at 5 K min^–1^ to 823 K in flowing air (200 cm^3^ min^–1^) and held at 823 K for 6 h to produce white powder Al-BEA-F and Zr-BEA-F samples.

### 2.2 Catalyst Characterization

The crystallinities of the M-BEA materials were confirmed with an X-ray diffractometer (Bruker, D8 Advance) with Cu Kα radiation under ambient conditions. The M-BEA powders were loaded into a polypropylene sample holder. Metal oxide samples were purchased commercially and used as received: γ-Al_2_O_3_ (US Research Nanomaterials, Inc., 20 nm, 99%) and ZrO_2_ (Sigma-Aldrich, 5 μm 99% trace metals basis). The diffractograms of M-BEA-OH (Figure S1a) and M-BEA-F (Figure S1b) match previously reported diffraction patterns for *BEA,^37^ supporting that all M-BEA possess the *BEA framework. Furthermore, the patterns for M-BEA do not possess strong features in the characteristic peak locations for the respective metal oxides (Figure S1c), supporting that negligible quantities of each incorporated metal exist in metal oxide form.

The metal contents of the M-BEA samples were calculated with inductively coupled plasma optical emission spectroscopy (ICP-OES, PerkinElmer, Optima 8300) measurements carried out by staff scientists at the Microanalysis Laboratory at the University of Illinois. The measurements confirm that Zr-BEA-OH and Zr-BEA-F contain negligible quantities of Al (Si:Al ∼4000). Each Zr-BEA possesses similar Si:Zr values (131 for Zr-BEA-OH, 103 for Zr-BEA-F), and each Al-BEA shows a similar Si:Al ratio (69 for Al-BEA-OH, 61 for Al-BEA-F). Reported turnover rates are calculated by normalizing rates with the metal loadings determined by ICP-OES. Metal loadings and Si:Al ratios for each zeolite are shown in Table 1. The M-BEA-F materials were also tested for residual fluorine content using the ion-selective electrode (ISE) method (Orion, Thermo Scientific). No fluorine is detected in these materials from the ISE method, which has a detection limit of 0.01 ppm. This indicates that the M-BEA-F catalysts contain no (or sub ppm) levels of fluorine after hydrothermal synthesis, which does not impact the catalytic and thermodynamic measurements presented below.

**Table 1:** Characterization of the Chemical, Physical, and Electronic Properties of Synthesized M-BEA

catalyst	M wt %^a^	Si:M^a^	Si:Al^a^	band gap (eV)^b^	Φ_IR_^c^	BET surface area (m^2^ g^–1^)^d^	micropore volume (cm^3^ g^–1^)^e^	active metal (%)^f^
Al-BEA-OH	0.63	69	69		1.44	602 ± 11	0.145	97 ± 4
Al-BEA-F	0.72	61	61		0.19	516 ± 13	0.172	92 ± 3
Zr-BEA-OH	1.03	131	3980	5.8	1.41	536 ± 12	0.142	104 ± 7
Zr-BEA-F	1.44	103	4090	5.7	0.21	522 ± 13	0.170	96 ± 2

a Measured with ICP-OES.

b Extracted from leading edge of Tauc plot from DRUV–vis.

c Calculated from infrared transmission spectra of dehydrated M-BEA samples.

d Determined from Ar physisorption.

e Calculated using the *t*-plots from Ar physisorption.

f Measured from in situ 1,2-diphenylethylenediamine site titrations.

The dispersity of the M atoms in the M-BEA catalysts was probed with diffuse reflectance UV–visible (DRUV–vis) spectroscopy. M-BEA were mixed and finely ground with magnesium oxide (MgO, Sigma-Aldrich, 99.9995%) at a MgO:M-BEA mass ratio of 10:1. MgO was used as a background for measurements. The total reflectance sample spectra were obtained with a UV–vis spectrophotometer (Varian, Cary 5G) under ambient conditions. γ-Al_2_O_3_ and ZrO_2_ were again used as received. The band gap energies (Table 1) were determined by extrapolating the linear portion of the Tauc plots [*F*(*R*)·*hv*]^1/2^) to the horizontal axis to determine the minimum energy of photons adsorbed (eV) (Figure S2). The band gap of ZrO_2_ was measured in a similar manner as the M-BEA materials (Figure S2). Each Zr-BEA shows a greater band gap than ZrO_2_, supporting that the Zr atoms are substituted within the framework at dispersed locations. Both Al-BEA materials and Al_2_O_3_ show very weak or no reflectance features, likely because the band gap of each material exceeds the scan range of the spectrophotometer. Nevertheless, the low weight loadings of each Al-BEA (Si:Al ∼60–70) and the unfavorable nature of Al–O–Al pairs (Loewenstein’s rule^38^) support that Al atoms are well dispersed within the *BEA framework.

The coordination of metal atoms within M-BEA was examined using Raman spectroscopy. Ex situ Raman spectra were measured at ambient conditions on pressed catalyst pellets with a Raman spectrometer (Renishaw, InVia) equipped with a 532 nm laser. The accumulation time was 20 s per scan, and each Raman spectra was calculated by averaging 10 scans. The power density at the sample was approximately 2 mW μm^–2^ (Gentec-EO, PRONTO-SI). The Raman spectra for each M-BEA-OH (Figure S3) and M-BEA-F (Figure S4) lack discernible features in the regions where the respective metal oxides show strong features, indicating the absence of M-O-M bonds. Together with the high band gap energies (Table 1), these spectra support that the M atoms in each material predominantly reside in tetrahedral positions within *BEA.

The relative density of (SiOH)_*x*_ groups within M-BEA was measured with infrared spectroscopy. A background spectrum was first taken with an empty transmission cell configured with CaF_2_ windows. The cell was loaded into a Fourier transform infrared spectrometer (Bruker, Vertex 70) equipped with a liquid N_2_-cooled HgCdTe detector, as described previously.^39^ The cell was connected to a temperature controller and gas manifold, then heated to 573 K (∼5 K min^–1^) and held for 2 h in flowing Ar (50 cm^3^ min^–1^, 99.997%, Airgas) to desorb volatile compounds and H_2_O. The background spectrum was then collected at 573 K (128 scans, 4 cm^–1^). Each M-BEA was pressed into a pellet and loaded into the transmission cell. The cell was loaded into the spectrometer, and the spectra for M-BEA (Figure S5) were obtained in the same manner as the background measurement. The vibrational features of the infrared spectra at 1800–2100 and 3300–3750 cm^–1^ represent the Si–O–Si overtones^40^ of the *BEA framework and *v*(O–H) of (SiOH)_*x*_ groups,^41,42^ respectively. The broad features from 3300 to 3700 cm^–1^ arise from SiOH defects containing multiple hydroxyl moieties within (SiOH)_*x*_ groups. The sharper feature at ∼3750 cm^–1^ represents isolated SiOH defects that do not interact with other SiOH.^41,42^ The ratio of the areas for *v*(O–H) and *v*(Si–O–Si) at 1865 and 2000 cm^–1^ gives a relative measure of SiOH density defined as Φ_IR_ (eq 1) in each M-BEA. The isolated SiOH feature is excluded from *A*_ν(O–H)_ in eq 1. The *v*(O–H) region was deconvoluted into multiple peaks (see Figure S6), and the isolated SiOH peak area was subtracted from the combined area of the *v*(O–H) region.
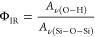
1

Values of Φ_IR_ (Table 1) indicate that the M-BEA-OH materials contain greater densities of (SiOH)_*x*_ groups than M-BEA-F. Calculation estimates based on the quantity of Al removed and M incorporated indicate that the M-BEA-OH materials contain 2.1–2.6 (SiOH)_*x*_ (unit cell)^−1^ (see Section S1.5). M-BEA-F were synthesized hydrothermally without Al present, so we cannot make the same calculation of (SiOH)_*x*_ (unit cell)^−1^. However, previously established trends^43^ between Φ_IR_ and (SiOH)_*x*_ suggest that the M-BEA-F samples synthesized for this work (Φ_IR_ ∼0.20, Table 1) contain fewer than 0.1 (SiOH)_*x*_ per unit cell.

The presence of Lewis and Brønsted acid sites in M-BEA was characterized by infrared spectra of adsorbed pyridine (C_5_H_5_N, Sigma-Aldrich, >99%) and deuterated acetonitrile (CD_3_CN, Cambridge Isotope Laboratories, 99.8% D atom). The catalysts were pelletized, loaded into the spectrometer, and pretreated at 573 K, as described above. The cell was then cooled to either 393 K (C_5_H_5_N) or 303 K (CD_3_CN). A background spectrum was taken before flowing the adsorbates. C_5_H_5_N or CD_3_CN were then introduced with a syringe pump (KD Scientific, Legato) and vaporized into a stream of flowing Ar (20 cm^3^ min^–1^) within the heated gas-transfer lines. Reported spectra were collected after the absorbance features had reached a steady state. Section S1.6 presents the spectra and a detailed analysis of the results.

The structure of Al atoms within the Al-BEA materials was also examined with ex situ ^27^Al NMR spectroscopy (Bruker AVIII 400, 10 kHz spin speed). The Al-BEA samples were loaded into a zirconia-based rotor (Bruker, 4 mm, kel-f cap). The rotor was then loaded into the NMR spectrometer, where spectra were collected at ambient temperature. The spectra were referenced to aqueous Al^3+^ at 0.24 ppm. The reported spectra are an average of 1024 scans, with a 0.6 μs pulse time and 1 s relaxation time. Figures S9 and S10 report the ^27^Al NMR spectra for the bare Al-BEA samples and Al-BEA impregnated with C_5_H_5_N. These spectra provide insight into the structure of Al atoms with and without adventitious H_2_O present.

The morphology of the M-BEA materials was examined with scanning electron microscopy (SEM). The samples were dispersed on double-sided carbon tape attached to an SEM holder. A sputter coater (Emitech, K575) was then used to coat the materials with an Au–Pd alloy. Au–Pd provides a conductive surface layer that inhibits surface charging and improves the SEM signal quality. The sample holder was loaded into the microscope (Hitachi-S 4800) and degassed before taking images. The micrographs were obtained at a working distance of 10 mm with an accelerating voltage of 5 kV. The SEM measurements reveal that M-BEA-F materials show larger particle sizes than M-BEA-OH. The SEM images for all M-BEA materials are displayed in Figure S11.

The surface area and micropore volumes of each M-BEA were determined using gas-phase adsorption isotherms of Ar (87 K) on a volumetric adsorption instrument (Micromeritics, 3Flex). The M-BEA samples were pelletized and sieved to retain particles from 100–250 μm in diameter. The samples were degassed under dynamic vacuum prior to adsorption (<7 × 10^–4^ Pa, 673 K, 3 h). The surface area shown in Table 1 was calculated using the Rouquerol-modified Brunauer–Emmett–Teller (BET) method,^44^ while the micropore volume was determined from the *t*-plot method.^45^Figure S12 shows the Ar isotherms, and Table S4 presents the total and external surface area obtained from the Ar isotherms for each M-BEA. The surface area and pore volumes differ by less than 1.25 times between M-BEA-OH and M-BEA-F in each case but external surface area, in which M-BEA-OH materials show ∼2 times greater area than M-BEA-F. The similarities in these values suggest that differences in accessible pore volume and surface area do not contribute significantly to changes in epoxide ring-opening rates discussed in Section 3 (vide infra).

Collectively, the characterization of the M-BEA materials gives strong evidence that each material possesses a crystalline *BEA framework containing similar densities of (SiOH)_*x*_. All M-BEA lack spectroscopically detectable quantities of the respective metal oxides and plausibly contain atomically dispersed metal atoms incorporated at framework positions.

### 2.3 Epoxide Ring-Opening Turnover Rate Measurements

Turnover rates for 1,2-epoxybutane (C_4_H_8_O, TCI Chemicals, >99.0%) ring-opening with methanol (CH_3_OH, Sigma-Aldrich, ≥99.9%) in acetonitrile (CH_3_CN, Fisher Chemical, HPLC grade) solvent were measured in three-necked round-bottom flasks with magnetic stirring (700 rpm). Benzene (Sigma-Aldrich, thiophene-free, >99%) was included in the reaction mixture as an internal standard. The flasks were submerged in a temperature-controlled bath containing either H_2_O (298–318 K) or silicone oil (Sigma-Aldrich, viscosity 100 cSt) (323–328 K) on a hot plate (Corning, PC-420D). The flasks were also connected to reflux condensers to prevent evaporative losses. The reaction mixture was stirred for 0.5 h at the reaction temperature before taking an initial aliquot (∼0.5 cm^3^) to determine the initial concentration of C_4_H_8_O and the ring-opening products, 1-methoxy-2-butanol (C_5_H_12_O_2_, 1M2B) and 2-methoxy-2-butanol (C_5_H_12_O_2_, 2M1B). M-BEA (∼15–60 mg) was then added to initiate the reaction, and aliquots were taken as a function of time with a syringe equipped with a polypropylene filter (Tisch Scientific, 0.22 μm) to separate the sample solution from the catalyst. The concentrations of C_4_H_8_O, benzene, and ring-opening products were quantified as a function of time with a gas chromatograph (GC) (Agilent, 6850) equipped with a liquid autosampler, flame ionization detector, and polysiloxane column (HP-1, Agilent, 19091Z-115E). Elution times and sensitivity factors were determined using commercially obtained samples of C_4_H_8_O, CH_3_OH, benzene, 1M2B (TCI Chemicals, >93.0%), and 2M1B (TCI Chemicals, >98.0%) (Section S2).

Reagents and solvents were used as received (i.e., not dried) prior to their use in rate measurements. Previous works demonstrate that H_2_O may reversibly bind to Lewis^12,16^ or Brønsted^34,46^ acid sites in zeolites and influence reaction turnover rates for chemistries including alkene epoxidation.^11,13^ Here, we exclude significant contributions from H_2_O on ring-opening rates and thermodynamics for two reasons. First, ^1^H NMR of the CH_3_OH and CH_3_CN do not contain detectable features corresponding to H_2_O, which indicates these solvents contain less than 5 mM H_2_O based on measured detection limits.^11^ Second, we do not observe detectable quantities of 1,2-butanediol during reactions, which demonstrates that C_4_H_8_O does not undergo nucleophilic attack by H_2_O.

Turnover rates were measured in CH_3_CN cosolvent to allow for control of both C_4_H_8_O and CH_3_OH concentrations ([C_4_H_8_O], [CH_3_OH], where [*x*] denotes the concentration of species *x*). Measurements were also made in neat CH_3_OH (24.7 M CH_3_OH). The reaction yields two possible products, 1-methoxy-2-butanol (1M2B, a terminal ether) or 2-methoxy-1-butanol (2M1B, a terminal alcohol) (see Scheme 1 above). Initial turnover rate values were calculated by fitting turnover numbers (moles of product per moles of active metal) as a function of time to a second-order polynomial with the turnover rate fixed at zero at time equal to zero, then determining the derivative at time equal to zero. All reported turnover rates were measured at differential conversion (<10%) with an uncertainty of ∼10%, as demonstrated with replicated measurements. The carbon balance closes within 90–110% for all reported measurements. Furthermore, the carbon selectivity to the desired 1M2B and 2M1B products exceeds 90% across all measurements. Two minor peaks appear at similar GC retention times to the ring-opening products. While these products have not been identified, we excluded many likely species that may form by side reactions of C_4_H_8_O, including butanone, 1-butanol, 2-butanol, butyraldehyde, crotonaldehyde, crotyl alcohol, 1,2-butanediol, and dibutyl ether (Section S2). In addition, the unknown peaks evolve in the absence of CH_3_OH, which demonstrates these do not form by secondary reaction of either ring-opening product with CH_3_OH or C_4_H_8_O. Consequently, we conclude these peaks signify the reactions between C_4_H_8_O and other species present, such as atmospheric CO_2_ to form 1,2-butylene carbonate (C_5_H_8_O_3_) or CH_3_CN^47,48^ to form C_6_H_10_ON.

Hot filtration experiments were conducted to determine if the metal atoms or catalytic H^+^ sites leach from the *BEA framework during reactions and form homogeneous complexes active for ring-opening. A large aliquot (∼4 cm^3^) was taken ∼600–900 s after adding the catalyst, filtered with a polypropylene filter (Tisch Scientific, 0.05 μm) to separate the catalyst, then transferred into a scintillation vial (20 cm^3^, Trident Technology). The vial was transferred to a hot plate, which was preheated to 308 K and stirred at 700 rpm. Aliquots (∼0.5 cm^3^) were taken from the vial as a function of time. Figure S16 in Section S3 shows that the product concentrations change negligibly after filtering out the solid catalyst but continue to increase in the presence of the catalyst. Furthermore, a dealuminated *BEA material (Si-BEA-OH) shows product formation rates per gram of catalyst at least 13 times less than all M-BEA materials (Table S7), demonstrating that (SiOH)_*x*_ and Si–O–Si functions do not contribute significantly to the measured ring-opening turnover rates over M-BEA. These experiments provide strong evidence that the ring-opening of C_4_H_8_O with CH_3_OH proceeds solely at active sites within the *BEA framework.

Mass transfer constraints prevent the measurement of intrinsic kinetic behavior, thus corrupting comparisons between catalysts and convoluting the interpretation of apparent rate measurements.^49^ Therefore, we conducted experimental measurements to detect these potential artifacts and select optimal reaction conditions using methods recently described.^50^ The conditions used for rate measurements here avoid internal and external mass transfer constraints because turnover rates do not depend on the loading of metal atoms for all materials within the range of loadings studied (Figure S17 in Section S3). These measurements demonstrate that all M-BEA materials satisfy the Madon–Boudart criterion^49^ and are free of mass transfer artifacts. In addition, turnover rates exhibit combinations of first- and zero-order dependencies on the concentrations of C_4_H_8_O and CH_3_OH at limiting conditions (Section 3.1).^51^ A mass-transfer limited material would show a sublinear dependence on reactant concentrations because the mean concentration of the reactants throughout the pores would not depend linearly on the fluid phase reactant concentrations.

The percentage of active metal atoms within each M-BEA was probed using in situ site titration experiments with (1*R*,2*R*)-(+)-1,2-diphenylethylenediamine (DPED, Sigma-Aldrich, 97%). Briefly, DPED was added to a mixture of CH_3_CN, benzene, CH_3_OH, and M-BEA at a ratio of DPED to metal atoms ranging from 0 to 1.5. The reaction flask was stirred for 0.5 h at 308 K to allow DPED to bind to the active sites before reaction. The reaction was then initiated by adding C_4_H_8_O, after which an aliquot was quickly taken at ∼0.004 h. Further aliquots were then taken as a function of time. The number of active sites in the materials was quantified by fitting a line to the linear portion of the titration curves shown in Section S4. All materials contain more than 90% active metal atoms, as shown in Table 1.

The DPED titration measurements for Zr-BEA-F suggest that a minor fraction (<5%) of Zr sites may be inactive for C_4_H_8_O ring-opening. While no extraframework Zr features (i.e., ZrO_2_) are observed by Raman or DRUV–vis, we cannot exclude the possibility that a small percentage of ZrO_2_ may exist in Zr-BEA-F and do not catalyze C_4_H_8_O ring-opening. The intensities of the ZrO_2_ features may simply fall below the detection limit of the Raman and DRUV–vis spectrometers. Alternatively, Zr-BEA-F may contain a small number of tetrahedrally incorporated Zr atoms located in close proximity such that the bulky DPED titrant cannot titrate the sites individually,^52,53^ which would cause the DPED titration method to slightly underestimate active metal content. In contrast, the minor fraction of inactive Al sites observed in the titrations for Al-BEA-OH and Al-BEA-F aligns with octahedral Al features present in the ^27^Al NMR spectra (Figures S9 and S10). Based on the peak area ratios of tetrahedral to octahedral Al in the presence of C_5_H_5_N, the ^27^Al NMR spectra predict 4% and 10% of Al atoms exist as octahedral species in Al-BEA-OH and Al-BEA-F, respectively. Adding C_5_H_5_N removes residual H_2_O that forms hydrated octahedral Al species, which provides a more accurate report of the structure of Al atoms than ^27^Al NMR spectra of the bare zeolites (see Section S1.7). The greater intensity of the octahedral NMR feature and the presence of a Lewis acid Al feature in the CD_3_CN FTIR spectra for Al-BEA-F also both agree with the lower active metal percentage in Al-BEA-F (92%) than Al-BEA–OH (97%). These three techniques provide strong evidence that each Al-BEA material contains a minor fraction of extraframework, octahedral, Lewis acidic Al species that do not catalyze C_4_H_8_O ring-opening. We do not believe the presence of these extraframework species convolutes the kinetics in this manuscript because they comprise <10% of metal sites in all materials. Reported rates are normalized by the number of active metal atoms calculated from the DPED titrations.

### 2.4 Liquid-Phase and Gas-Phase Adsorption Measurements

The mole fraction of CH_3_OH in the pores of M-BEA was estimated via liquid-phase uptake measurements from CH_3_CN. The intrapore solvent compositions, shown in Figure 4, were estimated from the amount of solution absorbed by M-BEA and the difference in the liquid phase composition before and after mixing the solution with the catalyst.

The effect of (SiOH)_*x*_ density on gaseous CH_3_OH (293 K) and CH_3_CN (296 K) uptake was determined from gas-phase adsorption isotherms (Micromeritics, 3Flex). The M-BEA samples were pelletized and sieved to retain particles from 100–250 μm in diameter. The CH_3_OH and CH_3_CN used here were HPLC grade (Sigma-Aldrich) and were degassed under three freeze–pump–thaw cycles to ensure that the vapors were contamination free. The samples were degassed under dynamic vacuum prior to adsorption (<7 × 10^–4^ Pa, 673 K, 3 h). The isotherms are presented in Figure 5.

### 2.5 Liquid-Phase Adsorption Enthalpies for Reactants

The heat released upon adsorption of C_4_H_8_O and CH_3_OH was measured with an isothermal titration calorimeter (ITC) (TA Instruments, NanoITC) equipped with sample and reference cells. A brief cleaning procedure was performed to prepare the sample and reference cells for each experiment. First, 500 cm^3^ of a cleaning solution consisting of 2 volume % detergent in DI H_2_O (18.2 MΩ cm, Elga Purelab Flex 2) was flowed through the cell at room temperature. The sample cell was then rinsed with 1000 cm^3^ of DI H_2_O. The sample cell and reference cell were then filled with 350 μL of H_2_O (maximum cell volumes are 500 μL). A 50 μL ITC syringe was filled with H_2_O, then loaded into the instrument. A subsequent electrical calibration used sequential pulses to determine the heat released from a calibrated Pt resistor. Finally, pure DI H_2_O was injected into DI H_2_O to ensure the sample cell was clean enough to begin experiments. The cell was assumed to be clean when the H_2_O–H_2_O injection released negligible amounts of heat (±3 μJ for each injection of 1 μL). The cleaning procedure was repeated if the H_2_O–H_2_O injection did not give sufficiently low heat rates.

In a typical experiment, a slurry of M-BEA (20–40 mg) in CH_3_CN or CH_3_OH was titrated by C_4_H_8_O (0.005 M) or CH_3_OH (0.1 M) diluted in the same solvent used for the slurry. The mass of M-BEA in the sample cell was calculated by taking the difference between the mass added to the slurry and the mass remaining upon evaporation of the residual slurry after loading the cell. The titrations were carried out at 308 K with a stirring rate of 250 rpm. The sample cell was filled with 350 μL of M-BEA and solvent, while the reference cell was filled with 350 μL of solvent. The enthalpies of adsorption were calculated by averaging the integrated heats released upon adsorption of the titrant molecules to M-BEA sites from a 1 μL injection of titrant at low coverages (<0.25 mol titrant (mol M)^−1^). The heat released in this regime remains approximately constant, suggesting that the calculated enthalpies represent the isosteric adsorption enthalpies for each titrant.^43^

## 3 Results and Discussion

### 3.1 Brønsted and Lewis Acid Zeolites Show Distinct Ring-Opening Rate Dependences on Reactant Concentrations and (SiOH)_*x*_ Density

**Figure 1:**
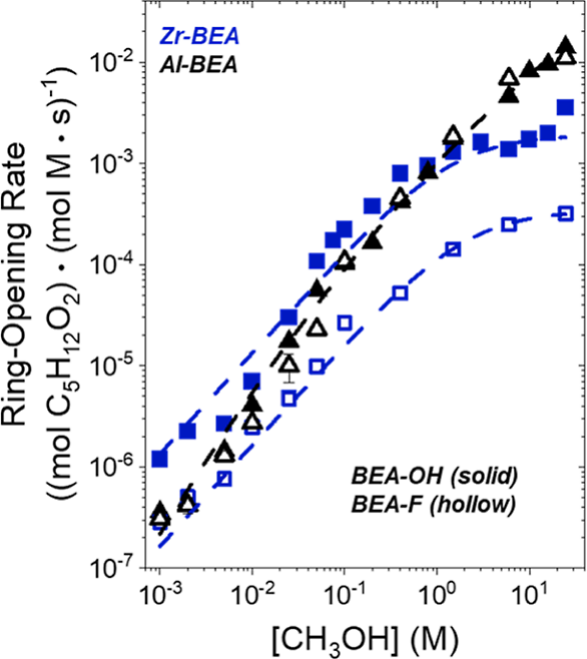
Turnover rates for C_4_H_8_O ring-opening with CH_3_OH as functions of CH_3_OH concentration over hydrophilic (solid points) and hydrophobic (hollow) Zr- (blue) and Al-BEA (black) materials (0.005 M C_4_H_8_O, CH_3_CN solvent, 308 K).

**Figure 2:**
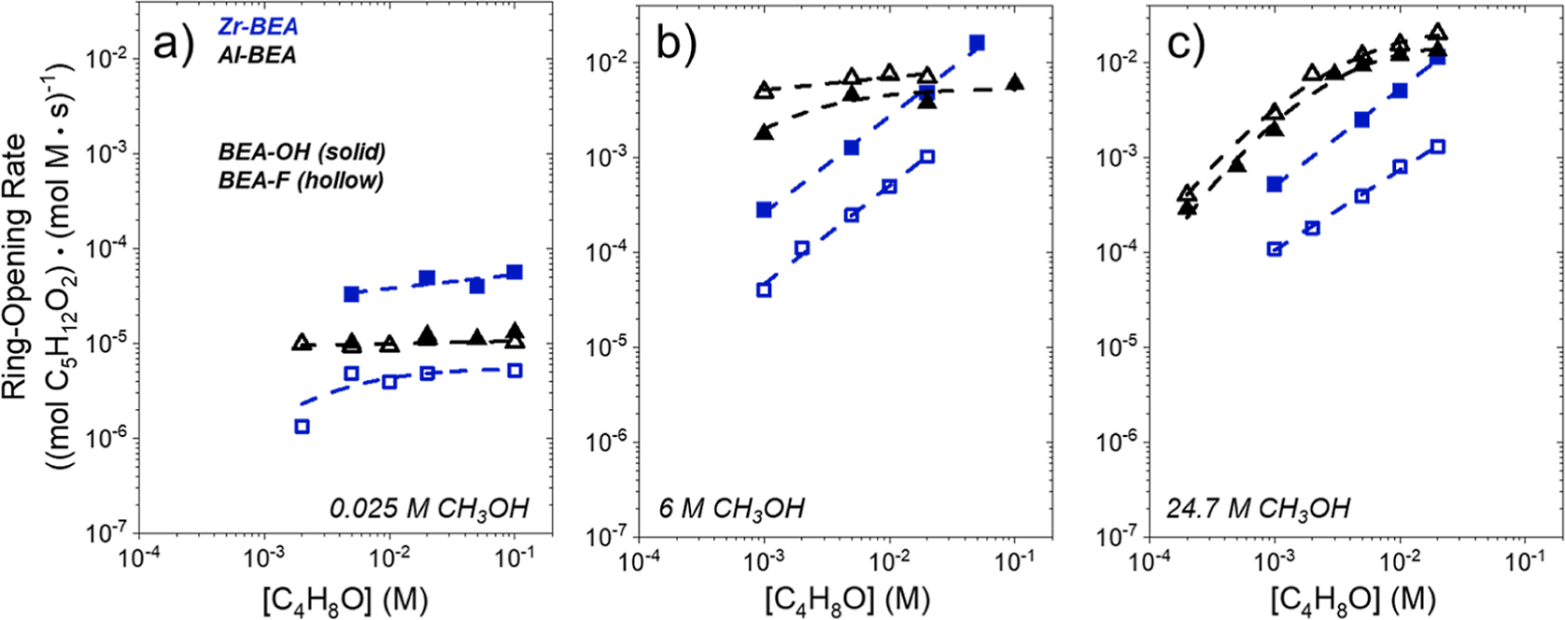
Turnover rates for C_4_H_8_O ring-opening with CH_3_OH as functions of C_4_H_8_O concentration at (a) 0.025 M CH_3_OH, (b) 6 M CH_3_OH, or c) neat (24.7 M) CH_3_OH (CH_3_CN solvent, 308 K) over hydrophilic (solid points) and hydrophobic (hollow) Zr- (blue) and Al-BEA (black) materials.

Turnover rates for C_4_H_8_O ring-opening depend on the concentrations of the reactants and products present, which determine the coverages of species on the active sites within M-BEA. Figures 1 and 2 show that rates depend differently on [CH_3_OH], [C_4_H_8_O], and (SiOH)_*x*_ density between Al-BEA and Zr-BEA materials. The (SiOH)_*x*_-dense Brønsted acid (Al-BEA-OH) shows nearly identical turnover rates to the (SiOH)_*x*_-poor analog (Al-BEA-F) at all conditions. In contrast, ring-opening turnover rates over Zr-BEA-OH exceed rates over Zr-BEA-F by approximately an order of magnitude across the range of [CH_3_OH] and [C_4_H_8_O] examined. Despite Zr-BEA and Al-BEA providing different rate dependences on (SiOH)_*x*_ density, turnover rates over each acid type exhibit nearly identical dependences on reactant concentrations among (SiOH)_*x*_-rich and (SiOH)_*x*_-poor materials. All M-BEA show linear rate dependences on [CH_3_OH] (Figure 1) and a nearly zero-order dependence on [C_4_H_8_O] (Figure 2) at molar ratios of [CH_3_OH] to [C_4_H_8_O] less than 50. These dependencies hold over Al-BEA catalysts up to ratios of [CH_3_OH] to [C_4_H_8_O] around 1200. Turnover rates approach a near zero-order dependence on [CH_3_OH] and linear dependence on [C_4_H_8_O] when ratios of [CH_3_OH] to [C_4_H_8_O] exceed 5000 over Al-BEA materials. However, rates transition to the regime of strong dependence on [C_4_H_8_O] and weak dependence on [CH_3_OH] over the Zr-BEA materials at much lower ratios of [CH_3_OH] to [C_4_H_8_O] (>500). To summarize, the measurements in Figures 1 and 2 demonstrate that the (SiOH)_*x*_ density influences epoxide ring-opening rates over Lewis acid zeolites (Zr-BEA) but do not significantly affect rates over Brønsted acid zeolites (Al-BEA). The (SiOH)_*x*_ density does not influence the dependence of rates on reactant concentrations over either class of materials, with either C_4_H_8_O- or CH_3_OH-derived species saturating the active sites within (SiOH)_*x*_-rich and (SiOH)_*x*_-poor M-BEA materials during C_4_H_8_O ring-opening.

**Scheme 2:**
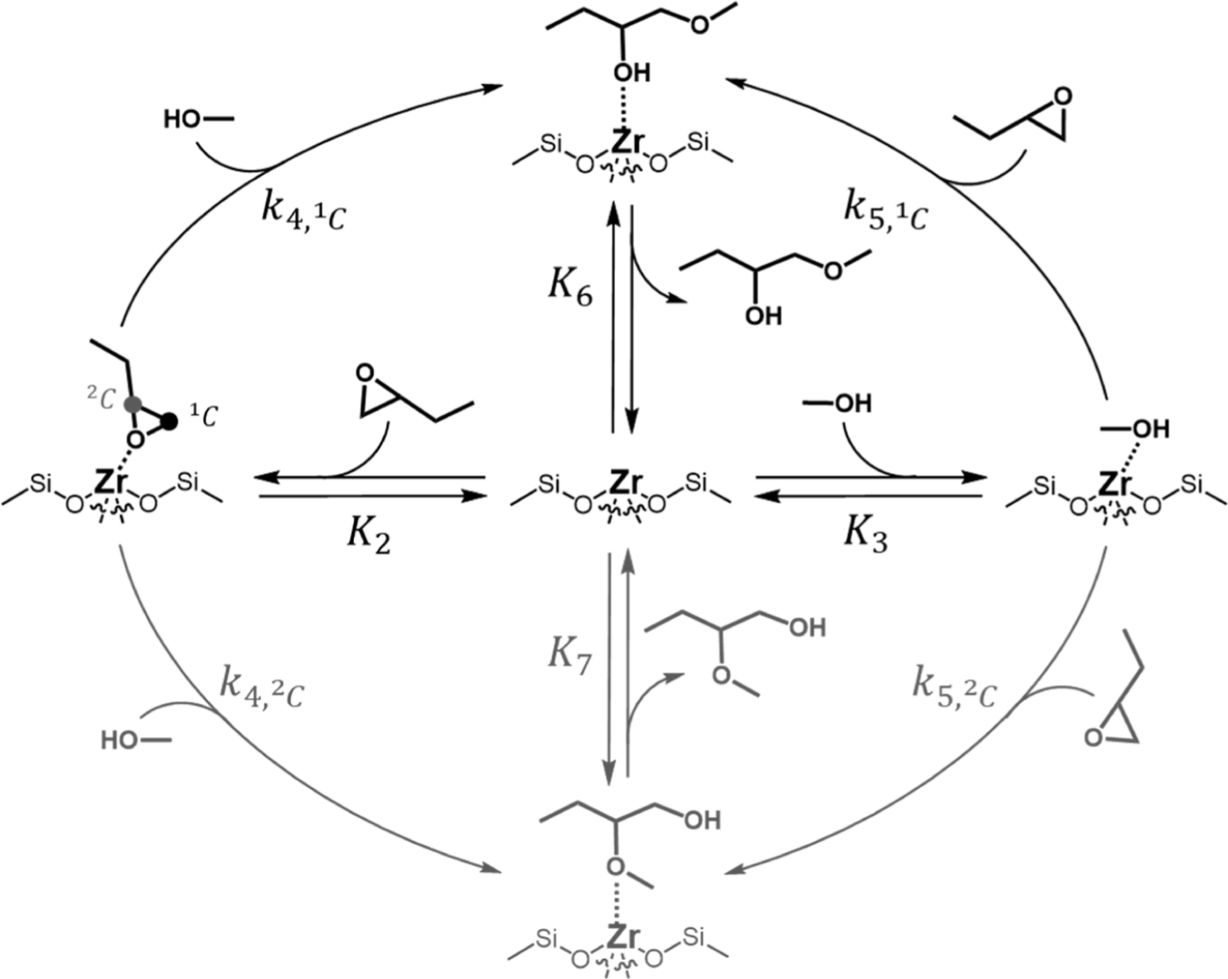
Proposed Catalytic Cycle for C_4_H_8_O Ring-Opening with CH_3_OH in CH_3_CN Solvent Over Zr-BEA Zeolites We expect an identical set of steps over the Al-BEA materials, although the structure of the intermediates differs due to proton transfer to the reactive species. All adsorption steps are currently assumed as reversible. Steps to form 1M2B and 2M1B are denoted as ^1^C or ^2^C, to signify nucleophilic attack on the primary and secondary carbons in the epoxide ring, respectively. For brevity, we do not show the reversible adsorption of CH_3_CN molecules (step 1) in this cycle. We portray only reactions with molecularly adsorbed CH_3_OH and C_4_H_8_O and not dissociatively bound oxygenates, although we expect each scenario may occur.

Scheme 2 presents a plausible system of elementary steps that explain the rate dependencies in Figures 1 and 2 over Zr-BEA materials (analogous set of steps for Al-BEA shown in Scheme S1). The cycle begins with the reversible adsorption of CH_3_CN (step 1, not shown for clarity), C_4_H_8_O (step 2), or CH_3_OH (step 3) to the active sites. The epoxide ring may open by the nucleophilic attack of bound C_4_H_8_O by CH_3_OH (step 4) or the reaction of bound CH_3_OH and a liquid phase C_4_H_8_O molecule (step 5). The distinct ring-opening products (C_5_H_12_O_2_—1M2B and 2M1B) desorb to complete the catalytic cycle (steps 6 and 7). Here, steps with a ^1^C subscript represent nucleophilic attack at the primary carbon in the C_4_H_8_O ring to form 1M2B, while a ^2^C subscript signifies attack at the secondary carbon to form 2M1B. 1M2B and 2M1B may form through either adsorbed intermediate by similar elementary steps that possess distinct rate constants (e.g., *k*_4,^1^C_, *k*_4,^2^C_) and transition state structures that drive changes in regioselectivity. The formation rates of 1M2B and 2M1B show very similar dependencies on [CH_3_OH] and [C_4_H_8_O] (see Section S5), supporting that the products form from common intermediates.

Rates for ring-opening (*r*_RO_) equal the sum of the rates of total product formation through bound C_4_H_8_O- (*r*_4_) or CH_3_OH-derived intermediates (*r*_5_)
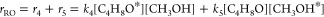
2where *k*_4_ and *k*_5_ represent the sum of the rate constants to form 1M2B and 2M1B in steps 4 (*k*_4,^1^C_, *k*_4,^2^C_) and 5 (*k*_5,^1^C_, *k*_4,^2^C_) in Scheme 2. [C_4_H_8_O*] and [CH_3_OH*] represent the number of adsorbed C_4_H_8_O and CH_3_OH intermediates, respectively. Applying the pseudo steady state hypothesis to the bound intermediates and deriving a site balance yields a turnover rate expression that accounts for reactions through bound C_4_H_8_O or CH_3_OH intermediates
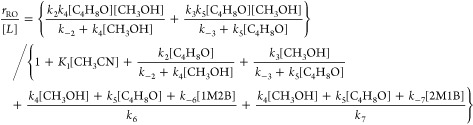
3

Section S6 provides a full derivation and discussion of the necessary assumptions to simplify the rate expression under conditions where C_4_H_8_O- or CH_3_OH-derived species saturate active sites and act as the most abundant reactive intermediate (MARI). Briefly, we utilize adsorption enthalpy measurements of C_4_H_8_O and CH_3_OH and the molar ratios of [CH_3_OH] to [C_4_H_8_O] to simplify eq 3 to yield the following rate expressions under conditions of C_4_H_8_O- (eq 4) and CH_3_OH-derived (eq 5) MARI species
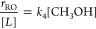
4
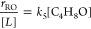
5

Equation 4 describes the rate trends in Figures 1 and 2 at lower molar ratios of [CH_3_OH] to [C_4_H_8_O] (<50 over Zr-BEA, <1200 over Al-BEA), while eq 5 matches trends at higher [CH_3_OH] to [C_4_H_8_O] ratios (>500 over Zr-BEA, >5000 over Al-BEA). The trends hold for both (SiOH)_*x*_-rich and (SiOH)_*x*_-poor materials, suggesting that each M-BEA material shares common reaction mechanisms and surface intermediates. Rate differences between materials instead likely stem from changes in the rate constants of the kinetically relevant nucleophilic attacks (*k*_4_ and *k*_5_).

Previous studies show that epoxide ring-opening rates and regioselectivities over zeolites depend strongly on the active metal and intrapore solvent structure. These variables likely drive the differences in *k*_4_ and *k*_5_ over Al-BEA and Zr-BEA materials through changes in the stability of reactive species. We postulate that turnover rate differences between Zr-BEA-OH and Zr-BEA-F reflect differences in noncovalent interactions with surrounding solvent molecules due to differences in (SiOH)_*x*_ density. Differences in *k*_4_ and *k*_5_ between Al-BEA and Zr-BEA likely stem from both the different charge transfer mechanisms of Lewis and Brønsted acid sites and the solvent environment surrounding these active sites. The following section quantitively examines the effect of these interactions on the stability of reactive species through activation barrier measurements.

### 3.2 Active Site Structure and Solvent Environment Drive Changes in Activation Barriers

The turnover rates for C_4_H_8_O ring-opening in Figures 1 and 2 depend on both the (SiOH)_*x*_ density of the *BEA framework and the active metal substituted within *BEA, stemming from changes in the values of *k*_4_ and *k*_5_ from the kinetically relevant steps shown in Scheme 2. The rate constants depend on the activation free energies (Δ*G*^‡^) and corresponding activation enthalpies (Δ*H*^‡^) and entropies (Δ*S*^‡^), according to transition state theory
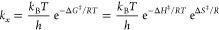
6

**Scheme 3:**
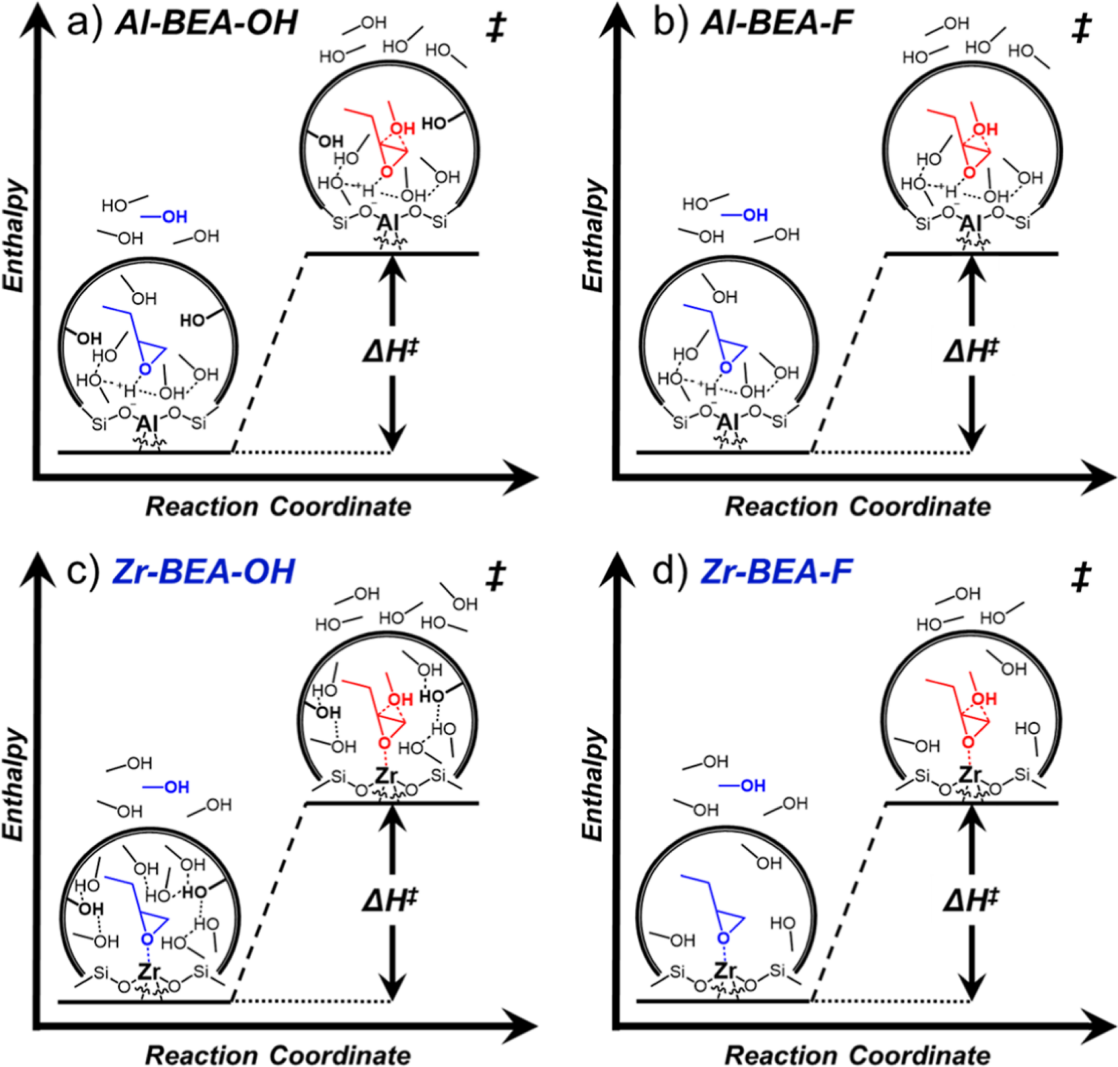
Proposed Reaction Coordinate Diagrams for C_4_H_8_O Ring-Opening with CH_3_OH in CH_3_CN Solvent under Conditions of a MARI Species Derived From C_4_H_8_O Over (a) Al-BEA-OH, (b) Al-BEA-F, (c) Zr-BEA-OH, and (d) Zr-BEA-F CH_3_OH adsorbs to react with C_4_H_8_O* and form the product, which desorbs to complete the reaction.

Scheme 3 depicts the proposed reaction coordinates for C_4_H_8_O ring-opening over (SiOH)_*x*_-rich and (SiOH)_*x*_-poor Zr-BEA and Al-BEA materials under a C_4_H_8_O-derived MARI. Values of Δ*H*^‡^ and Δ*S*^‡^ represent the difference between the energy of the transition state for nucleophilic attack and the sum of the energies of liquid-phase CH_3_OH and the bound C_4_H_8_O intermediate (C_4_H_8_O*)
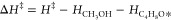
7
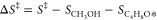
8

The activation parameters represent the total formation rate of both products. Both parameters consist of standard state (*H*^0^, *S*^0^) and excess (*H*^ε^, *S*^ε^) contributions
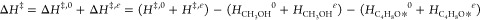
9
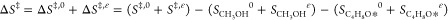
10

*H*^0^ and *S*^0^ values represent the covalent interactions stemming from the coordinate covalent bonds between Lewis acidic Zr sites and reactive species and the ionic interactions between the zeolite anion and protonated intermediates and transition states in the Brønsted acid material. *H*^ε^ and *S*^ε^ values encompass noncovalent interactions of the transition state or reactive intermediates with surrounding solvent molecules and the pore walls of the zeolite.^5,9^

**Figure 3:**
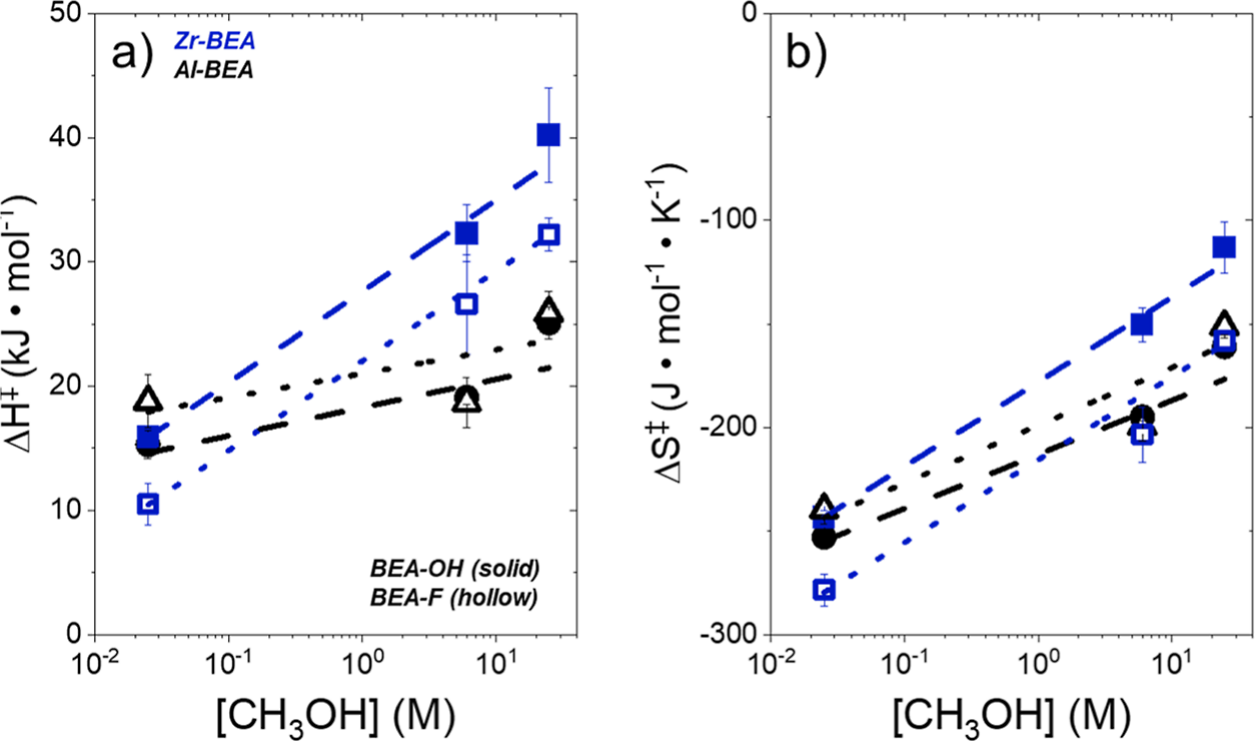
Activation (a) enthalpies and (b) entropies for C_4_H_8_O ring-opening with CH_3_OH (solid points −0.005 M C_4_H_8_O, 0.025 M CH_3_OH in CH_3_CN; half solid points −0.25 M C_4_H_8_O, 6 M CH_3_OH in CH_3_CN; hollow points −1 M C_4_H_8_O in neat (24.7 M) CH_3_OH), 298–323 K) over hydrophilic (solid points, dashed lines) and hydrophobic (hollow points, dotted lines) Zr- (blue) and Al-BEA (black) materials. Turnover rates obtained at additional concentrations (0.25 and 1 M C_4_H_8_O) are provided in Section S9.

Figure 3 presents Δ*H*^‡^ and Δ*S*^‡^ values as a function of [CH_3_OH] under conditions of a C_4_H_8_O-derived MARI species, corresponding to the rate expression described in eq 4. For all M-BEA, Δ*H*^‡^ and Δ*S*^‡^ values increase as a function of [CH_3_OH]. The value of *H*_CH_3_OH_^0^ does not vary as a function of [CH_3_OH] over a given M-BEA material, while calculated *H*_CH_3_OH_^ε^ values span a range of less than 3 kJ mol^–1^ across the range of [CH_3_OH] (see Section S7). Therefore, changes in the stability of reactive species within the zeolite pores (i.e., C_4_H_8_O*, transition state) drive the activation barrier differences with [CH_3_OH] in Figure 3. Increasingly positive Δ*H*^‡^ and Δ*S*^‡^ values at higher [CH_3_OH] suggest that the displacement of CH_3_OH molecules from the *BEA pores during transition state formation leads to a greater enthalpic cost and corresponding entropic gain than the displacement of CH_3_CN, consistent with our previous interpretations of epoxide ring-opening^27^ and alkene epoxidation^11^ activation barriers over M-BEA materials.

As [CH_3_OH] increases from 0.025 to 24.7 M CH_3_OH, the Al-BEA materials show less significant increases in Δ*H*^‡^ (7–10 kJ mol^–1^) and Δ*S*^‡^ (88–92 J mol^–1^ K^–1^) than the Zr-BEA materials (22–25 kJ mol^–1^, 120–130 J mol^–1^ K^–1^). The different dependences of these energies on [CH_3_OH] stem from both standard state and excess contributions. The protonation of the transition state and adsorbed C_4_H_8_O in Al-BEA likely influences Δ*H*^‡,0^ and Δ*S*^‡,0^ values, leading to differences between Al- and Zr-BEA materials. Furthermore, the proton that comprises the active site within Al-BEA delocalizes across clusters of CH_3_OH molecules^54−56^ at higher [CH_3_OH] values (24.7 M and possibly 6 M, illustrated in Scheme 3). The delocalization and solvation of the proton may lower Δ*H*^‡^ through either an enthalpic stabilization of the transition state (increasing *H*^‡^ in eq 7) or an enthalpic destabilization of C_4_H_8_O* (increasing *H*_C_4_H_8_O_*). We hypothesize that the delocalization of the active site increases *H*_C_4_H_8_O_* but leads to a corresponding increase in *S*_C_4_H_8_O_* by increasing the mobility of this adsorbed intermediate, as supported by more endothermic C_4_H_8_O adsorption enthalpies in CH_3_OH than CH_3_OH (see Section 3.4). These changes may arise from a combination of covalent and noncovalent interactions. Despite the convoluting effects of active site solvation in Al-BEA, the increasing trends of Δ*H*^‡^ and Δ*S*^‡^ with [CH_3_OH] for all M-BEA shows the strong effect of noncovalent interactions on the activation barriers through changes in Δ*H*^‡,ε^ and Δ*S*^‡,ε^.

The (SiOH)_*x*_ density of M-BEA can also influence Δ*H*^‡^ and Δ*S*^‡^ values for C_4_H_8_O ring-opening. Al-BEA-OH and Al-BEA-F provide similar values of Δ*H*^‡^ (within 1–4 kJ mol^–1^) and Δ*S*^‡^ (within 5–10 J mol^–1^ K^–1^) at each [CH_3_OH] value. The differences in Δ*H*^‡^ and Δ*S*^‡^ largely compensate to provide very similar rates over Al-BEA-OH and Al-BEA-F (Figures 1 and 2), as Δ*G*^‡^ values at 308 K fall within 1 kJ mol^–1^ at each [CH_3_OH] for these materials. In contrast, Zr-BEA-OH shows more positive Δ*H*^‡^ and Δ*S*^‡^ values than Zr-BEA-F by 5–8 kJ mol^–1^ and 35–55 J mol^–1^ K^–1^, respectively. Zr-BEA-OH provides Δ*G*^‡^ values 5–6 kJ mol^–1^ less than Zr-BEA-F (308 K), demonstrating that entropic gains lead to greater turnover rates in Zr-BEA-OH. The presence of (SiOH)_*x*_ groups plausibly alters Δ*H*^‡,ε^ and Δ*S*^‡,ε^ through changes in the solvent structure surrounding the active sites, as illustrated in Scheme 3. The H^+^ sites in the Al-BEA materials likely govern the solvent structure surrounding C_4_H_8_O* and the ring-opening transition state, leading to similar solvent configurations around the active sites in Al-BEA-OH (Scheme 3a) and Al-BEA-F (Scheme 3b). Therefore, the H^+^ sites may lessen the effect of (SiOH)_*x*_ on the solvent environment near active sites, giving similar Δ*H*^‡^ and Δ*S*^‡^ between Al-BEA-OH and Al-BEA-F. In comparison, the stronger influence of (SiOH)_*x*_ on Δ*H*^‡^ and Δ*S*^‡^ for Zr-BEA materials implies a more significant role of (SiOH)_*x*_ on the solvent structure surrounding the Zr sites. Zr-BEA-OH likely adsorbs greater quantities of CH_3_OH and CH_3_CN that form hydrogen bond networks within the pores (Scheme 3c), while the pores of Zr-BEA-F primarily contain weakly interacting CH_3_OH and CH_3_CN molecules (Scheme 3d). These hypotheses align with previous proposals for the structural differences of H_2_O,^12,14,57^ CH_3_CN,^57,58^ and alcohol^10,11,59^ molecules within hydrophilic and hydrophobic Lewis acid zeolites. The formation of the ring-opening transition state likely requires the disruption of hydrogen bonds between solvent molecules within Zr-BEA-OH that leads to greater Δ*H*^‡^ and Δ*S*^‡^ values compared to Zr-BEA-F at all [CH_3_OH]. The entropic gains exceed the enthalpic penalty, leading to greater rates for ring-opening in Zr-BEA-OH than Zr-BEA-F. These trends align with previous observations for alkene epoxidation with H_2_O_2_ in organic solvents (CH_3_OH, CH_3_CN) containing H_2_O over Ti-BEA-OH and Ti-BEA-F,^11,13,19,20,57,58^ providing evidence that the intrapore solvent structure influences alkene epoxidation and the secondary epoxide ring-opening process in similar ways.

Figure 3 shows that differences in metal identity, solvent composition, and (SiOH)_*x*_ density lead to differences in rate constants for epoxide ring-opening through changes in Δ*H*^‡^ and Δ*S*^‡^, which stem from changes in the stability of reactive species in the zeolite pores. The distinct dependences of Δ*H*^‡^ and Δ*S*^‡^ on (SiOH)_*x*_ density over Al-BEA and Zr-BEA materials imply that different contributions govern the intrapore solvent structure within these Brønsted and Lewis acid materials. The following section establishes the differences in solvent structure among M-BEA-OH and M-BEA-F that drive rate differences for ring-opening.

### 3.3 Solvent Environment Governed by Different Functions in Brønsted and Lewis Acid Zeolites

Recent studies show that the intrapore composition of binary liquid mixtures in zeolites depends on the liquid solvent composition,^11,60−62^ (SiOH)_*x*_ density within Lewis acid^11^ and siliceous zeolites,^15,63,64^ and the density of H^+^ sites within Brønsted acids.^15,34,65^ We hypothesize that differences in the intrapore solvent composition will correlate strongly with observed trends in rates and activation barriers described above.

**Figure 4:**
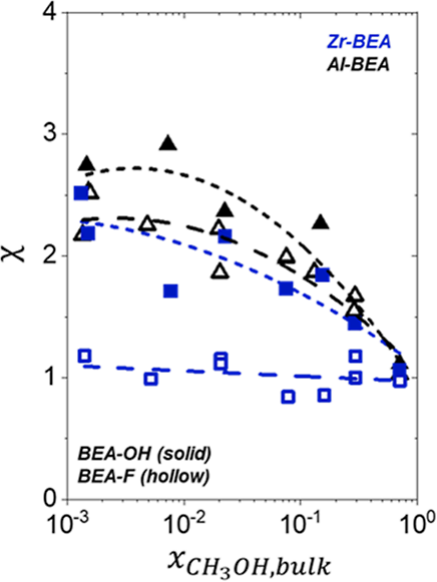
Preferential absorption of CH_3_OH within zeolite pores expressed as values of χ as a function of *x*_CH_3_OH,bulk_ at 308 K in a mixture of CH_3_OH and CH_3_CN over hydrophilic (solid) and hydrophilic (hollow) Al- (black) and Zr-BEA (blue) materials.

Figure 4 demonstrates that the solvent composition within M-BEA depends on the active metal identity, (SiOH)_*x*_ density, and mole fraction of CH_3_OH within CH_3_CN in the bulk solvent (*x*_CH_3_OH,bulk_). Here, χ represents the ratio of the estimated mole fractions of CH_3_OH in the zeolite pores (*x*_CH_3_OH,pore_) and *x*_CH_3_OH,bulk_ at equilibrium (full experiment and calculation details provided in Section S10)
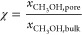
11

The M-BEA-OH materials in Figure 4 consistently show higher χ values than the corresponding M-BEA-F material, suggesting that (SiOH)_*x*_ groups facilitate CH_3_OH adsorption relative to CH_3_CN. CH_3_OH contains a greater sum of hydrogen bond donors and acceptors (3) than CH_3_CN (1), meaning that CH_3_CN molecules contain fewer functions to coordinate with (SiOH)_*x*_ groups or form hydrogen-bonding networks with other molecules. Values of χ generally decrease with increasing *x*_CH_3_OH,bulk_ over each M-BEA material, with the M-BEA-OH and M-BEA-F materials collapsing to more similar values at higher *x*_CH_3_OH,bulk_. These trends suggest that (SiOH)_*x*_ functions influence CH_3_OH uptake more strongly at lower *x*_CH_3_OH,bulk_, when lower quantities of CH_3_OH reside in the pores. This interpretation appears consistent with previous observations in which H_2_O uptake within hydrophilic zeolites from mixtures with ethanol^66,67^ and acetic acid^68^ depend more strongly on *x*_H_2_O,bulk_ at lower *x*_H_2_O,bulk_ values. At higher mole fractions, CH_3_OH molecules can likely associate with preadsorbed CH_3_OH within the pores, leading to a weaker influence of the (SiOH)_*x*_ density or active site structure on CH_3_OH adsorption.

Interestingly, Al-BEA-OH and Al-BEA-F show more similar χ values than Zr-BEA-OH and Zr-BEA-F across the range of *x*_CH_3_OH,bulk_. The greater dependence of χ values on (SiOH)_*x*_ density over Zr-BEA materials provides strong evidence that (SiOH)_*x*_ groups carry a greater influence on the intrapore solvent structure within Zr-BEA than Al-BEA materials. While Al-BEA-OH generally shows greater χ than Al-BEA-F, the similar trends and magnitudes of χ across *x*_CH_3_OH,bulk_ for these materials suggest that the H^+^ sites within Al-BEA impact the intrapore solvent structure more strongly than the framework Zr active sites in Zr-BEA. This explanation agrees with previous works that examined H_2_O adsorption over Brønsted acid materials, which found that H_2_O preferentially coordinates at H^+^ at lower coverages before binding to (SiOH)_*x*_ groups after saturating the H^+^ sites.^34,46,65^ CH_3_OH molecules may show a similar binding preference to the H^+^ sites in Al-BEA materials here. The observations from Figure 4 align with the interpretations of Δ*H*^‡^ and Δ*S*^‡^ values discussed in the previous section and the hypothesized differences in solvent structure among M-BEA illustrated in Scheme 3 above. The active sites in Al-BEA-OH and Al-BEA-F likely promote similar surrounding configurations of solvent molecules, while the difference in density of (SiOH)_*x*_ groups between Zr-BEA-OH and Zr-BEA-F leads to dissimilar solvent structures around the active sites. The distinct solvent structures within these materials likely drive the rate and activation barrier differences described in the previous sections.

**Figure 5:**
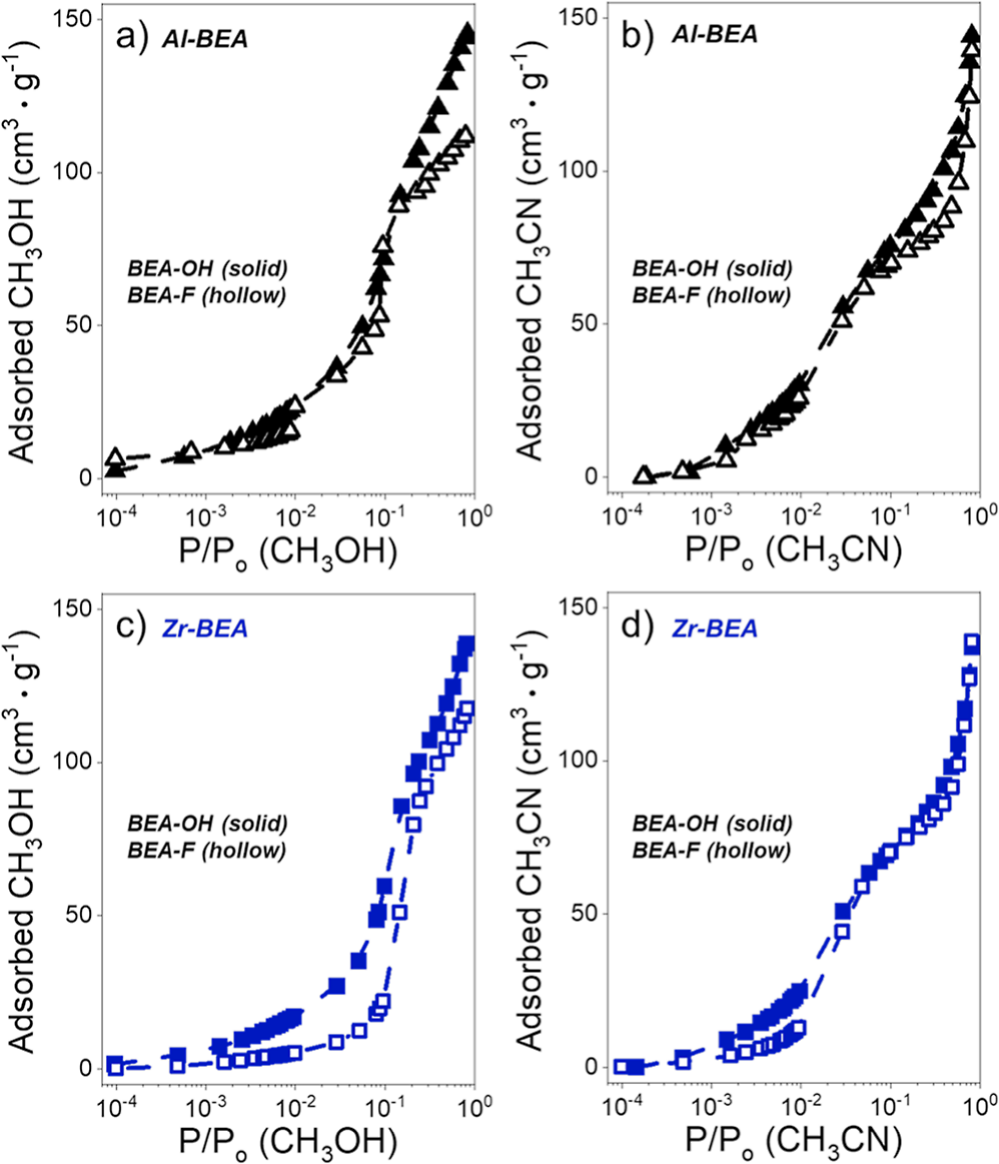
(a) CH_3_OH (293 K) and (b) CH_3_CN (296 K) adsorption isotherms over hydrophilic (solid) and hydrophilic (hollow) Al- (black) and Zr-BEA (blue) materials.

While the χ values shown in Figure 4 give insight into the intrapore solvent compositions in M-BEA during C_4_H_8_O ring-opening, these data do not probe the absolute density of solvent within the pores. Figure 5 presents single-component vapor adsorption isotherms for CH_3_OH and CH_3_CN over M-BEA, revealing that the (SiOH)_*x*_ density and active site structure influence the density of solvent molecules within each M-BEA material. The ratios of uptakes between Al-BEA-OH and Al-BEA-F vary from 0.9 to 1.2 for CH_3_CN and 1.0 to 1.5 for CH_3_OH at relative pressures (*P*/*P*_0_) less than 0.05. In contrast, Zr-BEA-OH uptakes differ significantly, ranging from 1.5 to 2.4 times more CH_3_CN and 2.7 to 5.1 times more CH_3_OH than Zr-BEA-F when *P*/*P*_0_ falls below 0.05. The similar uptakes of both adsorbates for the Al-BEA materials support that CH_3_CN and CH_3_OH bind preferentially to H^+^ sites. The greater uptakes over Zr-BEA-OH than Zr-BEA-F indicate that the adsorbates coordinate to different framework locations; CH_3_CN and CH_3_OH likely bind preferentially to the (SiOH)_*x*_ nests within Zr-BEA-OH, while CH_3_CN and CH_3_OH should primarily bind to framework Zr atoms or Si–O–Si linkages within Zr-BEA-F.

As P/P_0_ values surpass 0.05 and the pores begin to fill, the M-BEA-OH and M-BEA-F materials reach more similar uptakes of CH_3_OH and CH_3_CN regardless of acid type. For example, when *P*/*P*_0_ equals 0.8, M-BEA-OH and M-BEA-F adsorb nearly identical quantities of CH_3_CN, while the M-BEA-OH materials adsorb 1.3 times more CH_3_OH than M-BEA-F. Comparisons between Zr-BEA and Al-BEA materials of similar (SiOH)_*x*_ density show the total uptake differs by less than 10% at *P*/*P*_0_ of 0.8. These similarities suggest that as the adsorbates coordinate to less favorable binding sites and begin to form extended networks within the pore, the M-BEA materials reach similar densities of CH_3_OH and CH_3_CN regardless of the intrapore (SiOH)_*x*_ density or active site character.

Nevertheless, the differences in vapor uptake at the lowest P/P_0_ values (i.e., *P*/*P*_0_ < 0.05; Figure 5) show that the solvent environments surrounding the Brønsted acid sites depend weakly on nearby (SiOH)_*x*_ groups. In contrast, the (SiOH)_*x*_ nests within Zr-BEA-OH promote hydrogen-bonding structures of CH_3_CN and CH_3_OH near Zr active sites that do not appear within Zr-BEA-F pores. Furthermore, M-BEA-OH and M-BEA-F materials show greater differences in CH_3_OH than CH_3_CN uptake across the full range of *P*/*P*_0_, demonstrating that (SiOH)_*x*_ nests carry a greater influence on the adsorption of CH_3_OH. These findings agree with the interpretation of χ values in Figure 4. Overall, the comparisons of single-component vapor uptakes (Figure 5) further indicate that the H^+^ sites structure solvent similarly within Al-BEA-OH and Al-BEA-F, while the difference in (SiOH)_*x*_ density leads to differences in the solvent structure surrounding the catalytic active sites in Zr-BEA-OH and Zr-BEA-F.

Figures 4 and 5 give strong evidence that Lewis and Brønsted acid zeolites promote distinct intrapore solvent structures that depend differently on the (SiOH)_*x*_ density of the zeolite pores. Tuning the liquid solvent composition, active site structure, and (SiOH)_*x*_ density provides opportunities to control the solvent composition and density within porous catalysts. The solvent environment within M-BEA pores significantly influences C_4_H_8_O ring-opening rates and likely impacts the thermodynamics and selectivities of these processes, as highlighted in the following sections.

### 3.4 Enthalpies of 1,2-Epoxybutane Adsorption and Correlations to Apparent Barriers Show Influence of Intrapore Environment on Stability of Reactive Species

The Brønsted–Evans–Polanyi model proposes that the enthalpy of reaction for an elementary step scales linearly with the intrinsic activation energy of the same step, therefore establishing a connection between the intrinsic kinetics and thermodynamics of a reaction.^69−71^ Hammond later explained this connection by postulating that the transition state for a reaction resembles the stable state (i.e., reactant or product) most similar in energy.^72,73^ Recent studies demonstrate that the liquid-phase adsorption enthalpies for epoxide molecules over *BEA zeolites correlate positively with Δ*H*^‡^ values for alkene epoxidation as a function of the active metal,^35^ (SiOH)_*x*_ density,^43,57^ and solvent composition.^11^ More recently, we established linear correlations between epoxide adsorption enthalpies (Δ*H*_ads,C_4_H_8_O_) and Δ*H*^‡^ values for C_4_H_8_O ring-opening over M-BEA materials, implicating a transition state for ring-opening that resembles the adsorbed C_4_H_8_O reactant.^27^ The measurement of Δ*H*_ads,C_4_H_8_O_ can provide insight into the differences in ring-opening transition state stability over M-BEA-OH and M-BEA-F materials.

**Figure 6:**
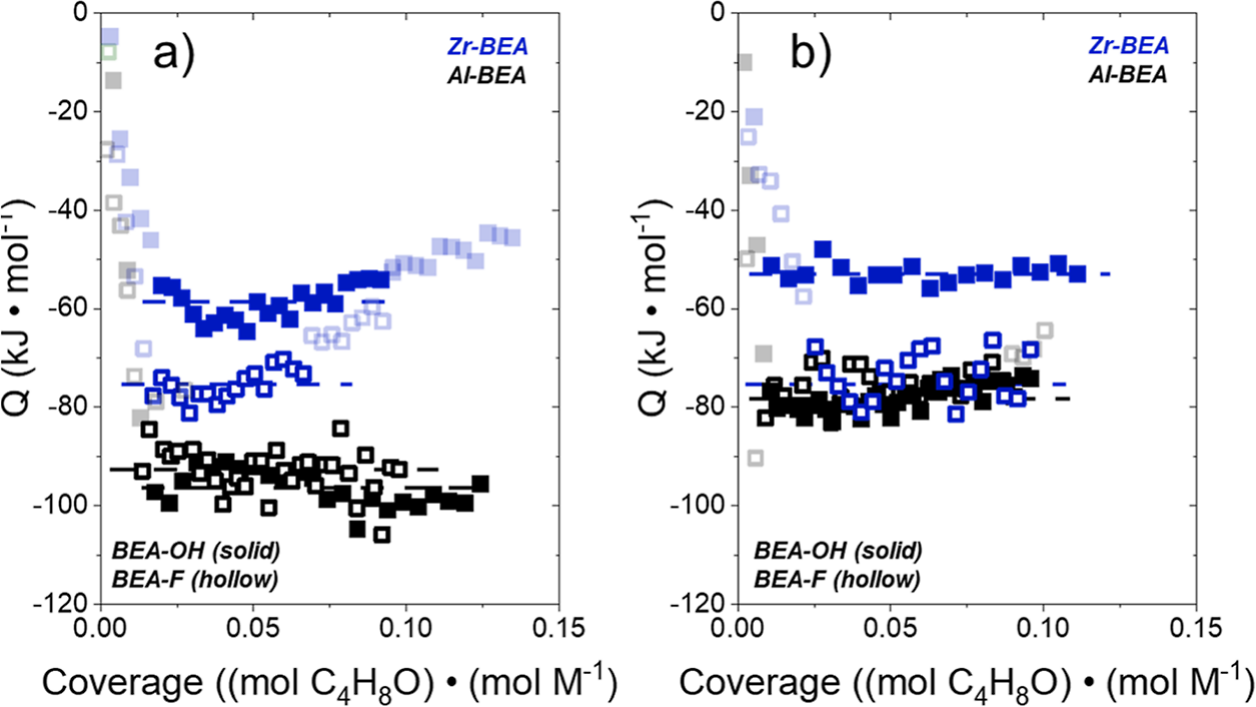
Heat released upon C_4_H_8_O adsorption in (a) CH_3_CN and (b) CH_3_OH as a function of C_4_H_8_O to metal ratio over hydrophilic (solid) and hydrophilic (hollow) Al- (black) and Zr-BEA (blue) materials (0.005 M C_4_H_8_O, 308 K). Transparent points are omitted from the adsorption enthalpy calculation, denoted by the dashed line. Section S11 includes adsorption enthalpy calculation details, raw data for the ITC experiments from Figure 6, and further ITC measurements, including CH_3_OH adsorption over each M-BEA.

Figure 6 reveals that Δ*H*_ads,C_4_H_8_O_ measured by isothermal titration calorimetry (ITC) depends on the active metal identity, solvent choice, and density of (SiOH)_*x*_ groups within M-BEA. The Al-BEA materials show more exothermic Δ*H*_ads,C_4_H_8_O_ values than the Zr-BEA materials in a CH_3_CN-rich solvent, suggesting that C_4_H_8_O adsorbs more strongly to the Brønsted acid sites than to Zr framework sites. This trend corresponds with the weaker dependence of rates on [C_4_H_8_O] in Al-BEA than Zr-BEA materials (Figure 2) and gives insight into why C_4_H_8_O saturates sites on Al-BEA across nearly all conditions examined. Δ*H*_ads,C_4_H_8_O_ values over Zr-BEA-OH and Zr-BEA-F change negligibly upon changing the solvent from CH_3_CN and CH_3_OH, while CH_3_OH provides Δ*H*_ads,C_4_H_8_O_ values ∼15 kJ mol^–1^ more endothermic than CH_3_CN over both Al-BEA-OH and Al-BEA-F. The more endothermic Δ*H*_ads,C_4_H_8_O_ for Al-BEA in CH_3_OH may stem from the solvation of H^+^ by CH_3_OH, which could influence the binding enthalpy of C_4_H_8_O to the H^+^ sites. However, we also cannot exclude that the reaction of C_4_H_8_O and CH_3_OH may contribute to the measured Δ*H*_ads,C_4_H_8_O_ in CH_3_OH over both Zr-BEA and Al-BEA materials.

In both CH_3_CN and CH_3_OH, Δ*H*_ads,C_4_H_8_O_ values vary by less than 5 kJ mol^–1^ between Al-BEA-OH and Al-BEA-F. In contrast, Zr-BEA-OH shows a more endothermic Δ*H*_ads,C_4_H_8_O_ than Zr-BEA-F by 17–21 kJ mol^–1^ in both solvents. The similar Δ*H*_ads,C_4_H_8_O_ for the Al-BEA materials suggests that C_4_H_8_O similarly reorganizes the solvent surrounding the H^+^ sites in Al-BEA-OH and Al-BEA-F during adsorption. This observation provides strong evidence that the Al-BEA materials stabilize similar solvent structures around the H^+^ active sites, which leads to similar behavior between Al-BEA-OH and Al-BEA-F during C_4_H_8_O ring-opening, solvent adsorption, and C_4_H_8_O adsorption. The more endothermic Δ*H*_ads,C_4_H_8_O_ for Zr-BEA-OH compared to Zr-BEA-F likely originates from the disruption of hydrogen-bonded solvent molecules (CH_3_OH or CH_3_CN) by adsorbing C_4_H_8_O within the pores of Zr-BEA-OH. The displacement of weakly interacting solvent molecules within Zr-BEA-F requires a smaller enthalpic penalty and gives more exothermic Δ*H*_ads,C_4_H_8_O_ values.

**Figure 7:**
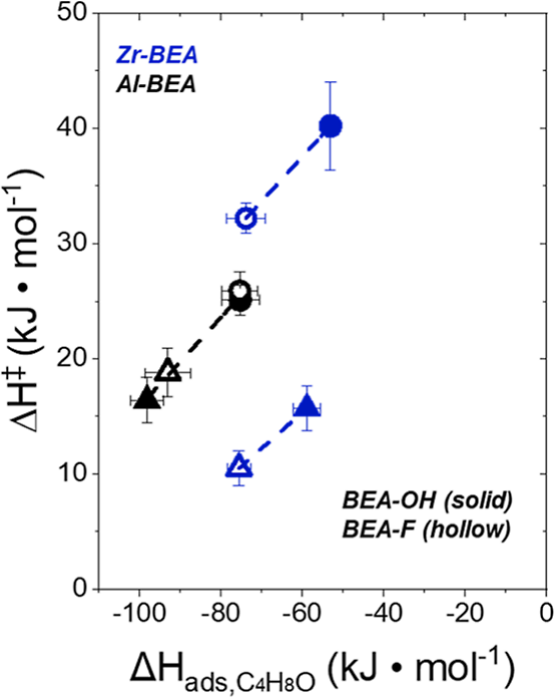
Δ*H*^‡^ values for C_4_H_8_O ring-opening (from Figure 3, 0.025 M CH_3_OH in CH_3_CN (triangles) or 24.7 M CH_3_OH (circles)) as a function of Δ*H*_ads_ for C_4_H_8_O over hydrophilic (solid) and hydrophilic (hollow) Al- (black) and Zr-BEA (blue) materials.

Figure 7 shows that Δ*H*^‡^ and Δ*H*_ads,C_4_H_8_O_ values correlate linearly for Al-BEA and Zr-BEA materials as a function of (SiOH)_*x*_ density at a given solvent composition. Interestingly, the Al-BEA and Zr-BEA materials do not fall on the same trendline. These differences likely originate from the proton transfer to the C_4_H_8_O* and ring-opening transition state or the delocalization of H^+^ by CH_3_OH, which may convolute the comparisons between Brønsted and Lewis acids. Furthermore, changing the solvent from CH_3_CN-rich to neat CH_3_OH leads to a discontinuity in the trend for the Zr-BEA materials but not for the Al-BEA materials in Figure 7. We postulate that the enthalpy of reaction between C_4_H_8_O and CH_3_OH may contribute to Δ*H*_ads,C_4_H_8_O_ and contribute to the discontinuity of the Zr-BEA materials (i.e., following adsorption of C_4_H_8_O, the adsorbed epoxide ring opens by reaction with CH_3_OH in the calorimeter). The solvation of H^+^ by CH_3_OH in Al-BEA apparently prevents or offsets this reaction contribution and leads to a consistent trend across all points. Section S12 includes a modified version of the enthalpy relationship from Figure 7 that accounts for the possible contribution of the enthalpy of reaction to Δ*H*_ads,C_4_H_8_O_. Regardless of the reaction potentially convoluting Δ*H*_ads,C_4_H_8_O_ contributions between CH_3_CN and CH_3_OH, the linear correlations between Δ*H*^‡^ and Δ*H*_ads,C_4_H_8_O_ over Zr-BEA and Al-BEA at a given solvent composition provide strong evidence that the adsorption of C_4_H_8_O and the formation of the C_4_H_8_O ring-opening transition state depend similarly on the surrounding solvent structure.

Al-BEA-OH and Al-BEA-F show very similar Δ*H*^‡^ and Δ*H*_ads,C_4_H_8_O_ within both solvent compositions, giving further evidence that the difference in (SiOH)_*x*_ density between these materials does not yield differences in the intrapore solvent structure that drive differences in catalysis or adsorption. The more endothermic values of each enthalpy at a higher [CH_3_OH] likely originate from a greater disruption of hydrogen-bonding solvent molecules during the formation of the ring-opening transition state and the adsorption of C_4_H_8_O within CH_3_OH-rich solvents. Δ*H*_C_4_H_8_O_^ε^ values vary by less than 3 kJ mol^–1^ across the range of [CH_3_OH] studied (Section S7), demonstrating that changes in Δ*H*_ads,C_4_H_8_O_ between solvents in Figure 7 result from changes in the stability of C_4_H_8_O* rather than liquid-phase C_4_H_8_O. Significantly, the enthalpic penalty for forming the transition state in Scheme 3a,b must exceed that for C_4_H_8_O adsorption to cause an increase in Δ*H*^‡^ with [CH_3_OH]; in other words, the excess enthalpy increases for the transition state (*H*^‡,ε^) must exceed that of the reference state for ring-opening (*H*_C_4_H_8_O*_^ε^) (eq 9). The transition state likely occupies more space than the C_4_H_8_O* intermediate and requires a more significant displacement of surrounding solvent molecules, which may yield a more significant excess enthalpic penalty.

The disruption of hydrogen-bonded solvent also appears to impart a greater enthalpic penalty to the larger transition state in the Zr-BEA materials. However, Zr-BEA-OH shows more endothermic Δ*H*^‡^ and Δ*H*_ads,C_4_H_8_O_ values than Zr-BEA-F in both CH_3_CN-rich and neat CH_3_OH solvents. The inability of solvent molecules to form hydrogen-bonding networks in the pores of Zr-BEA-F likely lessens the enthalpic cost required to form the ring-opening transition state or adsorb C_4_H_8_O to the Zr sites. Notably, the slope of the correlation between Zr-BEA materials in Figure 7 becomes more positive in neat CH_3_OH compared to a CH_3_CN-rich solvent. The steeper slope of the Zr-BEA correlations in neat CH_3_OH likely occurs because *H*^‡,ε^ depends more strongly on the (SiOH)_*x*_ density within Zr-BEA than *H*_C_4_H_8_O*_^ε^ in the presence of CH_3_OH than CH_3_CN. Specifically, the difference in enthalpic penalties provided to the larger transition state and smaller C_4_H_8_O* becomes more significant when the pores of Zr-BEA-OH contain hydrogen-bonded CH_3_OH compared to hydrogen-bonded CH_3_CN. Overall, the different dependences of Δ*H*^‡^ and Δ*H*_ads,C_4_H_8_O_ on (SiOH)_*x*_ density between Al-BEA and Zr-BEA materials reinforces that the solvent structure within the *BEA pores depends on different functions within Brønsted and Lewis acid zeolites. The H^+^ sites likely govern the solvent environment surrounding active sites within Al-BEA-OH and Al-BEA-F, while the higher density of (SiOH)_*x*_ groups within Zr-BEA-OH likely leads to a significantly different solvent environment around the Zr active sites than in Zr-BEA-F.

The solvent structure within M-BEA pores appears to drive differences in ring-opening turnover rates and activation barriers through changes in the excess energies of reactive species, as described above. The following section examines the consequences of these excess interactions and the covalent interactions between the reactive species and active sites on the regioselectivities of C_4_H_8_O ring-opening.

### 3.5 Active Site Structure and Solvation Drive Changes in Ring-Opening Regioselectivity

The interactions between transition states and intermediates with active and interactions with surrounding solvent molecules within zeolites influence rates and barriers for epoxide ring-opening. The distribution of regioisomers for C_4_H_8_O ring-opening with CH_3_OH over M-BEA (see Scheme 1 above) also depends on these intrapore interactions, as shown in our previous work.^27^ The regioselectivities are quantified with a rate ratio defined as β
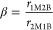
12where *r*_1M2B_ and *r*_2M1B_ represent the formation rates of 1M2B and 2M1B, respectively. The formation rates depend on the reactant concentrations and product-specific rate constants for the kinetically relevant steps (*k*_4,^1^*C*_, *k*_4,^2^*C*_, *k*_5,^1^*C*_, *k*_5,^2^*C*_) (Scheme 2). The expression for β simplifies if we assume the products form from identical surface intermediates and fluid-phase reactants upon the same set of active sites and we consider only conditions of C_4_H_8_O*-covered active sites (full derivation and discussion of assumptions in Section S6). In this case, β solely depends on the difference between the free energies of the transition states for the individual products (i.e., *G*_1M2B_^‡^ and *G*_2M1B_^‡^)
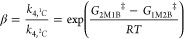
13

**Figure 8:**
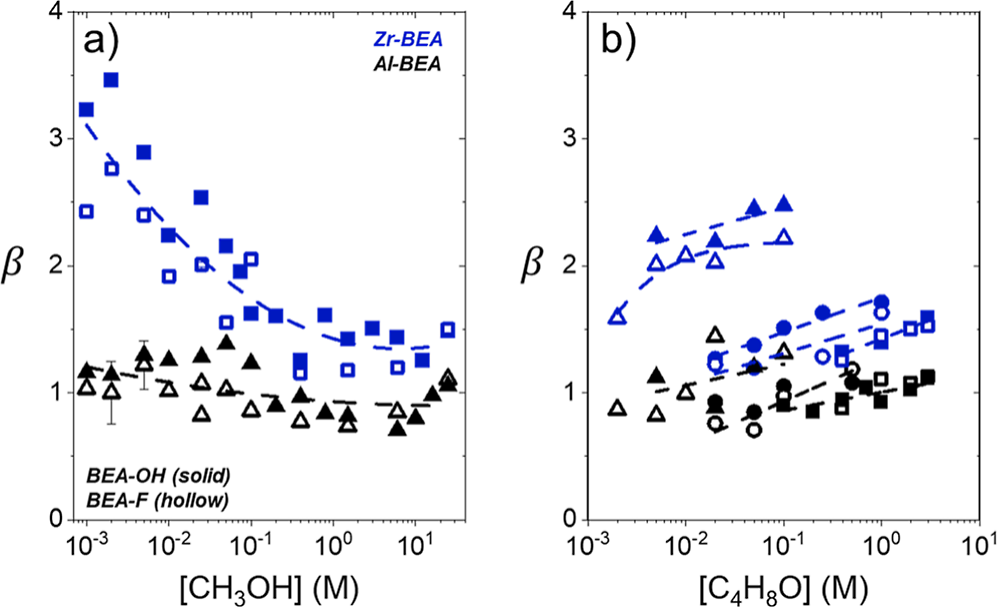
Measures of regioselectivity quantified by values of β for C_4_H_8_O ring-opening with CH_3_OH as a function of (a) CH_3_OH concentration (0.005–1 M C_4_H_8_O, [CH_3_OH]/[CH_3_CN] = 0.2–100, CH_3_CN solvent, 308 K), and (b) C_4_H_8_O concentration at 0.025 M (triangles), 6 M (circles), or 24.7 M CH_3_OH (squares) (CH_3_CN, 308 K) over hydrophilic (solid) and hydrophilic (hollow) Al- (black) and Zr-BEA (blue). All measurements were made under C_4_H_8_O*-covered active sites.

Figure 8 presents β values as a function of reactant concentrations under conditions of C_4_H_8_O*-covered active sites, which depend on the liquid solvent composition and the active metal and (SiOH)_*x*_ density within the *BEA pores. β values decrease at greater [CH_3_OH] for the Zr-BEA materials (Figure 8a), with β values approaching 3–3.5 at the lowest [CH_3_OH] (<0.1 M CH_3_OH) and β values of 1–1.5 in CH_3_OH-rich solvents. The β values over Al-BEA-OH and Al-BEA-F change less strongly with [CH_3_OH], with values between 0.75 and 1.5 across the range of [CH_3_OH]. Figure 8b shows that β values increase slightly with increasing [C_4_H_8_O] across each M-BEA at several values of [CH_3_OH] (0.025, 6, and 24.7 M). The generally greater regioselectivities to the terminal ether (1M2B) compared to the terminal alcohol (2M1B) in Figure 8 aligns with prior studies, which commonly attribute the difficulty of forming the terminal alcohol to the steric hindrance for nucleophilic attack at the more substituted C-atom of the oxirane ring.^74−77^ However, Figure 8 also reveals that increasing the number of hydrogen bond donors and acceptors within the solvent by increasing [CH_3_OH] leads to a greater preference to form 2M1B, which agrees with a recent report showing that intentionally adding hydrogen-bonding acceptors to the system (e.g., diols) leads to greater selectivity to terminal alcohols during epoxide ring-opening with alcohols over homogeneous borane catalysts.^78^ CH_3_OH molecules may preferentially stabilize the 2M1B transition state relative to 1M2B through hydrogen-bonding interactions within M-BEA, as evidenced by the increasing enthalpic preference to form 2M1B at higher [CH_3_OH] that corresponds to lower β values (see Section S8 for comparisons of the activation barriers to form the products). At lower [CH_3_OH] values, CH_3_CN molecules likely interrupt CH_3_OH from forming hydrogen bonds, thus contributing to the greater preference toward the TE product.

The weaker dependence of β values on reaction composition over Al-BEA compared to Zr-BEA materials in Figure 8 suggests that the intrapore solvent environment plays a lesser role in regioselectivity trends over the Brønsted acids. Instead, the covalent interactions between the H^+^ sites and reactive species appear to drive the distribution of regioisomers over Al-BEA. At an equivalent reaction composition, the Al-BEA materials consistently show lower β values than the Zr-BEA materials. Recent quantum chemical calculations reveal that protonation of the epoxide causes elongation of the weaker C–O bond between the more substituted carbon and epoxide oxygen atom,^79^ thereby facilitating the nucleophilic attack at this carbon to form the terminal alcohol.^79−82^ These interactions between the epoxide ring, H^+^ site, and the attacking CH_3_OH molecule likely lead to β values near unity in all conditions. A previous study reported β values from 0.8 to 1.3 for C_4_H_8_O ring-opening in neat CH_3_OH catalyzed by sulfuric acid and Brønsted acid zeolites of varying pore size, agreeing with the span of values for Al-BEA in Figure 8.^83^ Homogeneous (i.e., aluminum triflate)^84,85^ and heterogeneous Lewis acids (i.e., Hf-, Sn-, Zr-BEA)^75^ previously achieved β values between 1.2 and 1.4 for C_4_H_8_O ring-opening in neat CH_3_OH. Figure 8 demonstrates that introducing an organic cosolvent (i.e., CH_3_CN) provides opportunities to extend to higher β values over Lewis acidic materials.

**Scheme 4:**
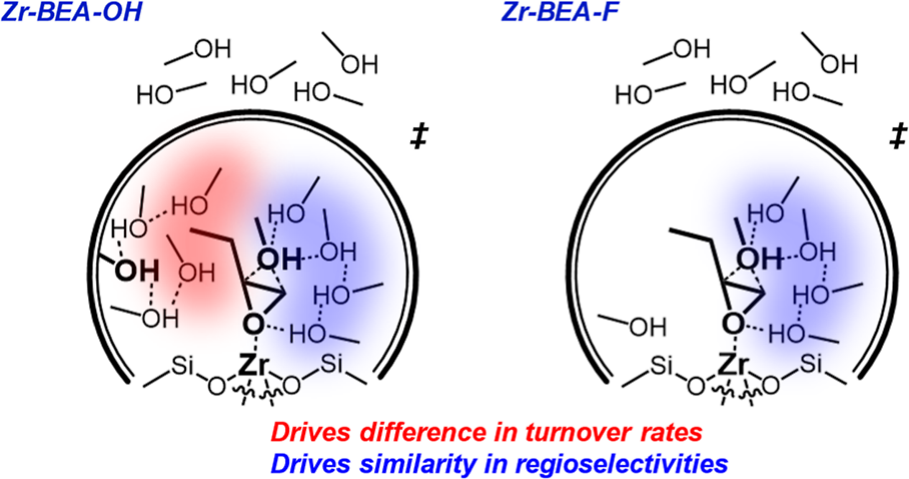
Proposed Origins of Differences between Zr-BEA-OH and Zr-BEA-F The disruption of hydrogen bonded-solvent by the alkyl tail of the transition state drives greater rates in Zr-BEA–OH, while the similar solvent structure surrounding the epoxide ring leads to similar regioselectivities among the materials.

The (SiOH)_*x*_ density of the M-BEA materials only slightly affects β values, in contrast to the strong effect of the active metal choice and solvent composition. The weak influence of (SiOH)_*x*_ on regioselectivity aligns with previous observations for 1,2-epoxyhexane ring-opening over Sn-BEA catalysts.^25^ While this trend corresponds with the weak dependence of rates, activation barriers, and intrapore solvent composition on the (SiOH)_*x*_ density of Al-BEA (vide supra), which differ from the persistent differences between the Zr-BEA-OH and Zr-BEA-F materials in previous sections. The explanation for this dissimilarity also remains unclear, but we postulate that the differences in solvent structure between Zr-BEA-OH and Zr-BEA-F do not influence β values as strongly as rates or activation barriers. The solvent environment surrounding the epoxide ring, where the nucleophilic attack occurs (i.e., near the active site), may sense the influence of (SiOH)_*x*_ groups less significantly than the extended solvent structure near the hydrophobic alkyl tail of the transition state (i.e., further from the active site) (see Scheme 4). In other words, the disruption of hydrogen-bonded solvent molecules by the alkyl tail of the transition state likely facilitates greater rates in Zr-BEA-OH over Zr-BEA-F, but does not contribute significantly to regioselectivity trends. To summarize, the observations from Figure 8 indicate that changing the active metal and solvent composition leads to differences in regioselectivities by altering the stability of transition states to form the ring-opening products, and the contributions from differences in (SiOH)_*x*_ density only weakly influence the regioselectivity for a given form of active site.

Industrial liquid-phase epoxide ring-opening processes commonly utilize homogeneous catalysts^31−33,86^ and ion-exchange resins,^87−89^ which provide high rates but suffer from issues with deactivation, regeneration, and separation. Microporous silicates (i.e., zeolites) offer a more stable and sustainable alternative that allows for precise control of the density and structure of intrapore liquids through changes in the active site character and (SiOH)_*x*_ density. While shown here for epoxide ring-opening, manipulating the properties of zeolite catalysts provides opportunities to control rates and selectivities of liquid-phase processes in general.

## 4 Conclusions

Rates and regioselectivities for epoxide ring-opening reactions depend intimately on the structure of solvent molecules, type of acid site (Lewis or Brønsted), and quantity of (SiOH)_*x*_ groups confined within zeolite pores. Introducing a high density of (SiOH)_*x*_ defects to the Lewis acidic zeolite (i.e., Zr-BEA-OH) facilitates C_4_H_8_O ring-opening rates by ∼10 times compared to the low density (SiOH)_*x*_ counterpart (i.e., Zr-BEA-F). Measurements of activation barriers, C_4_H_8_O adsorption enthalpies, and intrapore solvent compositions provide evidence that the disruption of networked solvent molecules within Zr-BEA-OH confers entropic gains to the transition state for ring-opening. This strong effect of (SiOH)_*x*_ nests on turnover rates over Lewis acidic zeolites corresponds to previous reports for alkene epoxidation^11,13,19−23^ and glucose isomerization,^10,14,16−18^ where (SiOH)_*x*_ groups stabilize hydrogen-bonded solvent structures that increase and decrease rates, respectively.

In contrast to the Lewis acids, Al-BEA-OH and Al-BEA-F show similar ring-opening rates (within a factor of 2), activation barriers, and solvent compositions across all conditions examined. These trends align with previous studies that Brønsted acid zeolites of different (SiOH)_*x*_ density provide rate constants for ethanol dehydration^65^ and rates for glucose acetalization^90^ within a factor of 2 in a condensed phase. Taking this work together with previous studies, the interactions between solvent molecules and (SiOH)_*x*_ nests in zeolites can strongly influence the kinetics of a variety of liquid phase catalytic reactions over Lewis acid sites but generally have a weak effect on kinetics over Brønsted acid sites. The H^+^ sites in the Brønsted acids likely lessen the effect of (SiOH)_*x*_ on the solvent environment near active sites, while the density of (SiOH)_*x*_ groups likely governs differences in solvent structure among the Zr-BEA materials. Interestingly, regioselectivities depend weakly on (SiOH)_*x*_ density for Al- and Zr-BEA, instead showing strong dependences on the acid type and reaction solvent composition. Therefore, different combinations of variables drive changes in rates and regioselectivities for ring-opening, providing opportunities to control these important reaction parameters independently. Evidently, the choices of zeolite acid type, intrapore (SiOH)_*x*_ density, and solvent carry strong consequences and warrant consideration during the design of catalysis and adsorption processes at zeolite–liquid interfaces.

## Supporting Information Available

X-ray diffraction, diffuse-reflectance UV–vis, ex situ Raman spectroscopy, transmission total-reflectance FT-infrared spectroscopy, ex situ solid-state ^27^Al NMR spectroscopy, scanning electron microscopy, Ar physisorption, GC sensitivity factors, hot filtrations, Madon–Boudart tests, 1,2-diphenyl-1,2-ethylene diamine active site titrations, supplemental epoxide ring-opening kinetics, rate expression derivation, excess enthalpies and free energies, supplemental activation barriers and equations, intrapore solvent uptakes, supplemental ITC thermograms, and supplemental adsorption enthalpies (PDF)

## References

[ref1] Kulprathipanja S. Zeolites in Industrial Separation and Catalysis; John Wiley & Sons, 2010; pp 173–303.

[ref2] Chen N.-Y. Shape Selective Catalysis in Industrial Applications; CRC Press, 2023.

[ref3] Noh G.; Shi Z.; Zones S. I.; Iglesia E. Isomerization and β-scission reactions of alkanes on bifunctional metal-acid catalysts: Consequences of confinement and diffusional constraints on reactivity and selectivity. J. Catal. 2018, 368, 389–410. 10.1016/j.jcat.2018.03.033.

[ref4] Sarazen M. L.; Iglesia E. Stability of bound species during alkene reactions on solid acids. Proc. Natl. Acad. Sci. U.S.A. 2017, 114, 201619557 10.1073/pnas.1619557114. PMC5441765 28461504

[ref5] Potts D. S.; Bregante D. T.; Adams J. S.; Torres C.; Flaherty D. W. Influence of solvent structure and hydrogen bonding on catalysis at solid-liquid interfaces. Chem. Soc. Rev. 2021, 50 (22), 12308–12337. 10.1039/D1CS00539A. 34569580

[ref6] Sievers C.; Noda Y.; Qi L.; Albuquerque E. M.; Rioux R. M.; Scott S. L. Phenomena Affecting Catalytic Reactions at Solid-Liquid Interfaces. ACS Catal. 2016, 6 (12), 8286–8307. 10.1021/acscatal.6b02532.

[ref7] Li G.; Wang B.; Resasco D. E. Solvent effects on catalytic reactions and related phenomena at liquid-solid interfaces. Surf. Sci. Rep. 2021, 76 (4), 100541 10.1016/j.surfrep.2021.100541.

[ref8] Bates J. S.; Gounder R. Kinetic effects of molecular clustering and solvation by extended networks in zeolite acid catalysis. Chem. Sci. 2021, 12 (13), 4699–4708. 10.1039/D1SC00151E. 34168752 PMC8179612

[ref9] Madon R. J.; Iglesia E. Catalytic reaction rates in thermodynamically non-ideal systems. J. Mol. Catal. A: Chem. 2000, 163 (1–2), 189–204. 10.1016/S1381-1169(00)00386-1.

[ref10] Vega-Vila J. C.; Gounder R. Quantification of Intraporous Hydrophilic Binding Sites in Lewis Acid Zeolites and Consequences for Sugar Isomerization Catalysis. ACS Catal. 2020, 10 (20), 12197–12211. 10.1021/acscatal.0c02918.

[ref11] Potts D. S.; Torres C.; Kwon O.; Flaherty D. W. Engineering intraporous solvent environments: effects of aqueous-organic solvent mixtures on competition between zeolite-catalyzed epoxidation and H_2_O_2_ decomposition pathways. Chem. Sci. 2023, 14 (12), 3160–3181. 10.1039/D2SC06473A. 36970093 PMC10034100

[ref12] Bregante D. T.; Potts D. S.; Kwon O.; Ayla E. Z.; Tan J. Z.; Flaherty D. W. Effects of Hydrofluoric Acid Concentration on the Density of Silanol Groups and Water Adsorption in Hydrothermally Synthesized Transition-Metal-Substituted Silicalite-1. Chem. Mater. 2020, 32 (17), 7425–7437. 10.1021/acs.chemmater.0c02405.

[ref13] Bregante D. T.; Johnson A. M.; Patel A. Y.; Ayla E. Z.; Cordon M. J.; Bukowski B. C.; Greeley J.; Gounder R.; Flaherty D. W. Cooperative Effects between Hydrophilic Pores and Solvents: Catalytic Consequences of Hydrogen Bonding on Alkene Epoxidation in Zeolites. J. Am. Chem. Soc. 2019, 141, 7302–7319. 10.1021/jacs.8b12861. 30649870

[ref14] Gounder R.; Davis M. E. Beyond shape selective catalysis with zeolites: Hydrophobic void spaces in zeolites enable catalysis in liquid water. AIChE J. 2013, 59 (9), 3349–3358. 10.1002/aic.14016.

[ref15] Zhang K.; Lively R. P.; Noel J. D.; Dose M. E.; McCool B. A.; Chance R. R.; Koros W. J. Adsorption of Water and Ethanol in MFI-Type Zeolites. Langmuir 2012, 28 (23), 8664–8673. 10.1021/la301122h. 22568830

[ref16] Cordon M. J.; Harris J. W.; Vega-Vila J. C.; Bates J. S.; Kaur S.; Gupta M.; Witzke M. E.; Wegener E. C.; Miller J. T.; Flaherty D. W.; Hibbitts D. D.; Gounder R. Dominant Role of Entropy in Stabilizing Sugar Isomerization Transition States within Hydrophobic Zeolite Pores. J. Am. Chem. Soc. 2018, 140 (43), 14244–14266. 10.1021/jacs.8b08336. 30265002

[ref17] Harris J. W.; Cordon M. J.; Di Iorio J. R.; Vega-Vila J. C.; Ribeiro F. H.; Gounder R. Titration and quantification of open and closed Lewis acid sites in Sn-Beta zeolites that catalyze glucose isomerization. J. Catal. 2016, 335, 141–154. 10.1016/j.jcat.2015.12.024.

[ref18] Cho H. J.; Gould N. S.; Vattipalli V.; Sabnis S.; Chaikittisilp W.; Okubo T.; Xu B.; Fan W. Fabrication of hierarchical Lewis acid Sn-BEA with tunable hydrophobicity for cellulosic sugar isomerization. Microporous Mesoporous Mater. 2019, 278, 387–396. 10.1016/j.micromeso.2018.12.046.

[ref19] Torres C.; Potts D. S.; Flaherty D. W. Solvent Mediated Interactions on Alkene Epoxidations in Ti-MFI: Effects of Solvent Identity and Silanol Density. ACS Catal. 2023, 13, 8925–8942. 10.1021/acscatal.3c01073.

[ref20] Tan J. Z.; Bregante D. T.; Torres C.; Flaherty D. W. Transition state stabilization depends on solvent identity, pore size, and hydrophilicity for epoxidations in zeolites. J. Catal. 2022, 405, 91–104. 10.1016/j.jcat.2021.11.029.

[ref21] Tang Z.; Yu Y.; Liu W.; Chen Z.; Wang R.; Liu H.; Wu H.; Liu Y.; He M. Deboronation-assisted construction of defective Ti(OSi)_3_OH species in MWW-type titanosilicate and their enhanced catalytic performance. Catal. Sci. Technol. 2020, 10 (9), 2905–2915. 10.1039/D0CY00126K.

[ref22] Bregante D. T.; Chan M. C.; Tan J. Z.; Ayla E. Z.; Nicholas C. P.; Shukla D.; Flaherty D. W. The shape of water in zeolites and its impact on epoxidation catalysis. Nat. Catal. 2021, 4 (9), 797–808. 10.1038/s41929-021-00672-4.

[ref23] Wang L.; Sun J.; Meng X.; Zhang W.; Zhang J.; Pan S.; Shen Z.; Xiao F.-S. A significant enhancement of catalytic activities in oxidation with H_2_O_2_ over the TS-1 zeolite by adjusting the catalyst wettability. Chem. Commun. 2014, 50 (16), 2012–2014. 10.1039/c3cc48220k. 24413395

[ref24] Izumi Y.; Onaka M. Chapter II. 1 Novel Catalytic Functions of Zeolites in Liquid-Phase Organic Reactions. In Studies in Surface Science and Catalysis; Elsevier, 1990; Vol. 54, pp 85–104. 10.1016/s0167-2991(08)60035-x.

[ref25] Spanos A. P.; Parulkar A.; Brunelli N. A. Enhancing hydrophobicity and catalytic activity of nano-Sn-Beta for alcohol ring opening of epoxides through post-synthetic treatment with fluoride. J. Catal. 2021, 404, 430–439. 10.1016/j.jcat.2021.10.023.

[ref26] Ford L.; Spanos A.; Brunelli N. A. Counting Sites in Lewis Acid Zeolite Sn-Beta: Connecting Site Quantification Experiments and Spectroscopy To Investigate the Catalytic Activity for the Alcohol Ring Opening of Epoxides. ACS Catal. 2023, 13 (17), 11422–11432. 10.1021/acscatal.3c02618.

[ref27] Potts D. S.; Komar J. K.; Locht H.; Flaherty D. W. Understanding Rates and Regioselectivities for Epoxide Methanolysis within Zeolites: Mechanism and Roles of Covalent and Non-covalent Interactions. ACS Catal. 2023, 13, 14928–14944. 10.1021/acscatal.3c04103.

[ref28] Hassan A.; Bagherzadeh E.; Anthony R. G.; Borsinger G.; Hassan A. Method of making alkylene glycols. U.S. Patent 8,354,562 B2, 2013.

[ref29] Chang C. D.; Hellring S. D. Hydrolysis of olefin oxides to glycols. U.S. Patent 4,620,044 A, 1986.

[ref30] Sedon J. H. Process for making glycol ethers using a heterogeneous catalyst. U.S. Patent 4,360,698 A, 1983.

[ref31] Teles J. H. Preparation of 1-methoxy-2-propanol. U.S. Patent 6,846,961 B2, 2005.

[ref32] Arrowood T. L.; Flick D. W.; Ackford J. F. Assembly for producing alkylene oxides and glycol ethers. U.S. Patent 9,822,087 B2, 2017.

[ref33] Goto S.; Itoh K.; Shinohara K.; Yoshii M.; Ishihara S. Method for producing glycols from oxirane compound. U.S. Patent 9,233,900 B2, 2016.

[ref34] Eckstein S.; Hintermeier P. H.; Zhao R.; Baráth E.; Shi H.; Liu Y.; Lercher J. A. Influence of Hydronium Ions in Zeolites on Sorption. Angew. Chem., Int. Ed. 2019, 58 (11), 3450–3455. 10.1002/anie.201812184. 30600885

[ref35] Ayla E. Z.; Potts D. S.; Bregante D. T.; Flaherty D. W. Alkene Epoxidations with H_2_O_2_ over Groups 4–6 Metal-Substituted BEA Zeolites: Reactive Intermediates, Reaction Pathways, and Linear Free-Energy Relationships. ACS Catal. 2021, 11 (1), 139–154. 10.1021/acscatal.0c03394.

[ref36] Blasco T.; Camblor M. A.; Corma A.; Esteve P.; Guil J. M.; Martinez A.; Perdigon-Melon J. A.; Valencia S. Direct Synthesis and Characterization of Hydrophobic Aluminum-Free Ti–Beta Zeolite. J. Phys. Chem. B 1998, 102, 75–88. 10.1021/jp973288w.

[ref37] Newsam J. M.; Treacy M. M. J.; Koetsier W. T.; Gruyter C. B. D. Structural characterization of zeolite beta. Proc. R. Soc. London, A 1988, 420, 375–405. 10.1098/rspa.1988.0131.

[ref38] Loewenstein W. The distribution of aluminum in the tetrahedra of silicates and aluminates. Am. Mineral. 1954, 39 (1–2), 92–96.

[ref39] Wang J.; Kispersky V. F.; Nicholas Delgass W.; Ribeiro F. H. Determination of the Au active site and surface active species via operando transmission FTIR and isotopic transient experiments on 2.3wt.% Au/TiO_2_ for the WGS reaction. J. Catal. 2012, 289, 171–178. 10.1016/j.jcat.2012.02.008.

[ref40] Jentys A.; Lercher J. Chapter 8 Techniques of zeolite characterization. In Studies in Surface Science and Catalysis; Elsevier, 2001; Vol. 137, pp 345–386. 10.1016/s0167-2991(01)80250-0.

[ref41] Zecchina A.; Bordiga S.; Spoto G.; Marchese L.; Petrini G.; Leofanti G.; Padovan M. Silicalite characterization. 2. IR spectroscopy of the interaction of carbon monoxide with internal and external hydroxyl groups. J. Phys. Chem. 1992, 96 (12), 4991–4997. 10.1021/j100191a048.

[ref42] Dzwigaj S.; Peltre M. J.; Massiani P.; Davidson A.; Che M.; Dzwigaj S.; Massiani P.; Sen T.; Sivasanker S. Incorporation of vanadium species in a dealuminated β zeolite. Chem. Commun. 1998, (1), 87–88. 10.1039/a704556e.

[ref43] Bregante D. T.; Flaherty D. W. Impact of Specific Interactions Among Reactive Surface Intermediates and Confined Water on Epoxidation Catalysis and Adsorption in Lewis Acid Zeolites. ACS Catal. 2019, 9 (12), 10951–10962. 10.1021/acscatal.9b03323.

[ref44] Rouquerol J.; Llewellyn P.; Rouquerol F. Is the BET equation applicable to microporous adsorbents?. In Studies in Surface Science and Catalysis; Elsevier: Amsterdam and Oxford, 2007; Vol. 160, pp 49–56. 10.1016/s0167-2991(07)80008-5.

[ref45] Galarneau A.; Villemot F.; Rodriguez J.; Fajula F.; Coasne B. Validity of the *t-plot* Method to Assess Microporosity in Hierarchical Micro/Mesoporous Materials. Langmuir 2014, 30 (44), 13266–13274. 10.1021/la5026679. 25232908

[ref46] Wang M.; Jaegers N. R.; Lee M. S.; Wan C.; Hu J. Z.; Shi H.; Mei D.; Burton S. D.; Camaioni D. M.; Gutierrez O. Y.; Glezakou V. A.; Rousseau R.; Wang Y.; Lercher J. A. Genesis and Stability of Hydronium Ions in Zeolite Channels. J. Am. Chem. Soc. 2019, 141 (8), 3444–3455. 10.1021/jacs.8b07969. 30698436

[ref47] Zhong P.; Zhang L.; Luo N.; Liu J. Advances in the Application of Acetonitrile in Organic Synthesis since 2018. Catalysts 2023, 13 (4), 761 10.3390/catal13040761.

[ref48] Kumagai N.; Matsunaga S.; Shibasaki M. Cooperative catalysis of a cationic ruthenium complex, amine base, and Na salt: Catalytic activation of acetonitrile as a nucleophile. J. Am. Chem. Soc. 2004, 126 (42), 13632–13633. 10.1021/ja0450509. 15493917

[ref49] Madon R. J.; Boudart M. Experimental Criterion for the Absence of Artifacts in the Measurement of Rates of Heterogeneous Catalytic Reactions. Ind. Eng. Chem. Fund. 1982, 21, 438–447. 10.1021/i100008a022.

[ref50] Flaherty D. W.; Bhan A. Improving the rigor and reproducibility of catalyst testing and evaluation in the laboratory. J. Catal. 2024, 431, 115408 10.1016/j.jcat.2024.115408.

[ref51] Chorkendorff I.; Niemantsverdriet J. W. H., Concepts of Modern Catalysis and Kinetics. 2nd ed.; Wiley-VCH Verlag Gmbh & Co.: Weinheim, 2007; pp 203–214.

[ref52] Shcherban N. D.; Filonenko S. M.; Barakov R. Y.; Sergiienko S. A.; Yu K.; Heinmaa I.; Ivaska A.; Murzin D. Y. New insights in evaluation of acid sites in micro-mesoporous zeolite-like materials using potentiometric titration method. Appl. Catal., A 2017, 543, 34–42. 10.1016/j.apcata.2017.05.039.

[ref53] Kester P. M.; Miller J. T.; Gounder R. Ammonia Titration Methods To Quantify Brønsted Acid Sites in Zeolites Substituted with Aluminum and Boron Heteroatoms. Ind. Eng. Chem. Res. 2018, 57 (19), 6673–6683. 10.1021/acs.iecr.8b00933.

[ref54] Moors S. L.; De Wispelaere K.; Van der Mynsbrugge J.; Waroquier M.; Van Speybroeck V. Molecular dynamics kinetic study on the zeolite-catalyzed benzene methylation in ZSM-5. ACS Catal. 2013, 3 (11), 2556–2567. 10.1021/cs400706e.

[ref55] Fifen J. J.; Nsangou M.; Dhaouadi Z.; Motapon O.; Jaidane N.-E. Solvation energies of the proton in methanol. J. Chem. Theory Comput. 2013, 9 (2), 1173–1181. 10.1021/ct300669v. 26588760

[ref56] Morrone J. A.; Tuckerman M. E. *Ab initio* molecular dynamics study of proton mobility in liquid methanol. J. Chem. Phys. 2002, 117 (9), 4403–4413. 10.1063/1.1496457.

[ref57] Potts D. S.; Jeyaraj V. S.; Kwon O.; Ghosh R.; Mironenko A. V.; Flaherty D. W. Effect of Interactions between Alkyl Chains and Solvent Structures on Lewis Acid Catalyzed Epoxidations. ACS Catal. 2022, 12 (21), 13372–13393. 10.1021/acscatal.2c03493.

[ref58] Kwon O.; Ayla E. Z.; Potts D. S.; Flaherty D. W. Effects of Solvent-Pore Interaction on Rates and Barriers for Vapor Phase Alkene Epoxidation with Gaseous H_2_O_2_ in Ti-BEA Catalysts. ACS Catal. 2023, 13, 6430–6444. 10.1021/acscatal.3c00730.

[ref59] Di Iorio J. R.; Johnson B. A.; Román-Leshkov Y. Ordered Hydrogen-Bonded Alcohol Networks Confined in Lewis Acid Zeolites Accelerate Transfer Hydrogenation Turnover Rates. J. Am. Chem. Soc. 2020, 142 (45), 19379–19392. 10.1021/jacs.0c09825. 33108165

[ref60] Qi L.; Alamillo R.; Elliott W. A.; Andersen A.; Hoyt D. W.; Walter E. D.; Han K. S.; Washton N. M.; Rioux R. M.; Dumesic J. A.; Scott S. L. Operando Solid-State NMR Observation of Solvent-Mediated Adsorption-Reaction of Carbohydrates in Zeolites. ACS Catal. 2017, 7, 3489–3500. 10.1021/acscatal.7b01045.

[ref61] Dejaco R. F.; Bai P.; Tsapatsis M.; Siepmann J. I. Adsorptive Separation of 1-Butanol from Aqueous Solutions Using MFI- and FER-Type Zeolite Frameworks: A Monte Carlo Study. Langmuir 2016, 32 (8), 2093–2101. 10.1021/acs.langmuir.5b04483. 26818393

[ref62] Bai P.; Tsapatsis M.; Siepmann J. I. Multicomponent Adsorption of Alcohols onto Silicalite-1 from Aqueous Solution: Isotherms, Structural Analysis, and Assessment of Ideal Adsorbed Solution Theory. Langmuir 2012, 28 (44), 15566–15576. 10.1021/la303247c. 23050981

[ref63] Dejaco R. F.; Dorneles De Mello M.; Nguyen H. G. T.; Jeon M. Y.; van Zee R. D.; Tsapatsis M.; Siepmann J. I. Vapor- and liquid-phase adsorption of alcohol and water in silicalite-1 synthesized in fluoride media. AIChE J. 2020, 66 (4), e16868 10.1002/aic.16868. PMC7712499 33281192

[ref64] Pahari S.; Dorneles De Mello M.; Shah M. S.; Josephson T. R.; Ren L.; Nguyen H. G. T.; Van Zee R. D.; Tsapatsis M.; Siepmann J. I. Ethanol and Water Adsorption in Conventional and Hierarchical All-Silica MFI Zeolites. ACS Phys. Chem. Au 2022, 2 (2), 79–88. 10.1021/acsphyschemau.1c00026. 36855513 PMC9718309

[ref65] Bates J. S.; Bukowski B. C.; Greeley J.; Gounder R. Structure and solvation of confined water and water–ethanol clusters within microporous Brønsted acids and their effects on ethanol dehydration catalysis. Chem. Sci. 2020, 11 (27), 7102–7122. 10.1039/D0SC02589E. 33250979 PMC7690318

[ref66] Oumi Y.; Miyajima A.; Miyamoto J.; Sano T. Binary mixture adsorption of water and ethanol on silicalite. In Studies in Surface Science and Catalysis; Elsevier, 2002; Vol. 142, pp 1595–1602. 10.1016/s0167-2991(02)80329-9.

[ref67] Ahn H.; Lee H.; Lee S.-B.; Lee Y. Pervaporation of an aqueous ethanol solution through hydrophilic zeolite membranes. Desalination 2006, 193 (1–3), 244–251. 10.1016/j.desal.2005.06.062.

[ref68] Sano T.; Ejiri S.; Yamada K.; Kawakami Y.; Yanagishita H. Separation of acetic acid-water mixtures by pervaporation through silicalite membrane. J. Membr. Sci. 1997, 123 (2), 225–233. 10.1016/S0376-7388(96)00224-4.

[ref69] Bell R. P. The theory of reactions involving proton transfers. Proc. R. Soc. London, A 1936, 154 (882), 414–429. 10.1098/rspa.1936.0060.

[ref70] Evans M.; Polanyi M. Further considerations on the thermodynamics of chemical equilibria and reaction rates. Trans. Faraday Soc. 1936, 32, 1333–1360. 10.1039/tf9363201333.

[ref71] Brønsted J.; Pedersen K. Stöchiometrie und verwandtschaftslehre. Z. Phys. Chem. 1924, 108, 185–235. 10.1515/zpch-1924-10814.

[ref72] Hammond G. S. A Correlation of Reaction Rates. J. Am. Chem. Soc. 1955, 77 (2), 334–338. 10.1021/ja01607a027.

[ref73] Anslyn E. V.; Dougherty D. A. Modern Physical Organic Chemistry; University Science, 2005; p 358.

[ref74] Cucciniello R.; Ricciardi M.; Vitiello R.; Di Serio M.; Proto A.; Capacchione C. Synthesis of monoalkyl glyceryl ethers by ring opening of glycidol with alcohols in the presence of lewis acids. ChemSusChem 2016, 9 (23), 3272–3275. 10.1002/cssc.201600989. 27880034

[ref75] Deshpande N.; Parulkar A.; Joshi R.; Diep B.; Kulkarni A.; Brunelli N. A. Epoxide ring opening with alcohols using heterogeneous Lewis acid catalysts: Regioselectivity and mechanism. J. Catal. 2019, 370, 46–54. 10.1016/j.jcat.2018.11.038.

[ref76] Mirkhani V.; Tangestaninejad S.; Yadollahi B.; Alipanah L. Efficient regio-and stereoselective ring opening of epoxides with alcohols, acetic acid and water catalyzed by ammonium decatungstocerate (IV). Tetrahedron 2003, 59 (41), 8213–8218. 10.1016/j.tet.2003.08.018.

[ref77] Das S.; Asefa T. Epoxide Ring-Opening Reactions with Mesoporous Silica-Supported Fe(III) Catalysts. ACS Catal. 2011, 1 (5), 502–510. 10.1021/cs1001256.

[ref78] Bhagat M. N.; Bennett C. K.; Chang G.-F.; Zhu Y.; Raghuraman A.; Belowich M. E.; Nguyen S. T.; Broadbelt L. J.; Notestein J. M. Enhancing the Regioselectivity of B(C_6_F_5_)_3_-Catalyzed Epoxide Alcoholysis Reactions Using Hydrogen-Bond Acceptors. ACS Catal. 2019, 9 (10), 9663–9670. 10.1021/acscatal.9b03089.

[ref79] Hansen T.; Vermeeren P.; Haim A.; van Dorp M. J.; Codée J. D. C.; Bickelhaupt F. M.; Hamlin T. A. Regioselectivity of Epoxide Ring-Openings via S_N_2 Reactions Under Basic and Acidic Conditions. Eur. J. Org Chem. 2020, 2020 (25), 3822–3828. 10.1002/ejoc.202000590.

[ref80] Parker R.-E.; Isaacs N. Mechanisms of epoxide reactions. Chem. Rev. 1959, 59 (4), 737–799. 10.1021/cr50028a006.

[ref81] Klein D. R. Organic Chemistry; John Wiley & Sons, 2022.

[ref82] McMurry J. Organic Chemistry. 9th ed.; Cengage Learning Boston, MA, USA: Boston, MA, USA, 2016.

[ref83] Ogawa H.; Miyamoto Y.; Fujigaki T.; Chihara T. Ring-opening of 1,2-epoxyalkane with alcohols over H-ZSM-5 in liquid phase. Catal. Lett. 1996, 40 (3–4), 253–255. 10.1007/BF00815291.

[ref84] Terblans Y. M.; Huyser M.; Young D. A.; Green M. J. The synthesis of butene glycol ethers with aluminium triflate. Can. J. Chem. 2006, 84 (6), 859–866. 10.1139/v06-086.

[ref85] Williams D. B. G.; Lawton M. Aluminium triflate: a remarkable Lewis acid catalyst for the ring opening of epoxides by alcohols. Org. Biomol. Chem. 2005, 3 (18), 3269–3272. 10.1039/b508924g. 16132088

[ref86] Li X.; Candela L. M.; Leipa M. A.; Leyshon D. W. Production of propylene glycol monoalkyl ether. EP 3362427 B1, 2021.

[ref87] Chan C. C.; Yamada E.; Billig B. J.; McGovern S. Process for preparing ethylene glycol. U.S. Patent 10,807,929 B2, 2020.

[ref88] Van Kruchten E. M. G. A. Process for the preparation of alkylene glycols. U.S. Patent 7,435,858 B2, 2008.

[ref89] Strickler G. R.; Landon V. G.; Lee G.-S. J. Process for the preparation of alkylene glycols. U.S. Patent 6,211,419 B1, 2001.

[ref90] Camblor M.; Corma A.; Iborra S.; Miquel S.; Primo J.; Valencia S. Beta zeolite as a catalyst for the preparation of alkyl glucoside surfactants: the role of crystal size and hydrophobicity. J. Catal. 1997, 172 (1), 76–84. 10.1006/jcat.1997.1837.

