# Supplementary material for: Consequences of Pore Polarity and Solvent Structure on Epoxide Ring-Opening in Lewis and Brønsted Acid Zeolites
Source: JACS Au. 2024 Jul 9;4(9):3501–18. doi: 10.1021/jacsau.4c00398 (PMC11423312; doi:10.1021/jacsau.4c00398)
Supplement: Supplementary file 1 — au4c00398_si_001.pdf [file au4c00398_si_001.pdf]

Supporting Information

Consequences of Pore Polarity and Solvent Structure on Epoxide Ring-Opening in Lewis and Brønsted Acid Zeolites

David S. Potts,[1] Jessica K. Komar,[1] Matthew A. Jacobson, [1,2] Huston Locht,[1,2] David W. Flaherty[1,2*]

[1]Department of Chemical and Biomolecular Engineering
University of Illinois Urbana-Champaign, Urbana, Illinois 61801 (USA)

[2]School of Chemical and Biomolecular Engineering
Georgia Institute of Technology, Atlanta, Georgia 30332 (USA)

*Corresponding author e-mail: dflaherty3@gatech.edu

S1. Catalyst Characterization

S1.1 X-Ray Diffraction to Observe Crystallinity of M-BEA Catalysts

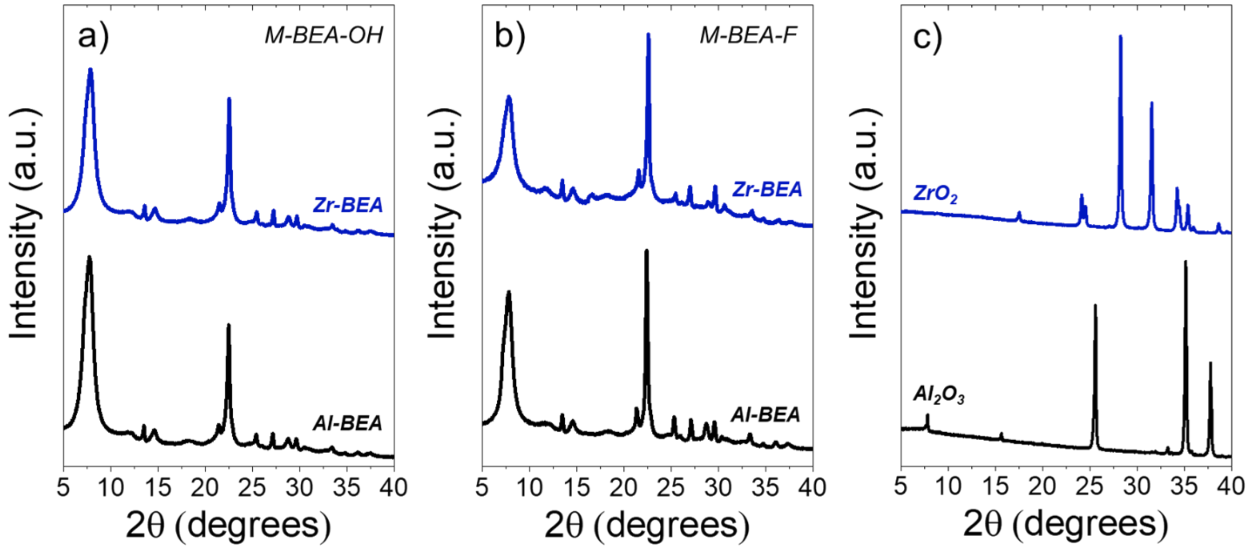

Figure S1. Powder X-ray diffractograms obtained with Cu Kα radiation (0.15418 nm) under ambient conditions for a) M-BEA-OH, b) M-BEA-F (Al (black), Zr (blue)), and c) the corresponding bulk metal oxides (γ-Al_2O_3 (black), ZrO_2 (blue)). Spectra are vertically offset for clarity.

The crystallographic features for each M-BEA are representative of the *BEA framework.[1] As displayed in Figures S1a and S1b, each M-BEA material shows weak features around 25.5, 27, 29, and 33 degrees. The sharper feature at ~22.5 degrees for the M-BEA-F materials in Figure S1b indicates a highly crystalline material, consistent with previous reports for zeolites synthesized in fluoride media.[2] The broader feature for the M-BEA-OH in Figure S1a suggests a smaller crystallite size[3] and the presence of internal $(SiOH)_x$ defects formed by dealumination of the parent Al-BEA material (Si:Al = 20).

Figure S1c reveals that each of these peak locations coincides with strong features in the diffractograms of at least one of the metal oxides. However, the presence of the weak features in all M-BEA suggests that the features are characteristic of the *BEA framework, rather than originating from the presence of metal oxide. Overall, the XRD patterns in Figure S1 support that each M-BEA possesses the crystalline *BEA framework, with minimal to no detectable metal oxide crystallites present.

S1.2 Diffuse Reflectance UV-Vis Spectra to Infer Metal Atom Dispersity

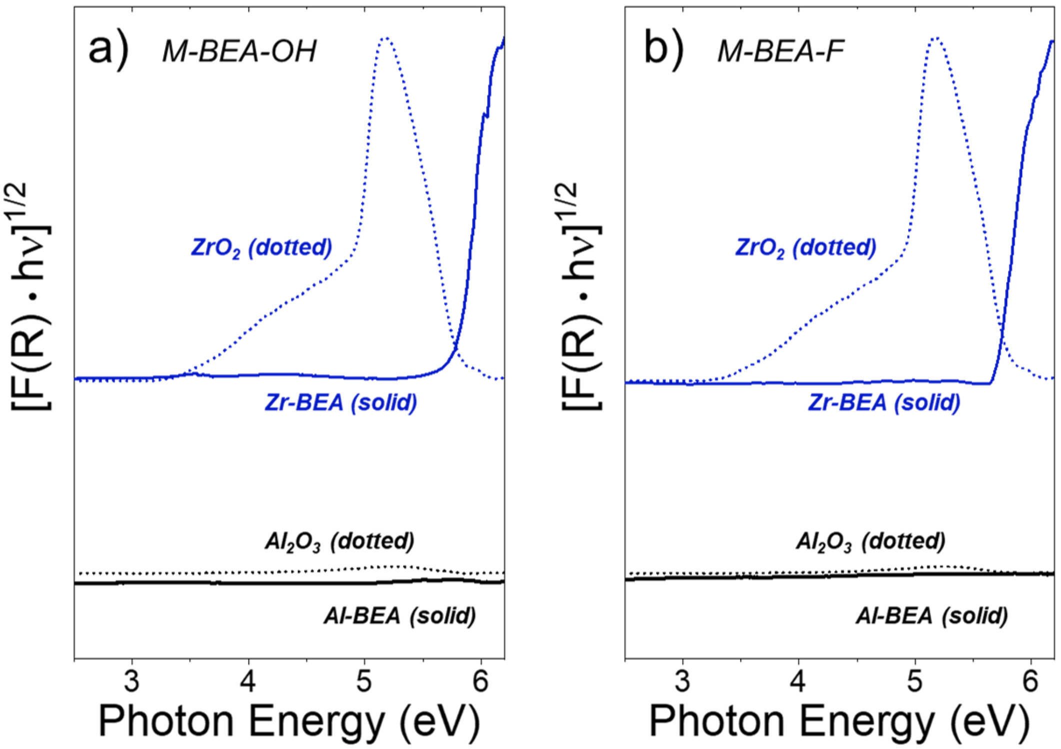

Figure S2. Tauc plots obtained with a UV-Vis spectrophotometer at ambient conditions for a) M-BEA-OH and b) M-BEA-F (solid lines; Al (−), Zr (−)) and the respective metal oxides (dotted lines; γ-Al_2O_3 (--), ZrO_2 (--)). UV-Vis spectra were obtained from 200 to 800 nm. The Tauc plots were normalized to the most intense features and are vertically offset for clarity.

The band gaps for each M-BEA and the respective metal oxides were obtained by extrapolating the linear absorbance region of the Tauc plot in Figure S2 to the baseline. The intersection of the baseline and linear region represents the band gap photon energy. Each Al-BEA and γ-Al_2O_3 show negligible features within the region of photon energies examined, consistent with reported band gaps greater than 7 eV for γ-Al_2O_3.[4-5] Table S1 reveals that the band gaps for each Al- and Zr-BEA are greater than the band gaps for the metal oxides, providing evidence that the metal atoms are well dispersed in the *BEA framework.

Catalyst	M-BEA<br>Band Gap (eV)	Metal Oxide<br>Band Gap (eV)
Al-BEA-F	--	--
Al-BEA-OH	--	--
Zr-BEA-F	5.7	4.9
Zr-BEA-OH	5.8	4.9

Table S1. Calculated band gaps for M-BEA and metal oxide materials. The band gaps for the Al materials likely exceeds the regime of wavelengths possible to measure on the DRUV-Vis spectrophotometer.

The photon energy *E* in electron volts (eV) was obtained from the wavelength as follows:

$$E = \frac{3 * 10^8 \frac{m}{s}}{Wavelength\ (nm) * \frac{1\ m}{10^9\ nm}} * h * \left(6.242 * 10^{18} \frac{eV}{J}\right)$$

where *h* equals the Planck constant. The ordinate from Figure S2 was obtained from the ordinate of the raw spectra, the percent reflectance (% R). The Kubelka-Munk function (*F(R))* was calculated from % R:

$$F(R) = \frac{\left(1 - \frac{\%R}{100}\right)^2}{2 * \left(\frac{\%R}{100}\right)}$$

F(R) was then multiplied by the photon energy, and taking the square root of the resulting quantity yielded the ordinate for Figure S2.

S1.3 *Ex situ* Raman Spectra of M-BEA Materials to Confirm Metal Coordination

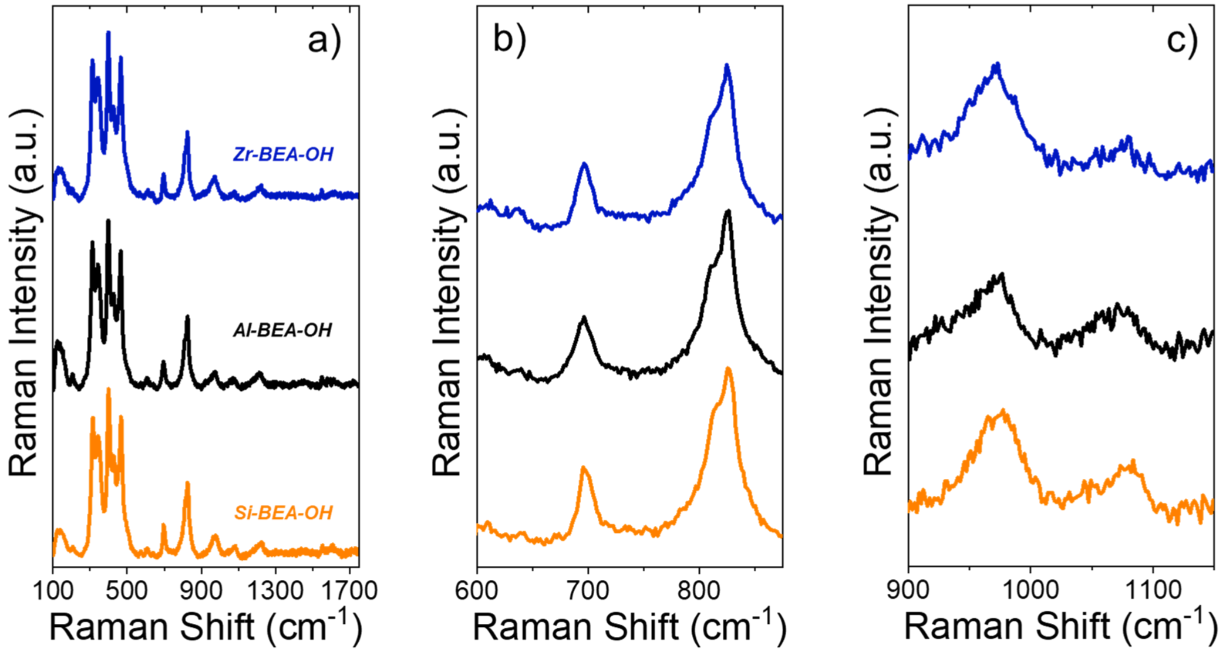

Figure S3. *Ex situ* Raman spectra of M-BEA-OH (Zr (blue), Al (black), Si (orange)) at (a) the full range of Raman shifts examined, (b) magnified view of the range of 600-875 cm^{-1}, and (c) magnified view of the 900-1150 cm^{-1} region. The spectra are an average of 10 scans with an exposure time of 20 s. Spectra are normalized by the maximum feature at ~400 cm^{-1} and are vertically offset for clarity. The spectra were taken at ambient conditions with a Raman spectrometer (Renishaw, InVia) equipped with a 532 nm laser that delivered a power density of ~2 mW μm^{-2}.

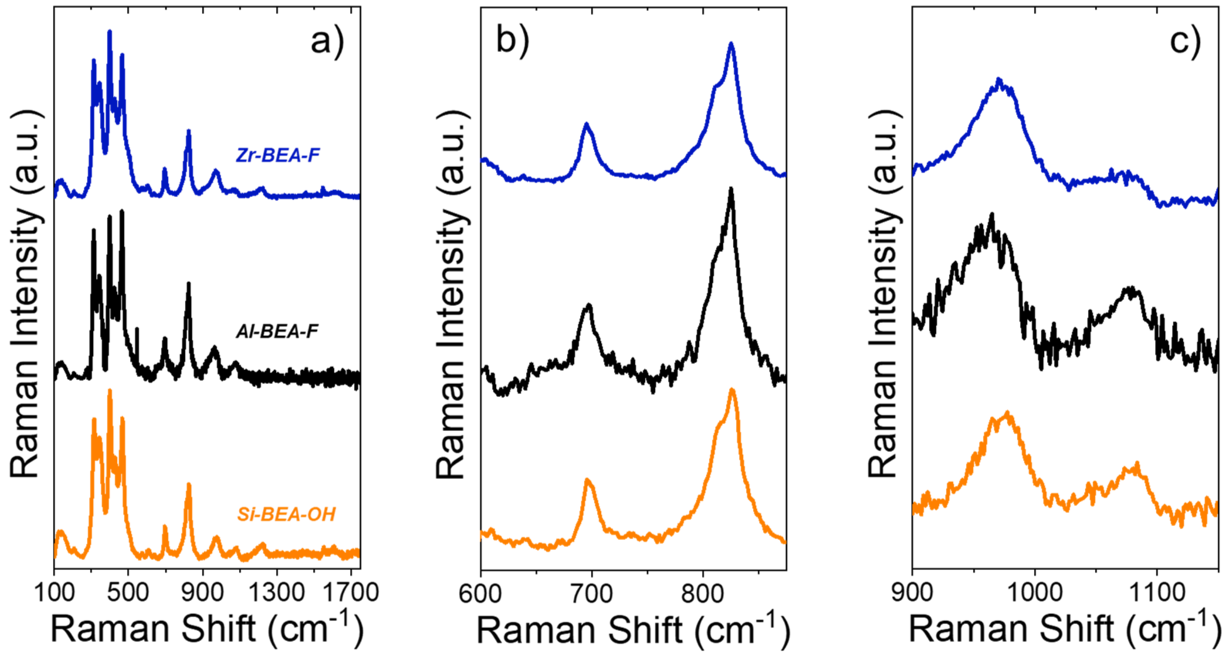

Figure S4. *Ex situ* Raman spectra of M-BEA-F (Zr (blue), Al (black)) with Si-BEA-OH for reference (orange).

Figures S3a and S4a reveal that each M-BEA and a dealuminated *BEA material (Si-BEA-OH) possess similar Raman spectra, with features characteristic of the *BEA framework. The intense peaks between 250 and 550 cm^{-1} represent features of the *BEA framework: the peaks at 315 and 345 cm^{-1} are assigned to six-membered rings, the peaks at 400 and 425 cm^{-1} are assigned to five-membered rings, and the peak at 465 cm^{-1} represents four-membered rings characteristic of the *BEA framework.[6-7] The peak at ~695 cm^{-1} has previously been reported on Raman spectra of crystalline *BEA zeolites, suggesting the feature may be characteristic of the *BEA framework.[8-9] The peak at ~820 cm^{-1} represents a symmetric skeletal mode of microporous silicates.[10-11]

All M-BEA materials show a feature at 144 cm^{-1}. The feature is also present in Si-BEA, which supports that the feature results from a mode inherent to the *BEA framework. Alternatively, the feature may simply be an artifact of the Raman filter used to suppress signal from Rayleigh scattering. Figures S3b and S4b zooms into a region in which Al_2O_3 (640, 751 cm^{-1})[12-14] and ZrO_2 (610, 640 cm^{-1})[15-17] have been reported to show Raman features. All M-BEA materials show nearly identical spectra in this region, with no notable features in the regions where the respective metal oxides contain features. These data support that all M-BEA materials do not contain oligomeric metal oxide structures.

Figure S3c and S4c show that each M-BEA possesses features at ~960 and ~1080 cm^{-1}. The feature at ~960 cm^{-1} has been commonly attributed to silanol defects.[18-20] The peak at ~1080 cm^{-1} plausibly originates from the *BEA framework. Ti-O-Si features have been commonly reported in the literature at ~960 and ~1125 cm^{-1}, respectively.[21-24] However, similar features have not been well reported for Al- or Zr-containing zeolites. Therefore, the presence of each metal in the framework cannot be confirmed with Raman spectra but is indirectly supported by the absence of metal oxide features.

The Raman spectra in Figures S3 and S4 suggest that the Al and Zr atoms within M-BEA predominately reside at tetrahedral framework positions within the *BEA zeolite framework.

S1.4 Infrared Spectra of M-BEA Materials to Measure Silanol Density

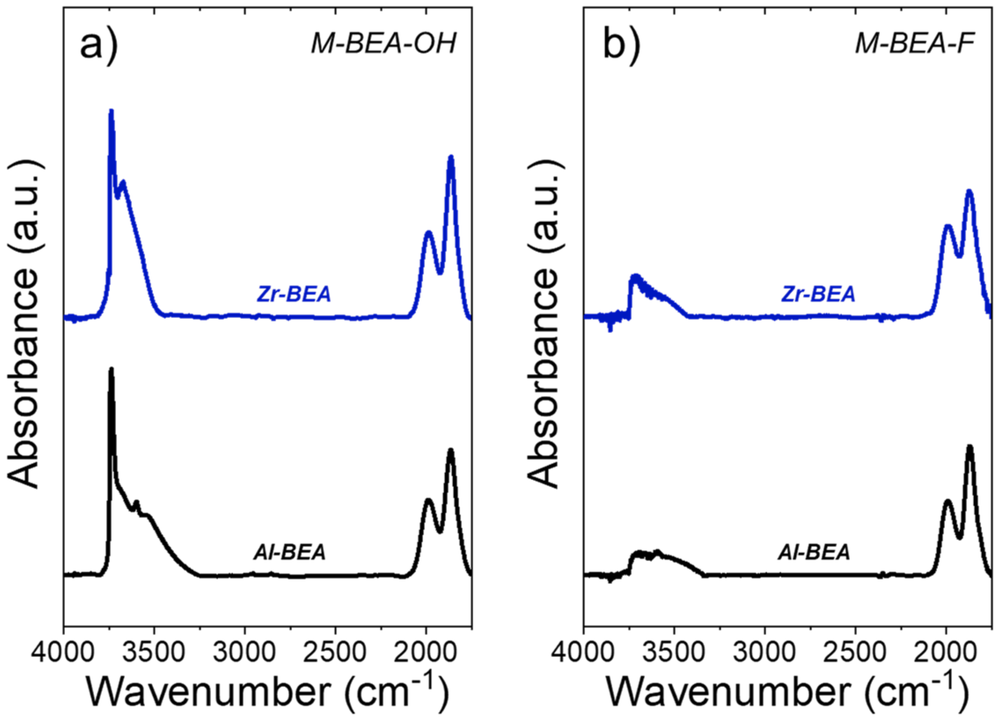

Figure S5. Infrared spectra of dehydrated samples of a) M-BEA-OH, b) M-BEA-F (Zr (blue), Al (black)). The samples were dehydrated under flowing He (101 kPa He, 50 cm^3 min^{-1}) at 573 K prior to measurement. Spectra are vertically offset for clarity.

The greater *v*(O-H) areas (3300-3750 cm^{-1}) for each M-BEA-OH compared to M-BEA-F, with respect to the *v*(Si-O-Si) area (1800-2100 cm^{-1}), shows that the M-BEA-OH materials possess a greater density of $(SiOH)_x$ groups than the M-BEA-F materials. The Φ_{IR} values in Table 1 of the main text were calculated by peak fitting on Origin 2021 (OriginLab Corporation). The peak areas for *v*(O-H) and *v*(Si-O-Si) were calculated with Gaussian fits (example shown in Figure S6).

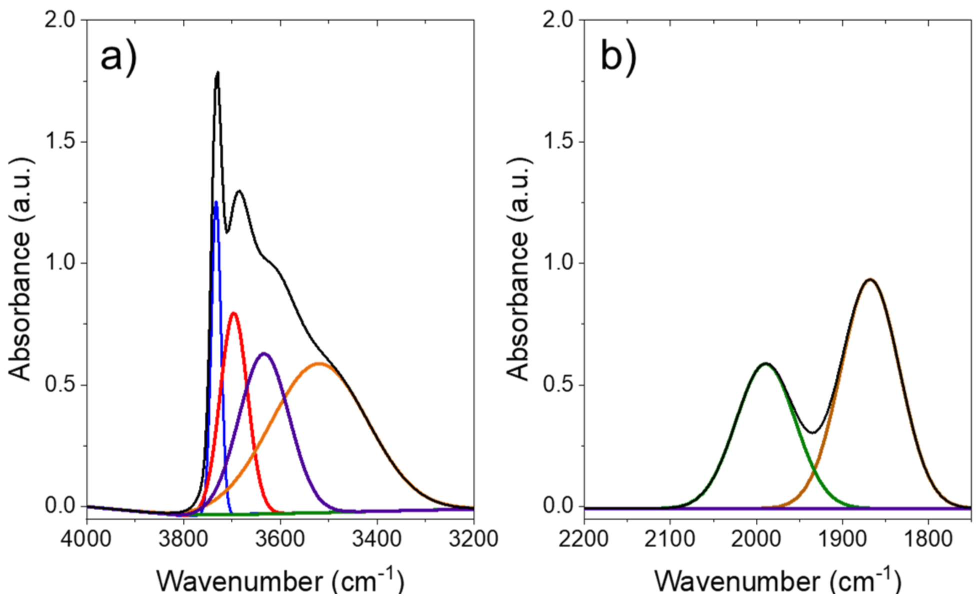

Figure S6. Fitted peaks for the (a) networked (red, purple, orange) and isolated (blue) Si-OH region and (b) Si-O-Si overtones at 1865 (brown) and 2000 cm^{-1} (green) for Zr-BEA-OH.

As shown in Equation 1 in the main text, the equation to calculate Φ_{IR} is:

$$\Phi_{IR} = \frac{A_{\nu(O-H)}}{A_{\nu(Si-O-Si)}} \quad \text{(S1)}$$

The red, purple, and orange peaks represent networked SiOH features which are summed to determine $A_{v(O-H)}$. The green and brown peaks in the Si-O-Si region were summed together to get $A_{\nu(Si-O-Si)}$. The blue peak in *v*(O-H) represent isolated SiOH features, so these peak areas were excluded from the Φ_{IR} calculations.

S1.5 Calculation of $(SiOH)_x$ (unit cell)$^{-1}$ in M-BEA-OH

The density of $(SiOH)_x$ per unit cell in M-BEA-OH was estimated based on the initial quantity of Al present in the commercial zeolite (Si:Al = 20). A *BEA unit cell contains 64 T atoms (i.e., Si, Al, or Zr), so the initial quantity of Al per unit cell equals 3.047:

$$Si + Al = 64$$

$$20Al + Al = 64$$

$$\boldsymbol{Al = 3.047\ \ per\ unit\ cell}$$

Assuming all Al atoms are removed in the thorough dealumination treatment for the Lewis acids, the dealumination produces 3.047 $(SiOH)_x$ (unit cell)$^{-1}$. The metal weight loading allows for the calculation of the Si:M ratio for each M-BEA:

$$Si + M = 64$$

$$(Si{:}M)M + M = 64$$

This can be used to calculate the number of metal atoms per unit cell incorporated into the formed $(SiOH)_x$. The final $(SiOH)_x$ (unit cell)$^{-1}$ for Zr-BEA-PJ is equal to the initial amount of Al (assumed that all Al is removed) subtracted by the added Zr. In contrast, the $(SiOH)_x$ (unit cell)$^{-1}$ for the Brønsted acid equals the initial quantity of Al subtracted by the amount of Al removed:

$$(SiOH)_{x,Zr-BEA-OH} = 3.047\ - added\ Zr$$

$$(SiOH)_{x,Zr-BEA-OH} = 3.047\ - removed\ Al$$

Table S2 reports metal loadings per unit cell from ICP and calculated $(SiOH)_x$ per unit cell. The calculated values predict a wider range of zeolite hydrophilicity than the Φ_{IR} calculations in Section S1.4 and Table 1. However, rate measurements as a function of metal loading (Section S4, *vide infra*) demonstrate a weak rate dependence on metal loading, and therefore $(SiOH)_x$ (unit cell)$^{-1}$, within the range of loadings examined for the M-BEA-OH materials here.

Catalyst	M (unit cell)$^{-1}$	$(SiOH)_x$ (unit cell)$^{-1}$
Al-BEA-OH	0.94	2.11
Zr-BEA-OH	0.45	2.59

Table S2. Metal loadings and $(SiOH)_x$ per unit cell, determined from metal loadings from ICP and estimated Al removed by dealumination treatments.

S1.6 Infrared Spectra of C_5H_5N and CD_3CN Adsorption to Probe Active Site Character

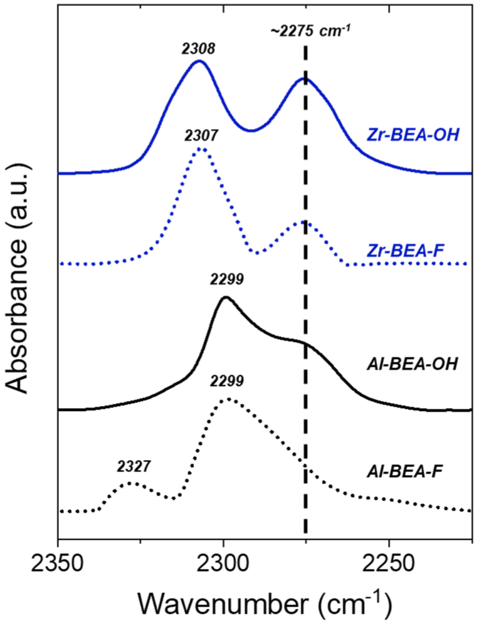

Figure S7. Infrared spectra of deuterated acetonitrile (CD_3CN) bound to hydrophilic (solid lines) and hydrophobic (dashed lines) Al-BEA (black) and Zr-BEA (blue) materials (0.03 kPa CD_3CN, 101 kPa Ar, 303 K). All spectra are normalized to the area of the Si-O-Si region of the zeolite from 1750-2100 cm^{-1}. Spectra are vertically offset for clarity.

Zr-BEA-OH and Zr-BEA-F possess single peaks at ~2308 cm^{-1}, in agreement with previous assignments of Lewis acid-bound CD_3CN within Zr-incorporated[25-26] zeolites. Both Zr-BEA materials also show an absorbance feature at ~2275 cm^{-1}, which has been reported as CD_3CN coordinated to Si-OH groups within *BEA.[27-28] Zr-BEA-F shows a less intense feature at ~2275 cm^{-1}, aligning with the lower density of $(SiOH)_x$ groups within Zr-BEA-F compared to Zr-BEA-OH. While each Zr-BEA only shows a single Lewis acid feature in Figure S7, each material likely contains both open and closed sites. Previous work demonstrated the presence of two Lewis acid features over Zr-incorporated zeolites from the infrared spectra of CO adsorption under cryogenic conditions, which the authors attributed to the presence of both open and closed Zr sites.[25] We do not rule out that -OH functions at open Zr sites could also contribute to the feature at ~2275 cm^{-1}. Furthermore, several publications also argue that closed Lewis acid sites may open in the presence of protic molecules such as H_2O[29-31] and alcohols,[32-33] meaning that Lewis acid sites in M-BEA could open *in situ* in the presence of CH_3OH and convolute *ex situ* comparisons of site distributions.

The feature at ~2299 cm^{-1} for Al-BEA-OH and Al-BEA-F is consistent with previous assignments for Brønsted acidic Al sites.[28, 34] Al-BEA-OH shows a shoulder at ~2275 cm^{-1} that likely originates from $(SiOH)_x$ nests in the hydrophilic material. The absence of a clear shoulder at ~2275 cm^{-1} in Al-BEA-F agrees with the lower $(SiOH)_x$ density of the material relative to Al-BEA-OH. Notably, Al-BEA-F also shows a feature at ~2327 cm^{-1} that is not present in Al-BEA-OH. Extraframework Lewis acidic Al species have been reported to show a feature in this range,[28, 34] suggesting that not all Al exists as framework Brønsted acid sites within Al-BEA-F. ^{27}Al NMR spectra and active site titration experiments (Figures S9, S10, and Figure S18, *vide infra*) suggest that ~ 4 and 10% of Al sites exist as extraframework species in Al-BEA-OH and Al-BEA-F, respectively. Nevertheless, the Raman spectra for Al-BEA-OH and Al-BEA-F do not show noticeable extraframework Al features. Al-BEA-OH and Al-BEA-F may both contain a minor fraction of extraframework Lewis acid Al sites, but the Al atoms predominantly exist as Brønsted

acid sites. Collectively, the spectra of adsorbed CD_3CN in Figure S7 demonstrate the presence of Lewis and Brønsted acid sites within the M-BEA materials.

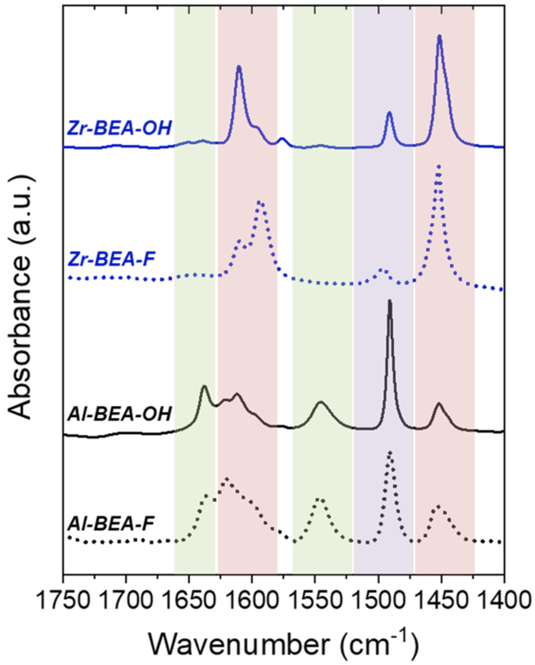

Figure S8. Infrared spectra of pyridine (C_5H_5N) bound to Lewis and Brønsted acid sites over hydrophilic (solid lines) and hydrophobic (dashed lines) Al-BEA (black) and Zr-BEA (blue) materials (1.4 kPa C_5H_5N, 101 kPa Ar, 393 K). All spectra are normalized to the area of the Si-O-Si region of the zeolite from 1750-2100 cm^{-1}. The highlighted regions on the plot represent regions for C_5H_5N absorbance features coordinated to only Brønsted acid sites (green), only Lewis acid sites (red), and either Lewis or Brønsted acid sites (purple). Spectra are vertically offset for clarity.

Each material possesses a feature at ~1450 cm^{-1} that has been attributed to C_5H_5N bound to only Lewis acid sites.[35-38] All M-BEA materials also show features at ~1490 cm^{-1}, corresponding to C_5H_5N bound to Lewis or Brønsted acid sites.[35, 39] Each material also shows peaks in the region of 1580-1625 cm^{-1}, commonly attributed to Lewis acid-bound C_5H_5N.[40-42] The presence of these features in the Al-BEA zeolites may signal the presence of some fraction of extraframework Lewis acidic Al species. This aligns with the presence of the Lewis acid Al feature on the CD_3CN spectra of Al-BEA-F, but contrasts with the absence of any Lewis acid feature for CD_3CN adsorption over Al-BEA-OH. Both Al-BEA-OH and Al-BEA-F also show features at ~1550 and ~1640 cm^{-1}, which solely originate from protonated C_5H_5N at Brønsted acid sites.[35-39] Zr-BEA-OH and Zr-BEA-F do not contain features in this region, suggesting that all Zr sites in these materials are Lewis acidic. Each material also shows a feature at ~1575 cm^{-1} of varying intensity, which has been attributed to physisorbed C_5H_5N.[35, 38, 43]

Notably, the Zr-BEA materials show distinct features at ~1595 and ~1610 cm^{-1}. The peak at ~1610 cm^{-1} has previously been assigned to C_5H_5N coordinated to Lewis acidic Zr sites.[44-45] However, the peak at ~1595 cm^{-1} resides in a position previously assigned to C_5H_5N hydrogen-bonded to -OH groups.[37-39, 41] This peak may arise from C_5H_5N coordinated to the -OH group from Zr-OH or the adjacent Si-OH in open sites within Zr-BEA. However, a feature at ~1595 cm^{-1} has also been attributed to gas-phase C_5H_5N, which convolutes the assignment of that feature the Zr-BEA materials. Overall, the spectra of adsorbed C_5H_5N in Figure S8 provide insight into the distribution of active sites within each M-BEA.

S1.7 Identifying Al Structure within Al-BEA Materials Using ^{27}Al NMR Spectra

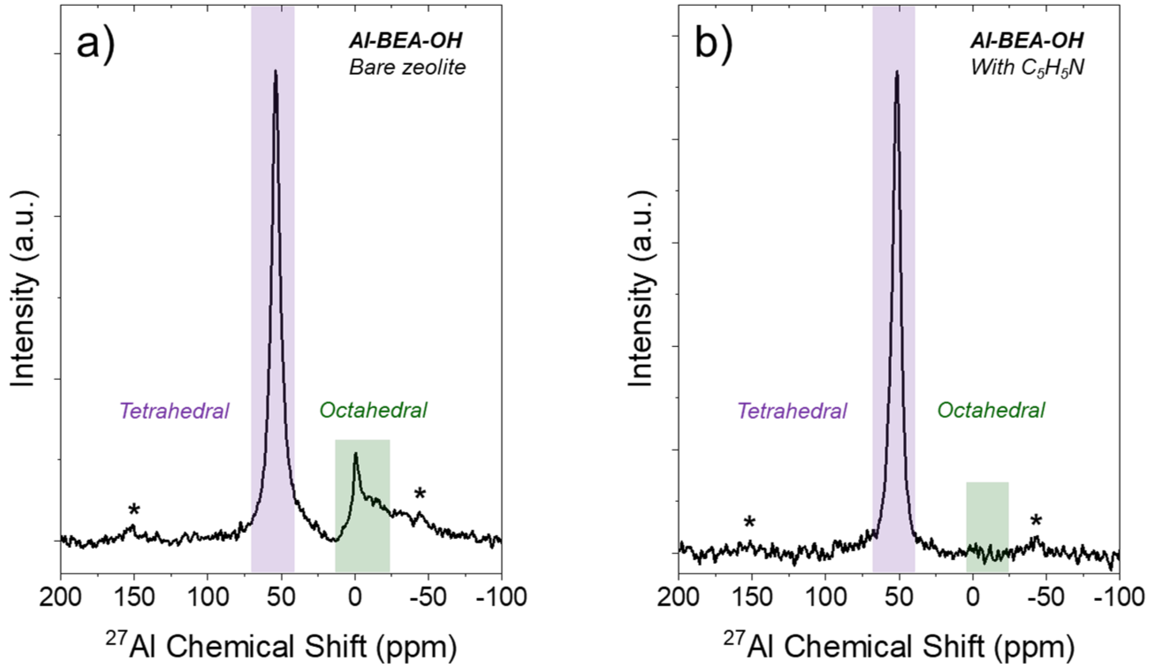

Figure S9. ^{27}Al NMR spectra for a) bare Al-BEA-OH, and b) Al-BEA-OH impregnated with C_5H_5N (298 K). The peaks representing tetrahedral and octahedral Al species are highlighted in purple and green, respectively. Asterisks represent spinning side-band features.

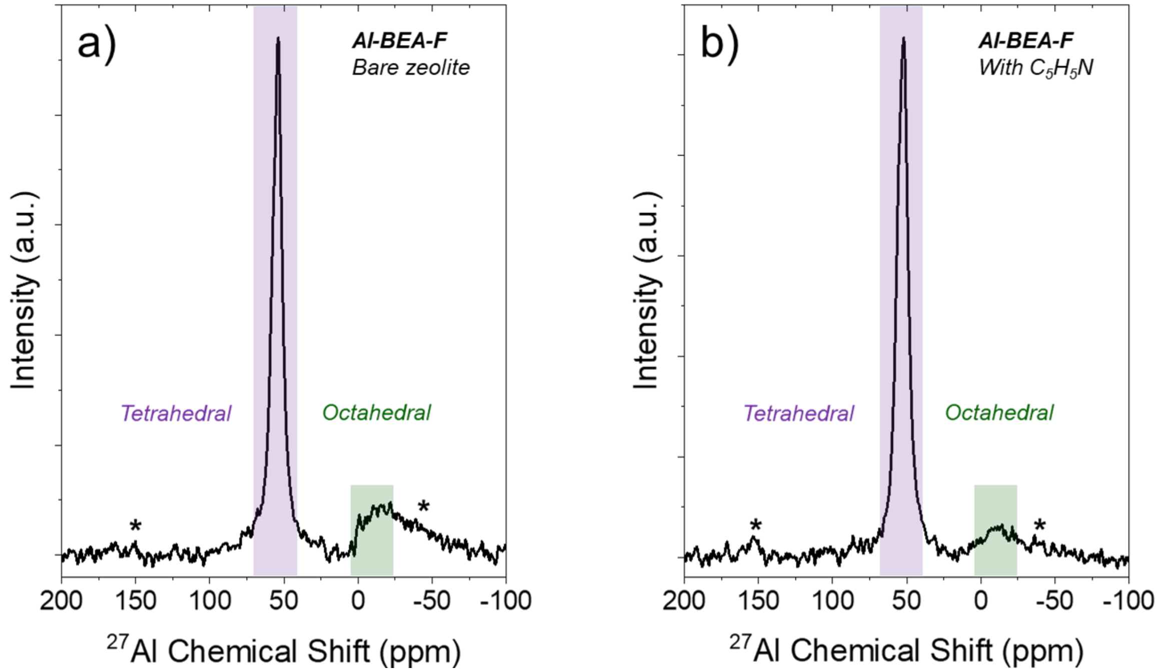

Figure S10. ^{27}Al NMR spectra for a) bare Al-BEA-F, and b) Al-BEA-F impregnated with C_5H_5N (298 K). The peaks representing tetrahedral and octahedral Al species are highlighted in purple and green, respectively. Asterisks represent spinning side-band features.

Figures S9 and S10 present the ^{27}Al NMR spectra for the Al-BEA materials in the presence and absence of liquid pyridine (C_5H_5N). The percentage of octahedral Al was calculated from the ratio of the tetrahedral to octahedral peak area features (Table S3). Notably, the bare zeolites show greater percentages of octahedral Al than the materials impregnated with C_5H_5N. Liquid or vapor water (H_2O) binds to Al atoms tetrahedrally coordinated within the zeolite framework and give rise to octahedral ^{27}Al NMR features, which reflects the additional coordination of two H_2O molecules.[46-48] Here, we believe that adventitious H_2O adsorbed from the atmosphere contributes to the larger quantities of octahedral Al observed in the bare zeolite materials in Figures S9 and S10. Previous works demonstrate that loading hydrated Brønsted acid zeolites with ammonia (NH_3) converts octahedral Al species back to tetrahedral Al sites, and consequently, the adsorption of a strong Brønsted base provides a more reliable strategy to report octahedral Al content without interference from H_2O.[48-50]

Motivated by the role of NH_3 shown in previous studies, we impregnated the Al-BEA materials with liquid C_5H_5N to revert hydrated Al species back to tetrahedral Al atoms. Table S3 shows that the C_5H_5N-incorporated materials show lower quantities of octahedral Al than the bare materials, but Al-BEA-OH and Al-BEA-F still contain non-zero quantities of octahedral Al. The ^{27}Al NMR spectra give octahedral Al percentages of 4 and 10% for Al-BEA-OH and Al-BEA-F, respectively. These values align closely with those obtained from active site titrations (Section S4, *vide infra*), which yield octahedral Al percentages of 3% and 8% for Al-BEA-OH and Al-BEA-F, respectively. Overall, the spectra in Figures S9 and S10 support that Al-BEA-OH and Al-BEA-F contain minor quantities of octahedral Al that do not convolute the kinetics reported in the main text.

Catalyst	% tetrahedral Al (bare zeolite)	% tetrahedral Al (with C_5H_5N)	Active Metal % (site titrations)
Al-BEA-OH	88	96	97 ± 4
Al-BEA-F	78	90	92 ± 3

Table S3. Percentage of tetrahedral Al measured from ^{27}Al NMR spectra (Figures S9 and S10) and active site titrations (see Section S4).

S1.8 Scanning Electron Micrographs of M-BEA Catalysts

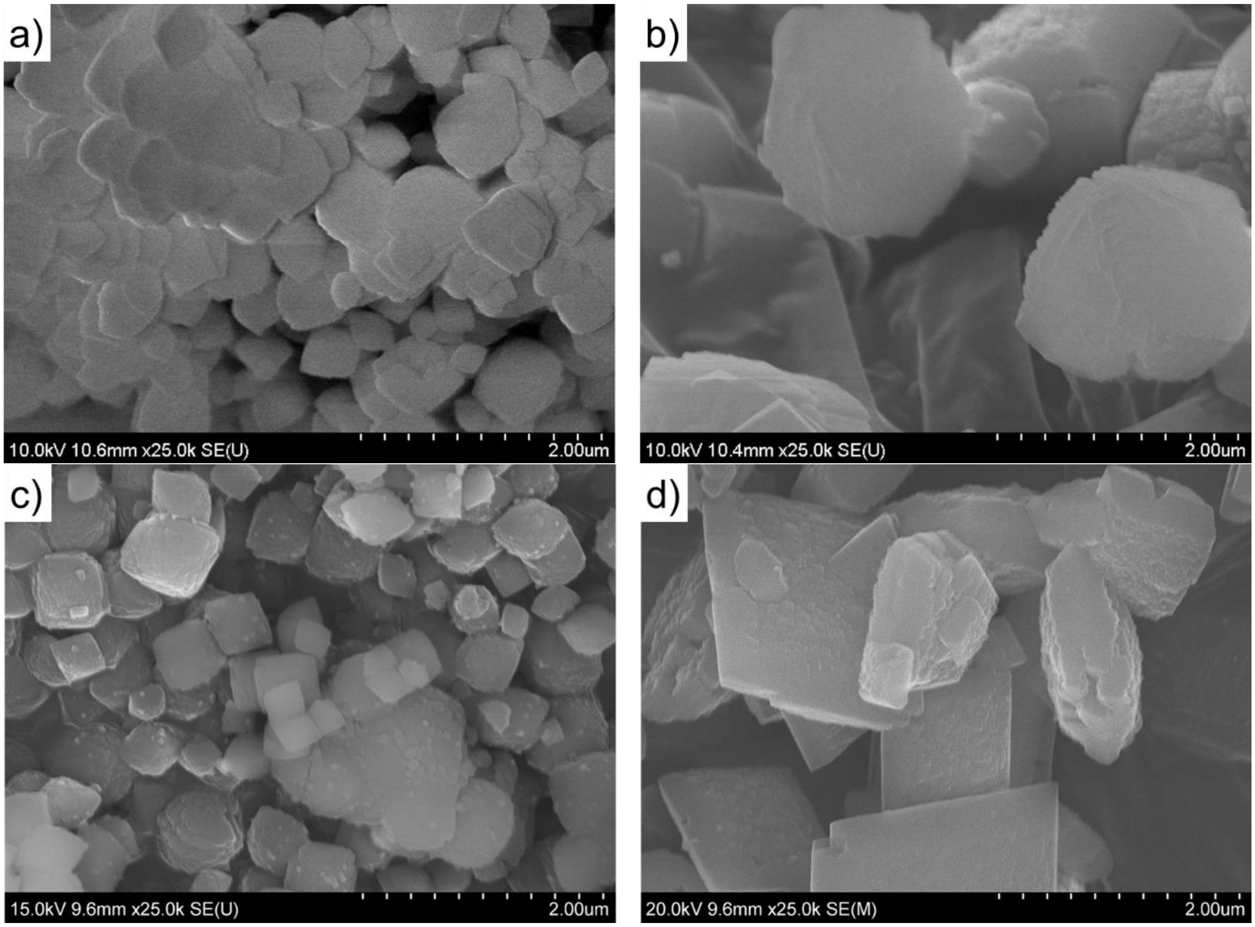

Figure S11. Representative high resolution scanning electron micrograph images of a) Al-BEA-OH, b) Al-BEA-F, c) Zr-BEA-OH, d) Zr-BEA-F.

The particle diameters of the M-BEA materials were estimated from scanning electron microscopy (SEM), with representative SEM images shown in Figure S11. Al-BEA-OH and Zr-BEA-OH both possess particles with mean diameters of 0.5 ± 0.2 μm, while Al-BEA-F and Zr-BEA-F show mean diameters of 2.0 ± 0.5 μm. The larger particle size of materials prepared in fluoride media compared to hydroxide media is consistent with previous reports over Ti-MFI[51-52] and Al-BEA[53-56] materials.

The differences in particle size can lead to internal mass transfer artifacts. However, we exclude this possibility because site titration experiments with 1,2-diphenyl-1,2-ethylenediamine show that all M-BEA materials possess approximately 100% active metal atoms (Figure S18, *vide infra*). Therefore, differences in C_4H_8O ring-opening rates between M-BEA-OH and M-BEA-F materials do not stem from differences in the particle diameter.

S1.9 Ar Adsorption Isotherms over M-BEA Materials

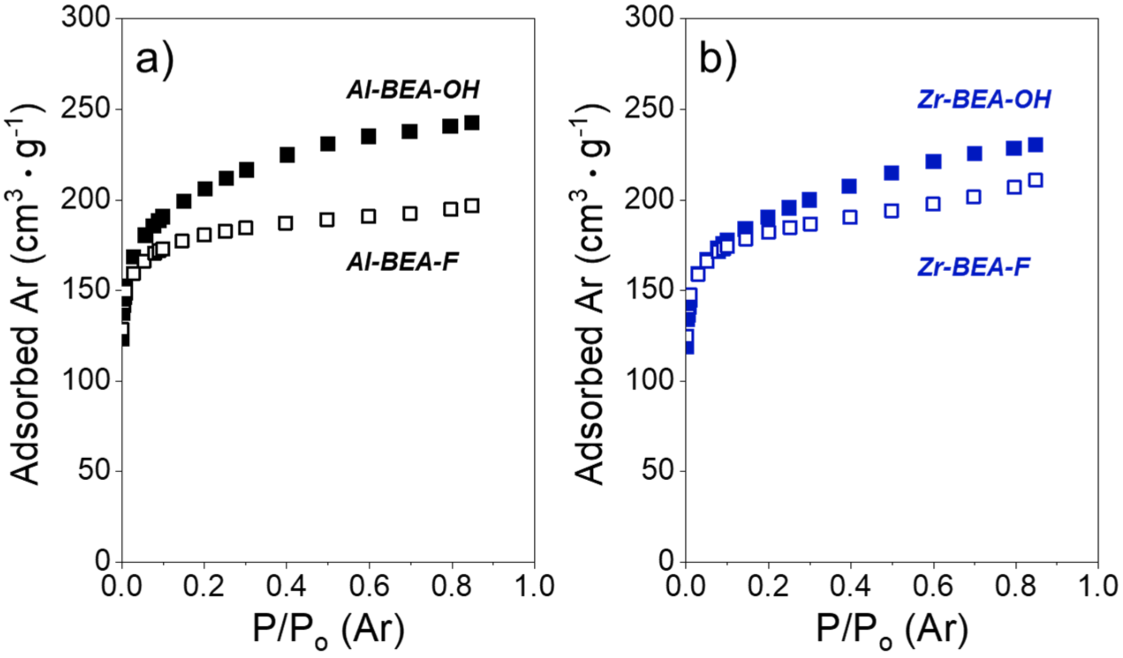

Figure S12. Ar (87 K) adsorption isotherms over hydrophilic (solid points) and hydrophobic (hollow) a) Al-BEA and b) Zr-BEA. The samples were degassed under dynamic vacuum prior to adsorption (< 7×10^{-4} Pa, 673 K, 3 h).

The BET surface areas and micropore volumes reported in Table 1 of the main text were calculated from the adsorption isotherms in Figure S12. The values in Table 1 are consistent with previous reports of surface areas[57-59] and micropore volumes[58, 60-63] of *BEA zeolites, supporting that M-BEA-OH and M-BEA-F possess the *BEA framework.

Al-BEA and Zr-BEA show similar surface area and volume magnitudes for both hydrophilic and hydrophilic catalysts. The BET surface area of the M-BEA-OH materials exceeds M-BEA-F by less than 1.2 times, while the micropore volume of the M-BEA-F materials is ~1.2 times greater than M-BEA-OH. While the external surface areas of the M-BEA-OH materials are ~2 times greater than M-BEA-F, the total pore volumes (micropore + mesopore) differ by less than 1.25 times between M-BEA-OH and M-BEA-F zeolites (Table S4).

Catalyst	External Surface Area (m^2 g^{-1})	Total Pore Volume (cm^3 g^{-1})
Al-BEA-OH	268	0.310
Al-BEA-F	132	0.252
Zr-BEA-OH	231	0.294
Zr-BEA-F	142	0.269

Table S4. External surface areas and total pore volumes obtained from Ar physisorption isotherms (Figure S12).

S2. Calculation of Epoxide and Product Sensitivity Factors

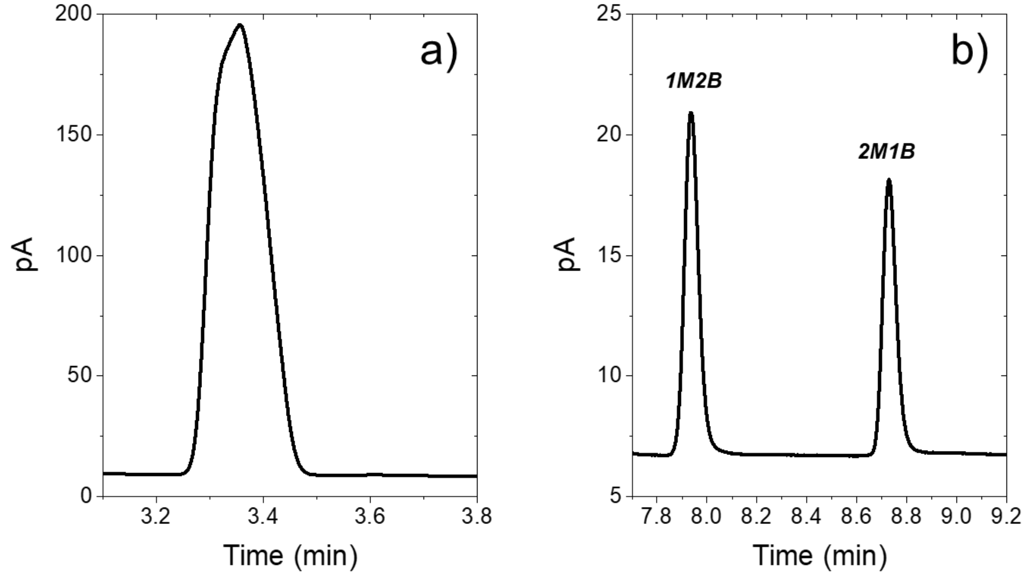

Figure S13. Raw GC chromatograms zoomed in to show peaks corresponding to (a) C_4H_8O, and (b) 1-methoxy-2-butanol and 2-methoxy-1-butanol formed by reaction of C_4H_8O with CH_3OH over Zr-BEA-OH for 1 h (0.005 M C_4H_8O, 6 M CH_3OH, CH_3CN, 308 K).

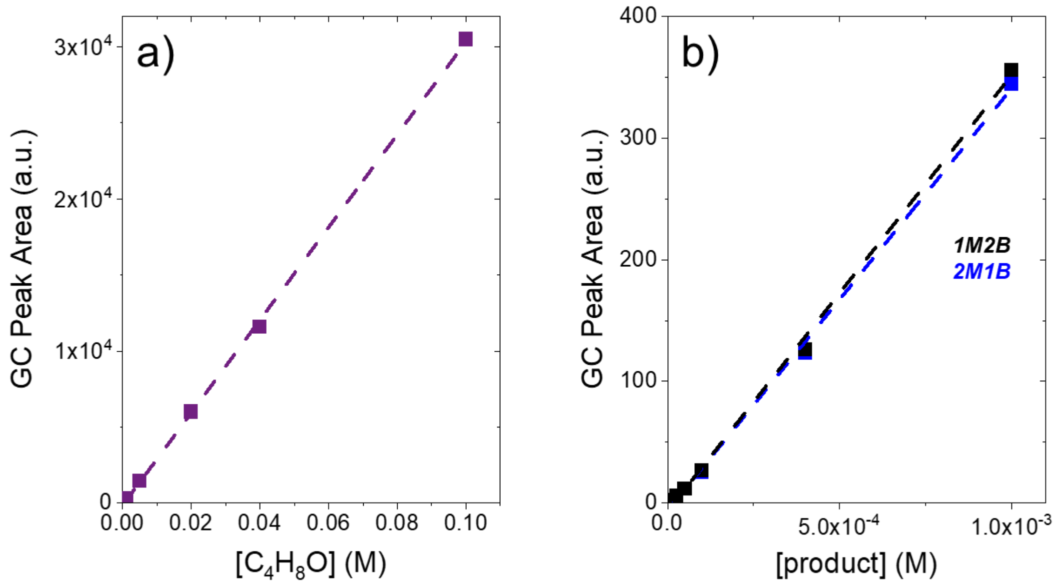

Figure S14. Integrated GC peak areas as a function of concentration for (a) C_4H_8O, and (b) 1-methoxy-2-butanol (black) and 2-methoxy-1-butanol (blue) in CH_3CN.

Figure S13 demonstrates the high signal-to-noise ratio of the epoxide and product peaks from the GC chromatograms, as well as the peak separation between the ring-opening products. GC chromatograms were collected as a function of concentrations (Figure S14) to determine the sensitivity factors of the epoxide and products using the as-purchased chemicals (see Sections 2.3 and 2.4 of the main text for sources and purities). Table S5 reports the sensitivity factors, which equal the slope of the calibration curve fit lines in Figure S14.

Compound	Sensitivity Factor (peak area M^{-1})
C_4H_8O	302810
1M2B	349380
2M1B	338550

Table S5. Sensitivity factors determined from calibration curves in Figure S14.

During reactions, the concentration of the products was calculated with the integrated peak areas and measured sensitivity factors:

$$[product] = \frac{GC\ Peak\ Area}{Sensitivity\ Factor} \quad \text{(S2)}$$

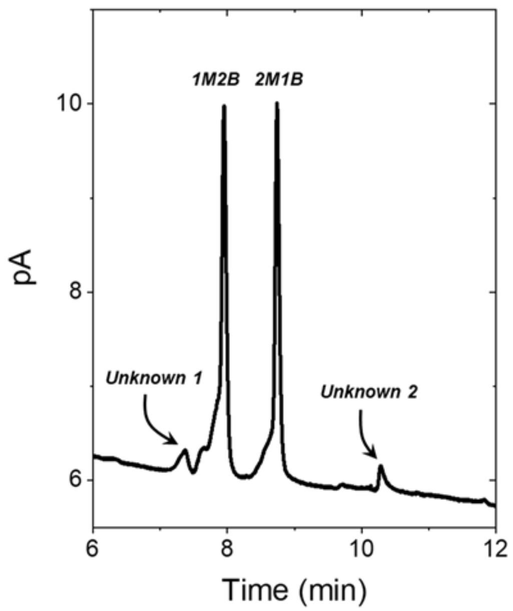

Figure S15. Raw GC chromatograms zoomed in to show peaks corresponding to 1-methoxy-2-butanol, 2-methoxy-1-butanol, and unknown peaks that appear during the reaction of C_4H_8O with CH_3OH over Al-BEA-F for 1 h (0.005 M C_4H_8O, 0.025 M CH_3OH, CH_3CN, 303 K).

Figure S15 presents a GC chromatogram from the reaction of C_4H_8O with CH_3OH, where unknown peaks grow at 7.4 and 10.3 minutes. We have ruled out many possible products that may form through side reactions of C_4H_8O because these species appear at different retention times than the unknown peaks, as shown in Table S6. As mentioned in Section 2.3 of the main text, the unknown peaks form without CH_3OH present, ruling out dimethoxybutane ($C_6H_{14}O_2$) and secondary oligomerization products as possible side products. We were unable to obtain 1,2-butylene carbonate ($C_5H_8O_3$) or $C_6H_{10}ON$ (from reaction of C4H8O with CH3CN) samples to check retention times, but these are two plausible side products that likely appear at similar retention times to the ring-opening products ($C_5H_{12}O_2$).

Compound	GC Retention Time (min)
1M2B	7.9
2M1B	8.7
**Unknown 1**	**7.4**
**Unknown 2**	**10.3**
Butanone	3.9
1-butanol	5.4
2-butanol	4.4
Butyraldehyde	3.8
Crotonaldehyde	4.6
Crotyl alcohol	5.1
1,2-butanediol	9.5
Dibutyl ether	11.2

Table S6. Measured GC retention times for ring-opening products, unknown peaks, and plausible side products.

S3. Hot Filtrations and Madon-Boudart Tests to Identify Active Centers for Ring-Opening

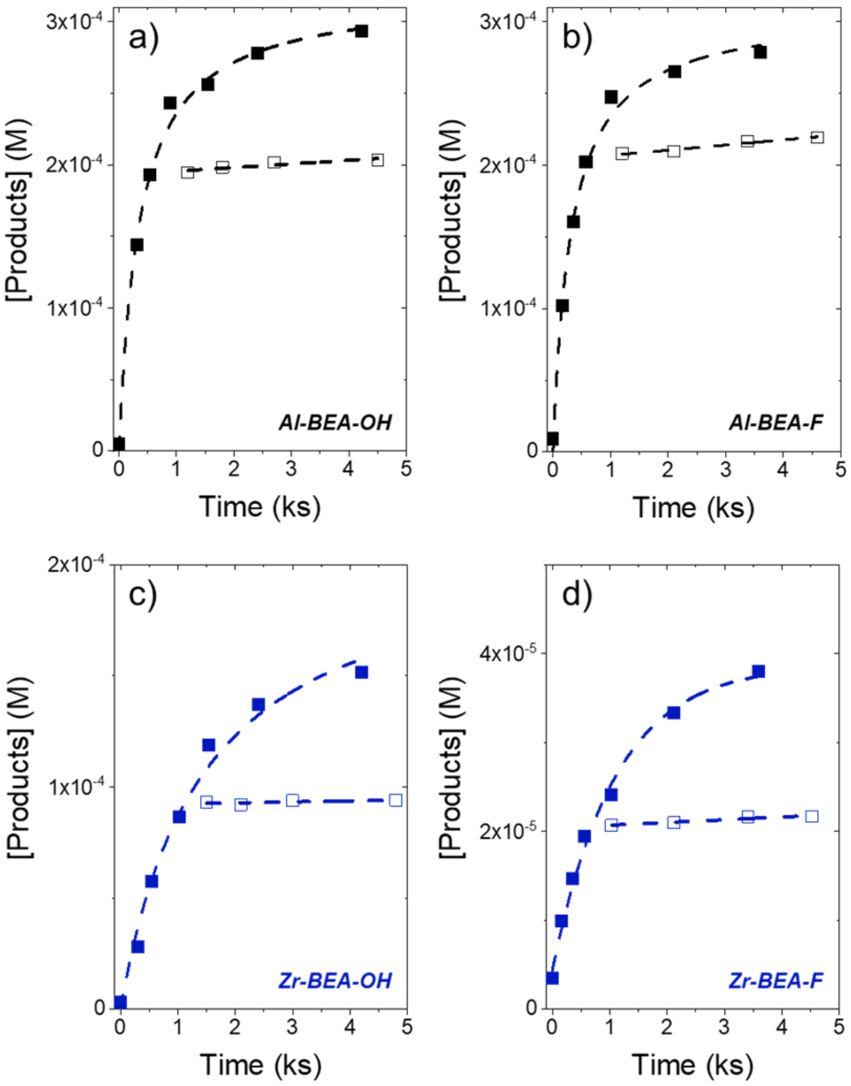

Figure S16. Measured total product concentrations (sum of [1M2B] and [2M1B]) as functions of time during hot filtration tests (0.005 M C_4H_8O, 6 M CH_3OH, CH_3CN solvent, 308 K) over (a) Al-BEA-OH, (b) Al-BEA-OH, (c) Zr-BEA-OH, and (d) Zr-BEA-F. The hollow points represent epoxide concentrations from aliquots that were separated from the catalyst after ~10-15 min, while solid points indicate concentrations from aliquots that were kept in contact with the catalyst over the course of the reaction.

Figure S16 displays the results of the hot filtration tests for all M-BEA. For all M-BEA, product formation ceases after separating the solution from the catalyst (hollow points). This result provides evidence that metal atoms do not leach from the *BEA framework, and the active sites remain within the framework during catalysis.

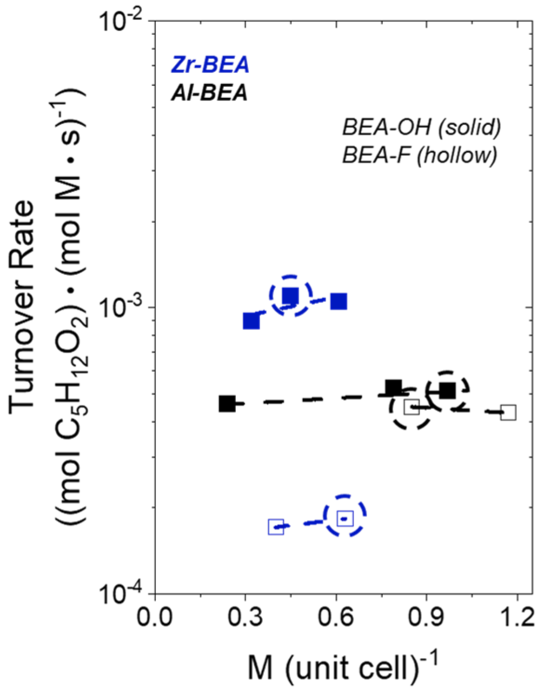

Figure S17. Total product formation rates from C_4H_8O ring-opening with CH_3OH (0.02 M C_4H_8O, 0.4 M CH_3OH, CH_3CN solvent, 308 K) as a function of active metal loading over hydrophilic (solid points) and hydrophobic (hollow) Zr- (blue) and Al-BEA (black) materials. The circled points signify the catalyst used for this study.

Figure S17 presents turnover rates as a function of metal loading for each M-BEA. Rates depend negligibly on metal loading within the range of loadings examined for all materials, ruling out contributions from intrapore mass transfer to turnover rate measurements.

Table S7 compares product formation rates per gram of catalyst for the catalysts used in this study and a dealuminated *BEA zeolite (Si-BEA-OH). The Si-BEA-OH materials shows formation rates 13 times less than the least active catalyst in this study (Zr-BEA-F), providing evidence that $(SiOH)_x$ and Si-O-Si functions do not contribute significantly to the calculated turnover rates for C_4H_8O ring-opening over M-BEA materials. Instead, C_4H_8O ring-opening occurs primarily at the active Al or Zr sites within M-BEA.

Catalyst	Product Formation Rate $(mol\ C_5H_{12}O_2) \cdot (g_{cat} \cdot s)^{-1})$
Al-BEA-OH	1.35×10^{-7}
Al-BEA-F	1.17×10^{-7}
Zr-BEA-OH	2.38×10^{-7}
Zr-BEA-F	3.89×10^{-8}
Si-BEA-OH	2.88×10^{-9}

Table S7. Product formation rates per gram of catalyst for the M-BEA catalysts used in this study compared to Si-BEA-OH (0.02 M C_4H_8O, 0.4 M CH_3OH, CH_3CN solvent, 308 K). Rates for Si-BEA-OH were normalized by the number of $(SiOH)_x$ groups in the material (3.047, see Section S2.5)

S4. Counting Active Sites with 1,2-Diphenyl-1,2-Ethylenediamine Titrations

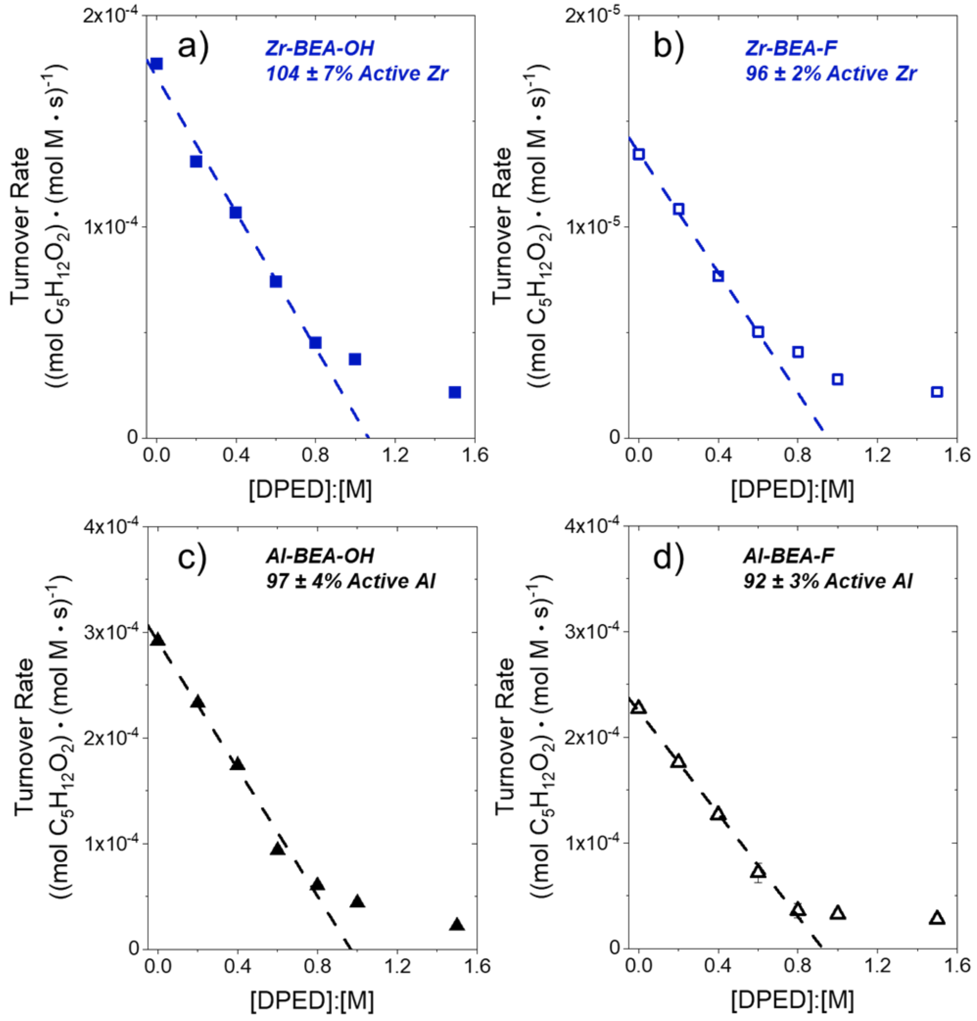

Figure S18. Total product formation rates from C_4H_8O ring-opening with CH_3OH (0.005 M C_4H_8O, 0.4 M CH_3OH, CH_3CN solvent, 308 K) as a function of 1,2-diphenyl-1,2-ethylenediamine (DPED) to active metal ratio over hydrophilic (solid points) and hydrophobic (hollow) Zr- (blue) and Al-BEA (black) materials.

Recently, phosphonic acids[64-67] have been employed as titrants during liquid phase reactions to count or distinguish acid sites in Lewis acid materials. Amines have been used to probe both Lewis acid[68-69] and Brønsted acid sites[70-71] for similar purposes. Therefore, we selected 1,2-diphenyl-1,2-ethylenediamine (DPED) as a titrant that could poison active sites in both Lewis and Brønsted acidic M-BEA. A line was fit to the linear portion of the plots in Figure S18 and extrapolated to the x-axis to estimate the number of active sites present in the M-BEA materials. All materials show greater than 90% active metal atoms, providing evidence that differences in the quantity of active metal do not contribute to rate differences between M-BEA.

The change in concentration of DPED during the reactions in Figure S18 was quantified by GC-FID. A GC calibration curve was developed for DPED, from which the moles of DPED in the liquid phase before and

at the completion of the reaction was determined with GC peak areas. A separate DPED adsorption measurement was carried out in a dealuminated *BEA catalyst (Si-BEA) to estimate contributions from DPED bound to $(SiOH)_x$ and pore walls (i.e., Si-O-Si). The DPED volume for the Si-BEA control was chosen to match the largest volume used in an experiment in Figure S18. Noticeably, the liquid phase DPED concentration decreases by more than 97% in all experiments in Figure S18 and the Si-BEA control after 1 h. This result suggests that any DPED that does not coordinate to Lewis or Brønsted acid sites can bind to $(SiOH)_x$ or Si-O-Si linkages. Nevertheless, the suppression of rates and extrapolated linear fits in Figure S18 provide strong evidence that DPED preferentially binds to Lewis and Brønsted acid sites, suppressing C_4H_8O ring-opening over M-BEA.

S5. Formation Rates of Individual Products of C_4H_8O Ring-Opening with CH_3OH

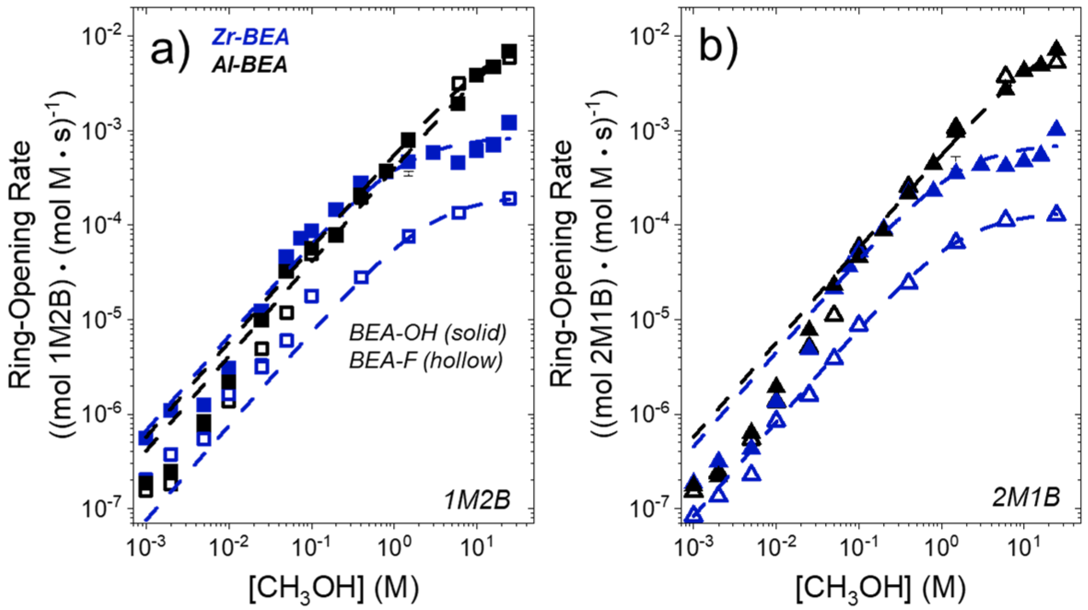

Figure S19. Formation rates of a) 1-methoxy-2-butanol (1M2B) and b) 2-methoxy-1-butanol (2M1B) products from C_4H_8O ring-opening with CH_3OH as functions of CH_3OH concentration over hydrophilic (solid points) and hydrophobic (hollow) Zr- (blue) and Al-BEA (black) materials (0.005 M C_4H_8O, CH_3CN solvent, 308 K).

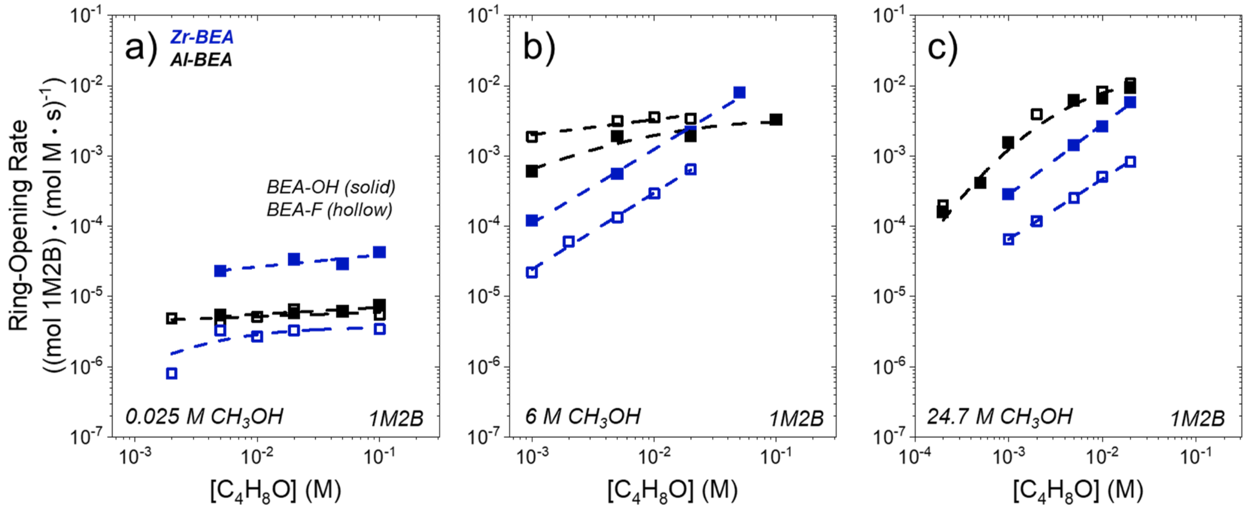

Figure S20. Formation rates of 1-methoxy-2-butanol (1M2B) from C_4H_8O ring-opening with CH_3OH as a function of C_4H_8O concentration at a) 0.025 M CH_3OH, b) 6 M CH_3OH, or c) neat (24.7 M) CH_3OH (CH_3CN solvent, 308 K) over hydrophilic (solid points) and hydrophobic (hollow) Zr- (blue) and Al-BEA (black) materials.

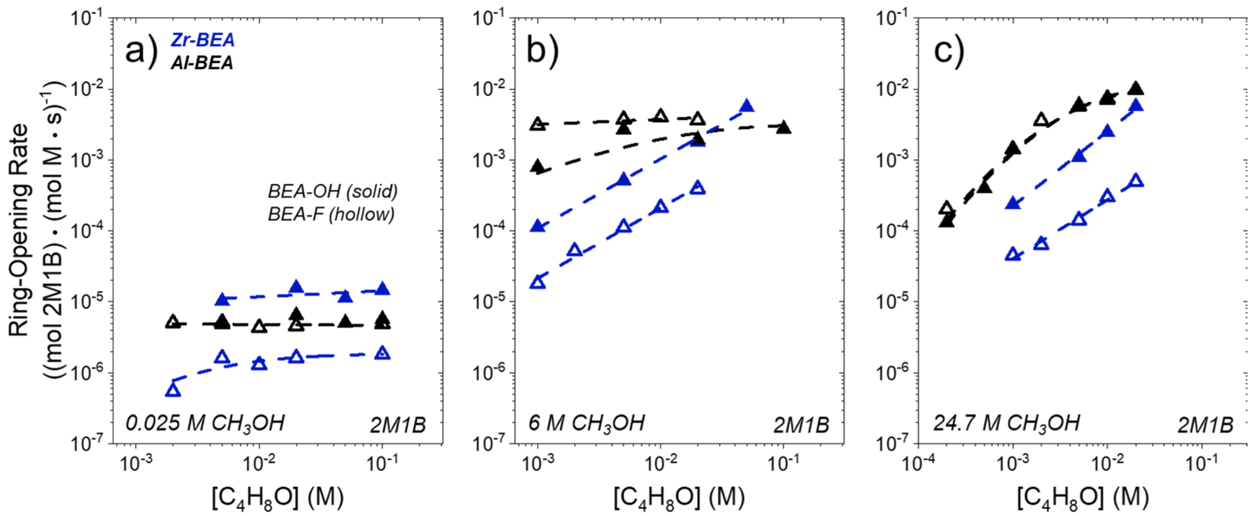

Figure S21. Formation rates of 2-methoxy-1-butanol (2M1B) from C_4H_8O ring-opening with CH_3OH as a function of C_4H_8O concentration at a) 0.025 M CH_3OH, b) 6 M CH_3OH, or c) neat (24.7 M) CH_3OH (CH_3CN solvent, 308 K) over hydrophilic (solid points) and hydrophobic (hollow) Zr- (blue) and Al-BEA (black) materials.

Figures S19 to S21 present the formation rates of the individual products from C_4H_8O ring-opening over M-BEA (total ring-opening rates shown in Figures 1 and 2 of the main text). In all M-BEA and solvent composition combinations, both products show nearly identical formation rate dependences on [C_4H_8O] and [CH_3OH]. The similar rate dependences provide compelling evidence that each product forms from identical reaction pathways and reactive intermediates, and differences in the stability of transition states between the products drive changes in regioselectivities.

S6. Derivation of Rate Expression for C_4H_8O Ring-Opening with CH_3OH over M-BEA

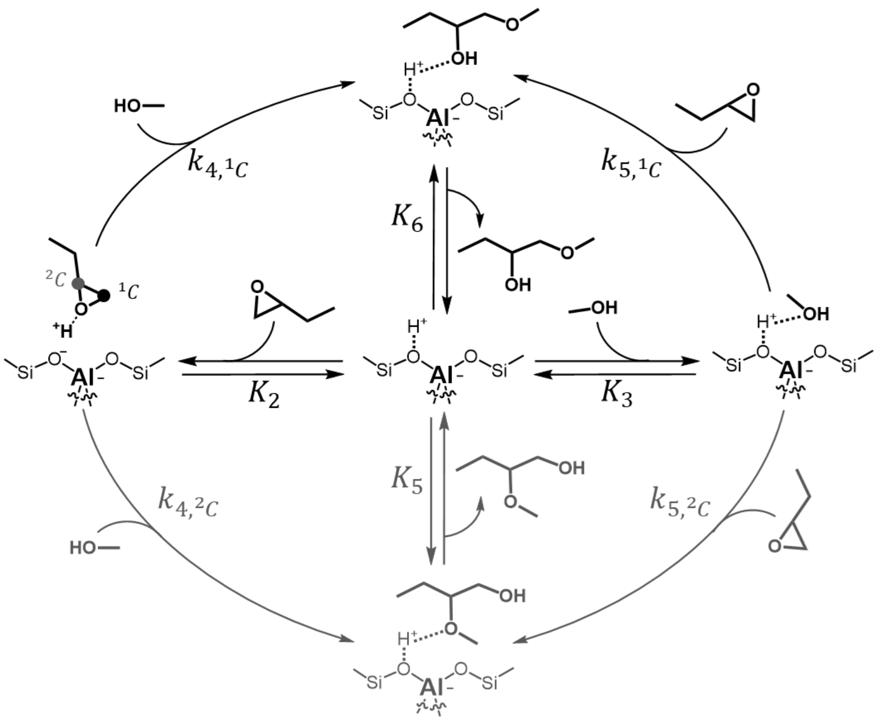

Scheme S1. Proposed catalytic cycle for C_4H_8O ring-opening with CH_3OH in CH_3CN solvent over Al-BEA zeolites. All adsorption steps are currently assumed as reversible. Steps to form 1M2B and 2M1B are denoted as ${}^1C$ or ${}^2C$, to signify nucleophilic attack on the primary and secondary carbons in the epoxide ring, respectively. For brevity, we do not show the reversible adsorption of CH_3CN molecules (step 1) in this cycle.

Scheme 2 in the main text and Scheme S1 above present proposed catalytic cycles for the ring-opening of C_4H_8O with CH_3OH over Lewis and Brønsted acidic M-BEA materials, respectively. Each mechanism begins with the adsorption of CH_3CN (step 1), C_4H_8O (step 2), or CH_3OH (step 3). The 1M2B or 2M1B products may then form through one of two plausible kinetically relevant steps (4 and 5), where a liquid-phase CH_3OH or C_4H_8O molecule may then react with adsorbed C_4H_8O or CH_3OH, respectively. The products then desorb in a reversible step to complete the cycle, with steps 6 and 7 accounting for the individual products that can form.

The reaction likely does not proceed as a bimolecular Langmuir-Hinshelwood reaction between surface-bound CH_3OH and C_4H_8O because this would lead to squared dependences on the CH_3OH and C_4H_8O terms in the denominator at sufficiently high coverages of either species. Rates would eventually decrease with increasing concentrations of CH_3OH and C_4H_8O in a bimolecular surface reaction. Rates show linear or zero order dependences over all conditions studied here, providing support that the reaction proceeds through an Eley-Rideal mechanism as illustrated in Scheme 2 and Scheme S1.

Epoxide ring-opening rates for the two possible reaction paths can be modeled with Equation 2 from the main text:

$$r_{RO} = r_4 + r_5 = k_4[C_4H_8O^*][CH_3OH] + k_5[C_4H_8O][CH_3OH^*] \quad \text{(S3)}$$

where k_4 and k_5 are the sum of the rate constants to form each product in steps 4 ($k_{4,^1C}$, $k_{4,^2C}$) and 5 ($k_{5,^1C}$, $k_{5,^2C}$) in Scheme S1, and [C_4H_8O*] and [CH_3OH*] represent the number of bound intermediates from each reactant. We apply the pseudo-state hypothesis to the respective bound intermediates, which gives:

$$r_{RO,C_4H_8O^*} = \frac{k_2k_4[C_4H_8O][CH_3OH][*]}{k_{-2}+k_4[CH_3OH]} + \frac{k_3k_5[C_4H_8O][CH_3OH][*]}{k_{-3}+k_5[C_4H_8O]} \quad \text{(S4)}$$

The total number of active sites in each M-BEA ([L]) can be written as:

$$[L] = [*] + [CH_3CN^*] + [CH_3OH^*] + [C_4H_8O^*] + [1M2B^*] + [2M1B^*] \quad \text{(S5)}$$

Where $[*]$ is the number of unoccupied active sites and the other terms represent the number of adsorbed solvent, reactant, and product molecules. Substituting in terms for the solvent, product, and reactant concentrations, rate constants, and equilibrium constants yields:

$$[L] = [*]\left(1 + K_1[CH_3CN] + \frac{k_2[C_4H_8O]}{k_{-2}+k_4[CH_3OH]} + \frac{k_3[CH_3OH]}{k_{-3}+k_5[C_4H_8O]} + \frac{k_4[CH_3OH]+k_5[C_4H_8O]+k_{-6}[1M2B]}{k_6} + \frac{k_4[CH_3OH]+k_5[C_4H_8O]+k_{-7}[2M1B]}{k_7}\right) \quad \text{(S6)}$$

Using Equation S6 to substitute an expression for $[*]$ in Equation S4 yields a full rate expression (Equation 3 in the main text):

$$\frac{r_{RO}}{[L]} = \frac{\frac{k_2k_4[C_4H_8O][CH_3OH]}{k_{-2}+k_4[CH_3OH]} + \frac{k_3k_5[C_4H_8O][CH_3OH]}{k_{-3}+k_5[C_4H_8O]}}{1+K_1[CH_3CN]+\frac{k_2[C_4H_8O]}{k_{-2}+k_4[CH_3OH]}+\frac{k_3[CH_3OH]}{k_{-3}+k_5[C_4H_8O]}+\frac{k_4[CH_3OH]+k_5[C_4H_8O]+k_{-6}[1M2B]}{k_6}+\frac{k_4[CH_3OH]+k_5[C_4H_8O]+k_{-7}[2M1B]}{k_7}} \quad \text{(S7)}$$

Rates depend linearly on [CH_3OH] and weakly on [C_4H_8O] at ratios of [CH_3OH] to [C_4H_8O] less than 40 over each M-BEA, implicating active sites saturated with a C_4H_8O-derived species. In this case, the [C_4H_8O*] term dominates in the denominator of Equation S7, allowing all other terms to be canceled out:

$$\frac{r_{RO}}{[L]} = \frac{k_2k_4[CH_3OH](k_{-3}+k_5[C_4H_8O])+k_3k_5[CH_3OH](k_{-2}+k_4[CH_3OH])}{k_2(k_{-3}+k_5[C_4H_8O])} \quad \text{(S8)}$$

This equation predicts a superlinear dependence of rates on [CH_3OH] and a weak dependence on [C_4H_8O] at low ratios of [CH_3OH] to [C_4H_8O]. However, the form of this equation matches the trends observed in Figures 1 and 2 of the main text at ratios of [CH_3OH] to [C_4H_8O] up to ~40 in Zr-BEA and ~1000 over Al-BEA only when the first term in the numerator dominates, which leads to the following:

$$\frac{r_{RO}}{[L]} = k_4[CH_3OH] \quad \text{(S9)}$$

Neglecting the second term implies that the value of k_2k_4 far exceeds k_3k_5. C_4H_8O shows much larger adsorption enthalpies (~ -50 to -100 kJ mol^{-1}) than CH_3OH (~ 0 to -5 kJ mol^{-1}) over M-BEA, measured by ITC (*vide infra*, Section S12). These comparisons suggest that values for the equilibrium constants for binding C_4H_8O (K_2) exceed those for CH_3OH (K_3), which implies that C_4H_8O adsorbs more readily than CH_3OH (i.e., k_2 >> k_3). The much greater values of k_2 relative to k_3 may lead to the first term in the numerator of Equation S8 dominating up to [CH_3OH] to [C_4H_8O] ratios of ~40 in Lewis acids and ~1000 over Al-BEA, even if k_4 and k_5 have similar values. Consequently, these comparisons indicate that the difference in adsorption strength between C_4H_8O and CH_3OH leads to rate trends that agree with the form of Equation 4 at sufficiently low ratios of [CH_3OH] to [C_4H_8O]. These assumptions and adsorption enthalpy measurements justify the simplification of Equation S8 to Equation S9 and the conclusion that C_4H_8O-derived species dominate active sites at these conditions.

At sufficiently high ratios of [CH_3OH] to [C_4H_8O] (> 500 for Lewis acids and > 5000 for Al-BEA), ring-opening rates shift to a weak dependence on [CH_3OH] and linear dependence on [C_4H_8O]. The [CH_3OH^*] term now dominates in the denominator of Equation S7:

$$\frac{r_{RO}}{[L]} = \frac{k_2 k_4 [C_4H_8O](k_{-3} + k_5[C_4H_8O]) + k_3 k_5 [C_4H_8O](k_{-2} + k_4[CH_3OH])}{k_3(k_{-2} + k_4[CH_3OH])} \quad \text{(S10)}$$

Here, the numerator simplifies to a form that matches the linear rate dependence on [C_4H_8O] and weak dependence on [CH_3OH] under greater ratios of [CH_3OH]:[C_4H_8O] when the second term of the numerator dominates, which gives:

$$\frac{r_{RO}}{[L]} = k_5[C_4H_8O] \quad \text{(S11)}$$

At sufficiently high ratios of [CH_3OH] to [C_4H_8O] (>500 in Lewis acids and ~5000 over Al-BEA), [CH_3OH] likely dominates over all terms in Equation S10, leading to a negligible value for the first term in the numerator. These assumptions lead to the form of Equation S11 in solutions with a large stoichiometric excess of CH_3OH, even if k_4 and k_5 possess values of a similar magnitude.

The rate expressions in Equations S9 and S11 account for all kinetic regimes observed as functions of reactant concentrations in Figures 1 and 2 of the main text.

S7. Excess Energies to Probe Liquid-Phase C_4H_8O and CH_3OH Stability

	C_4H_8O			CH_3OH		
Solvent	γ	H^{ε} (kJ mol^{-1})	G^{ε} (kJ mol^{-1})	γ	H^{ε} (kJ mol^{-1})	G^{ε} (kJ mol^{-1})
CH_3CN	2.23	1.01	2.08	2.64	2.39	2.53
6 M CH_3OH (in CH_3CN)	2.91	3.52	2.78	1.77	2.43	1.48
12 M CH_3OH (in CH_3CN)	2.85	3.20	2.72	1.29	0.9	0.66
CH_3OH	3.62	1.70	3.35	1	0	0

Table S8. Activity coefficients (γ), excess enthalpies (H^{ε}), and excess free energies (G^{ε}) calculated at 308 K in mixtures of CH_3CN and CH_3OH. Activity coefficients were obtained with the UNIFAC method on ChemCAD.

Table S8 presents the excess energies and activity coefficients for C_4H_8O and CH_3OH in mixtures of CH_3OH and CH_3CN at 308 K. The equations used to compute the excess energies from the activity coefficients are as follows:

$$G_j^{\varepsilon} = RT * ln(\gamma_j) \tag{S12}$$

$$H_j^{\varepsilon} = -RT^2 * \frac{d\ ln(\gamma_j)}{dT} \tag{S13}$$

where *j* represents C_4H_8O or CH_3OH here, R equals the ideal gas constant, and T represents temperature. The differential term $\left(\frac{d\ln(\gamma_j)}{dT}\right)$ was obtained by calculating activity coefficients between 303-323 K by increments of 5 K. The natural log of the activity coefficients was plotted against the temperature, and the slope of this line equaled the differential term.

Notably, γ values vary by factors of less than 2 and 3 for C_4H_8O and CH_3OH, respectively, across the range of solvent compositions examined for ring-opening and C_4H_8O adsorption. Furthermore, both H^{ε} and G^{ε} values span a range of less than 3 kJ mol^{-1} across solvent compositions. The weak dependences of these values on the mixing ratios of CH_3OH and CH_3CN support that the stability of reactive species and adsorbates in the liquid phase does not drive changes in ring-opening rates, activation enthalpies for ring-opening, and enthalpies of adsorption for C_4H_8O.

S8. Activation Enthalpy and Entropy Calculations and Supplemental Data

$\Delta H^{\ddagger}$ and $\Delta S^{\ddagger}$ values to form each ring-opening product were calculated using van't Hoff analysis. Applying transition state theory and substituting an expression for the apparent free energy to Equations S14 and S15 yields alternate rate expressions for ring-opening through either the bound C_4H_8O path (C_4H_8O MARI) or bound CH_3OH route (CH_3OH MARI):

$$\frac{r_x}{[L]} = \frac{k_B T}{h} \cdot \exp\left(-\frac{\Delta G_x^{\ddagger}}{RT}\right) \cdot [CH_3OH] \quad \text{(S14)}$$

$$\frac{r_x}{[L]} = \frac{k_B T}{h} \cdot \exp\left(-\frac{\Delta G_x^{\ddagger}}{RT}\right) \cdot [C_4H_8O] \quad \text{(S15)}$$

Where x represents the two possible products, 1M2B and 2M1B. Rearranging and substituting in the apparent activation enthalpy and entropy:

$$\ln\left(\frac{r_x}{[L]} \frac{h}{k_B T[CH_3OH]}\right) = \frac{1}{T}\left(\frac{-\Delta H_x^{\ddagger}}{R}\right) + \frac{\Delta S_x^{\ddagger}}{R} \quad \text{(S16)}$$

$$\ln\left(\frac{r_x}{[L]} \frac{h}{k_B T[C_4H_8O]}\right) = \frac{1}{T}\left(\frac{-\Delta H_x^{\ddagger}}{R}\right) + \frac{\Delta S_x^{\ddagger}}{R} \quad \text{(S17)}$$

The natural log term on the left side of Equations S16 and S17 was plotted against the inverse temperature for each product. A line was fit to this data (Figure S22), where the slope of this line equaled the negative of the apparent activation enthalpy divided by the ideal gas constant, and the y-intercept equaled the apparent activation entropy divided by the ideal gas constant (Table S9).

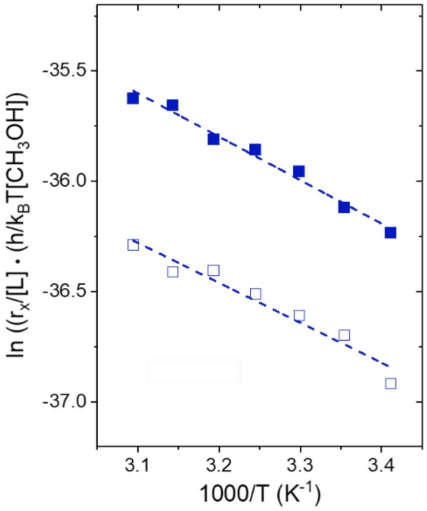

Figure S22. Representative Eyring plot for C_4H_8O ring-opening (0.005 M C_4H_8O, 0.025 M CH_3OH, CH_3CN solvent, 293-323 K) over Zr-BEA-OH to form 1-methoxy-2-butanol (solid points) and 2-methoxy-1-butanol (hollow points).

$\frac{-\Delta H_{1M2B}^{\ddagger}}{R}$	$\frac{\Delta S_{1M2B}^{\ddagger}}{R}$	$\frac{-\Delta H_{2M1B}^{\ddagger}}{R}$	$\frac{\Delta S_{2M1B}^{\ddagger}}{R}$
-2.0 ± 0.1	-29.5 ± 1.0	-1.8 ± 0.2	-30.7 ± 0.6

Table S9. Activation enthalpy and entropy to form each product in C_4H_8O ring-opening divided by the ideal gas constant, obtained from the slope and intercept, respectively, of Figure S22.

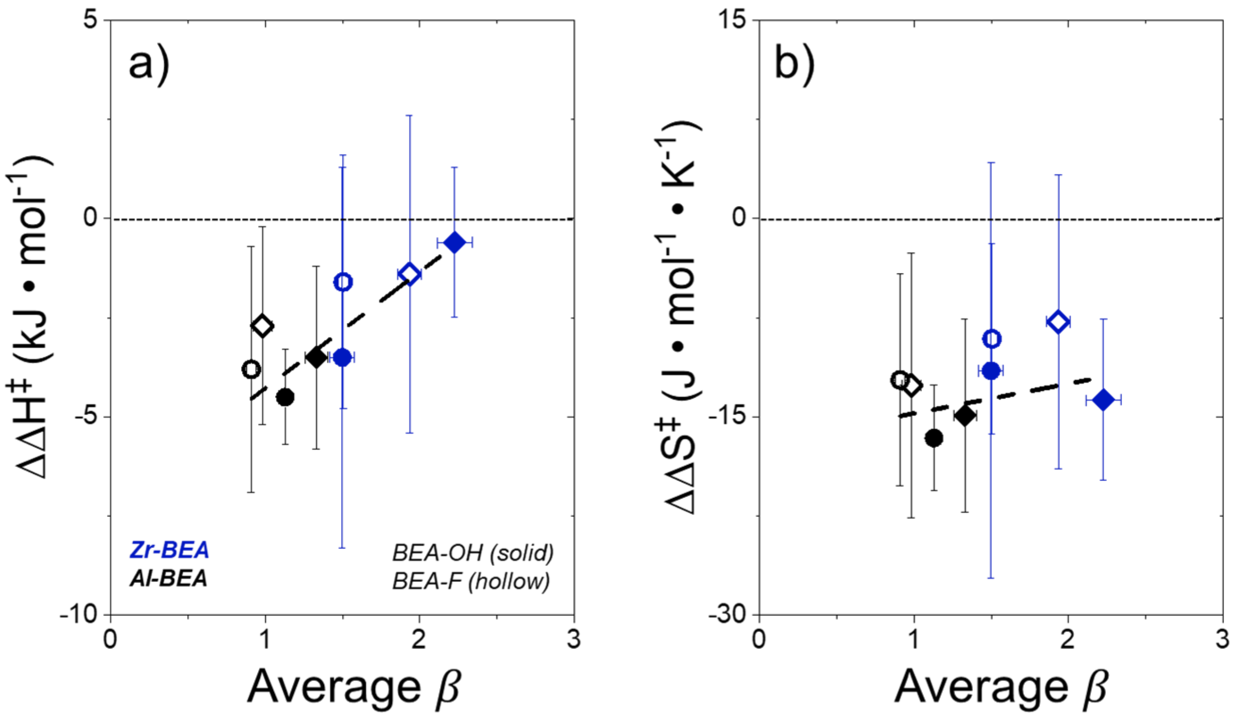

Figure S23. Values for a) $\Delta\Delta H^{\ddagger}$ and b) $\Delta\Delta S^{\ddagger}$ for C_4H_8O ring-opening and corresponding average values of β over hydrophilic (solid) and hydrophilic (hollow) Al- (black) and Zr-BEA (blue) materials from 298 – 323 K. Measurements were obtained in dilute CH_3OH (diamonds; 0.005 M C_4H_8O, 0.025 M CH_3OH in CH_3CN) and neat CH_3OH as solvent (circles; 1 M C_4H_8O, 24.7 M CH_3OH) with concentrations resulting in C_4H_8O*-covered active sites for all conditions.

Figure S23 presents differences in the activation enthalpies and entropies to form the products, deconvoluted from the activation barriers for total product formation presented in Figure 3 of the main text. These values represent the difference in the activation parameters for 2M1B relative to 1M2B:

$$\Delta\Delta H^{\ddagger} = H_{2M1B}{}^{\ddagger} - H_{1M2B}{}^{\ddagger} \quad \text{(S17)}$$

$$\Delta\Delta S^{\ddagger} = S_{2M1B}{}^{\ddagger} - S_{1M2B}{}^{\ddagger} \quad \text{(S18)}$$

Formation of 2M1B shows an increasing enthalpic preference (more negative $\Delta\Delta H^{\ddagger}$) with increasing [$CH_3OH$] and decreasing β values (Figure S23a). This trend supports that CH_3OH molecules stabilize the 2M1B transition state through hydrogen-bonding interactions. At the same time, the formation of the 2M1B transition state requires a greater entropic penalty (more negative $\Delta\Delta S^{\ddagger}$) regardless of catalyst identity or [CH_3OH] (Figure S23b). Overall, the differences in activation parameters between the products suggests that changing [CH_3OH] influences reaction regioselectivity through differences in solvent environment.

S9. Kinetic Measurements Under C_4H_8O-Covered Active Sites at High [C_4H_8O]

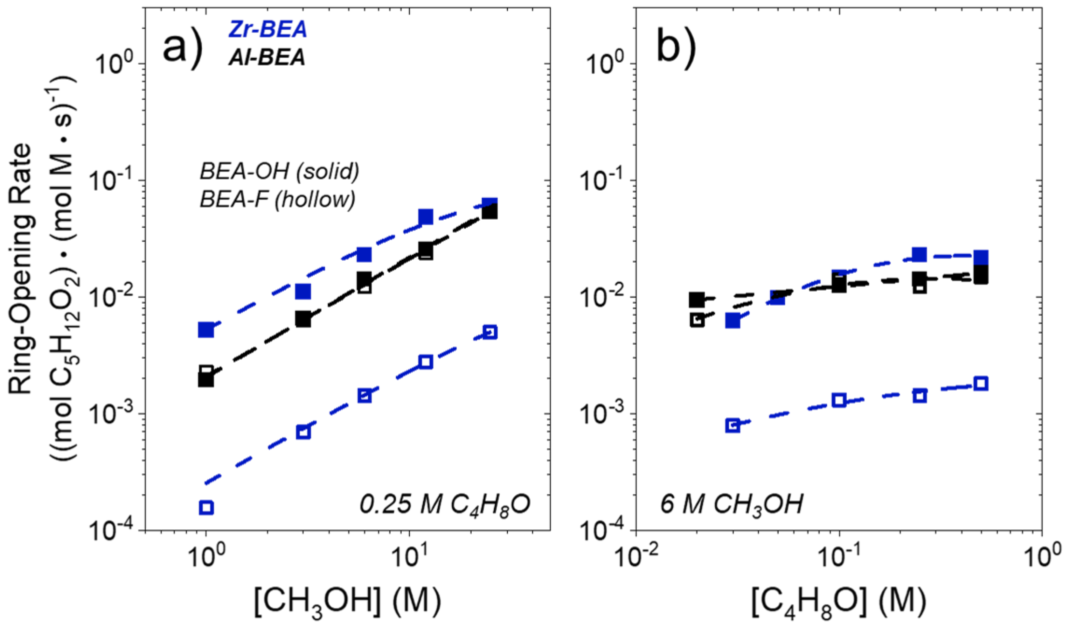

Figure S24. Turnover rates for C_4H_8O ring-opening with CH_3OH as functions of a) CH_3OH concentration at 0.25 M C_4H_8O (CH_3CN solvent, 308 K) and b) C_4H_8O concentration at 6 M CH_3OH (CH_3CN solvent, 308 K) over hydrophilic (solid points) and hydrophobic (hollow) Zr- (blue) and Al-BEA (black) materials.

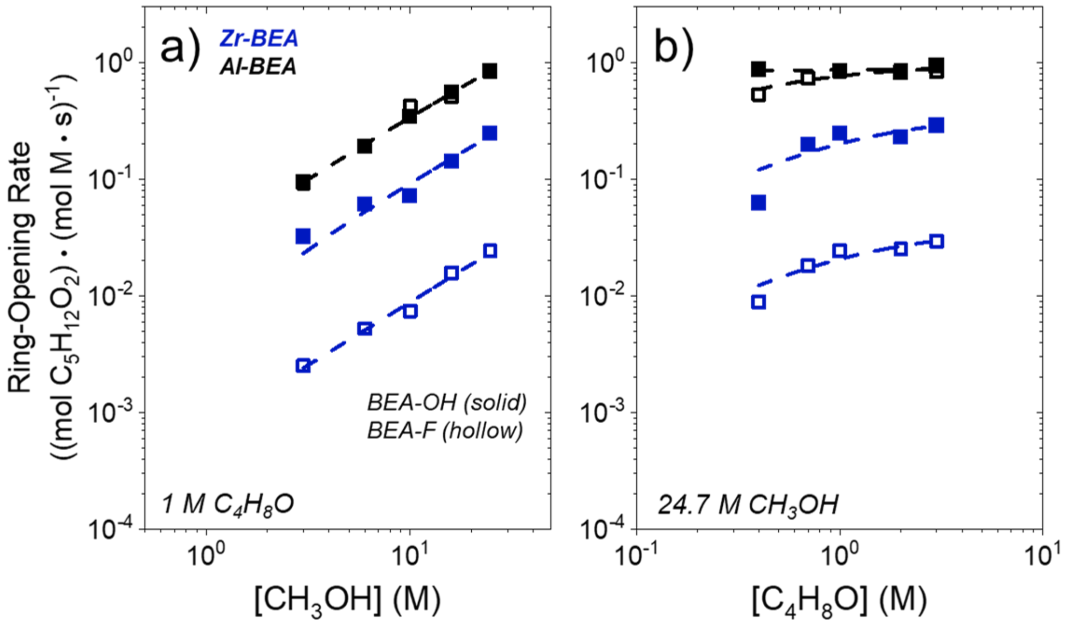

Figure S25. Turnover rates for C_4H_8O ring-opening with CH_3OH as functions of a) CH_3OH concentration at 1 M C_4H_8O (CH_3CN solvent, 308 K) and b) C_4H_8O concentration at 24.7 M CH_3OH (308 K) over hydrophilic (solid points) and hydrophobic (hollow) Zr- (blue) and Al-BEA (black) materials.

Figure S24 and Figure S25 present turnover rates for C_4H_8O ring-opening as a function of [CH_3OH] and [C_4H_8O] at high concentrations of [C_4H_8O]. All materials show a regime of first-order rate dependence on [CH_3OH], under which rates were then measured at varying [C_4H_8O]. Under this regime, materials show a corresponding zero-order rate dependence on [C_4H_8O], suggesting that C_4H_8O saturates active sites even at these high concentrations of [C_4H_8O]. The activation barriers reported in Figure 3 of the main text were made under these conditions to ensure C_4H_8O-covered active sites at each [CH_3OH].

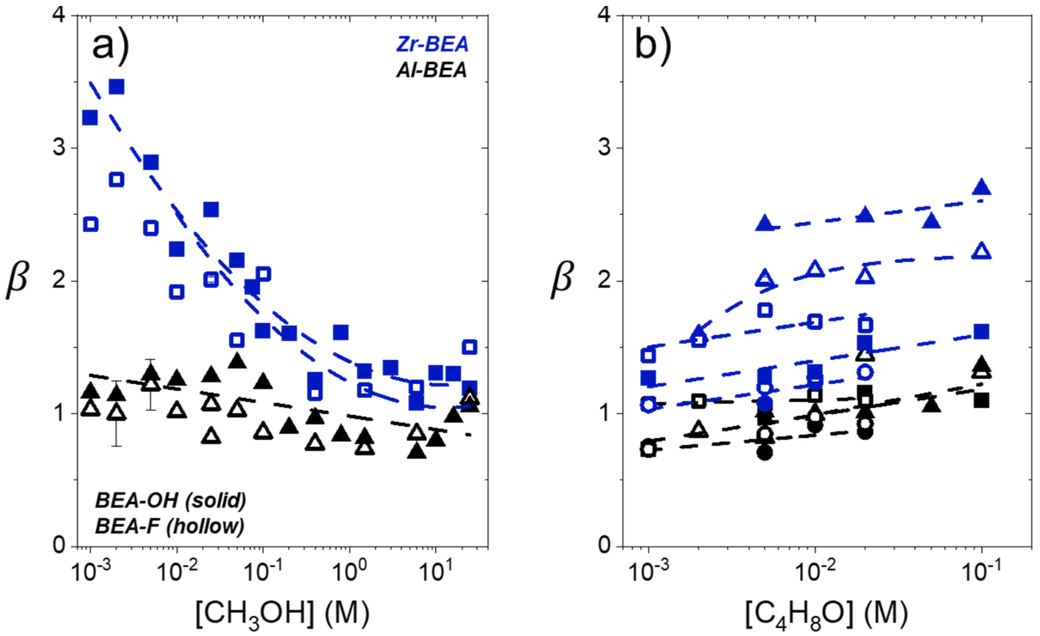

Figure S26. Values of β for C_4H_8O ring-opening with CH_3OH as a function of a) CH_3OH concentration (0.005 M C_4H_8O, CH_3CN solvent, 308 K), and b) C_4H_8O concentration at 0.01 M (triangles), 6 M (circles), or 24.7 M CH_3OH (squares) (CH_3CN, 308 K) over hydrophilic (solid) and hydrophilic (hollow) Al- (black) and Zr-BEA (blue).

Figure S26a shows β values for C_4H_8O ring-opening at a constant [C_4H_8O] (0.005 M), with regimes under which C_4H_8O or CH_3OH covers active sites. Figure S26b reveals the β values under several [CH_3OH], also under a regime where either C_4H_8O or CH_3OH covers active sites. Notably, Figure S26 shows very similar trends to Figure 7 in the main text, where greater [CH_3OH] leads to lower β values and greater [C_4H_8O] provides higher β values. The similarity among the trends demonstrates that the shift from C_4H_8O to CH_3OH-saturated active sites at high [CH3OH] in Figure S26 does not strongly affect trends in β, supporting that the differences in β in Figure 7 stem from changes in the intrapore solvent environment rather than any influence of the dominant surface intermediate.

S10. Measurements of Intrapore Solvent Composition

The liquid-phase concentrations of mixtures of CH_3OH and CH_3CN were quantified by GC before and after contact with M-BEA to determine the intrapore solvent composition. The ratio of the GC peak areas for each component was calculated for each sample before ($A_{CH_3OH:CH_3CN,initial}$) and after ($A_{CH_3OH:CH_3CN,final}$) adding the catalyst to the mixture. Then, the known initial mole fraction of CH_3OH ($x_{CH_3OH,bulk,initial}$) was used to calculate the final bulk solvent fractions:

$$x_{CH_3OH,bulk,final} = x_{CH_3OH,bulk,initial} * \frac{A_{CH_3OH:CH_3CN,final}}{A_{CH_3OH:CH_3CN,initial}} \tag{S18}$$

$$x_{CH_3CN,bulk,final} = 1 - x_{CH_3OH,bulk,final} \tag{S19}$$

The fraction of the initial solvent volume remaining in the bulk solvent after adsorption ($x_{bulk,final}$) was then calculated by estimating the fraction of solvent adsorbed from the bulk solvent ($V_{solvent,ads}$):

$$V_{solvent,ads} = m_{zeolite} * V_{pore,^*BEA} \tag{S20}$$

$$x_{bulk,final} = 1 - \left(\frac{V_{solvent,ads}}{V_{solvent,total}}\right) \tag{S21}$$

where $m_{zeolite}$ is the mass of M-BEA added to the solvent and $V_{pore,^*BEA}$ is the pore volume of *BEA, measured for a Ti-BEA material in a previous study by Ar physisorption[72] and assumed to be identical for all M-BEA here.

From there, the intrapore molar ratio ($\frac{n_{CH_3OH,pore}}{n_{CH_3CN,pore}}$) and CH_3OH fraction ($x_{CH_3OH,pore}$) can be calculated:

$$\frac{n_{CH_3OH,pore}}{n_{CH_3CN,pore}} = \frac{x_{CH_3OH,bulk,initial} - (x_{bulk,final} * x_{CH_3OH,bulk,final})}{x_{CH_3CN,bulk,initial} - (x_{bulk,final} * x_{CH_3CN,bulk,final})} \tag{S22}$$

$$x_{CH_3OH,pore} = \frac{\frac{n_{CH_3OH,pore}}{n_{CH_3CN,pore}}}{1 - \frac{n_{CH_3OH,pore}}{n_{CH_3CN,pore}}} \tag{S23}$$

Figure 4 in the main text reports $x_{CH_3OH,bulk}$ values as a function of the ratio of $x_{CH_3OH,pore}$ to $x_{CH_3OH,bulk}$, defined as χ in Equation 11 of the main text:

$$\chi = \frac{x_{CH_3OH,pore}}{x_{CH_3OH,bulk}} \tag{S24}$$

A full analysis of these solvent uptake measurements are included in Section 3.3 of the main text. Generally, Figure 4 demonstrates that β values decrease at greater $x_{CH_3OH,pore}$ for all M-BEA, providing evidence that the solvent structure surrounding active sites may strongly influence regioselectivities for C_4H_8O ring-opening through changes in $G_{2M1B}{}^{‡,\varepsilon}$ and $G_{1M2B}{}^{‡,\varepsilon}$. Of note, this result demonstrates that the choice of active metal not only influences G^0 values of reactive species through covalent interactions, but also can indirectly affect G^{ε}values by influencing the surrounding solvent structure. The choice of active metal leads to a span of β values even at nearly identical $x_{CH_3OH,pore}$, showing that differences in G^0 stemming from the properties of the active site contribute to differences in β.

S11. Isothermal Titration Calorimetry Thermograms and Heats per Epoxide Injection

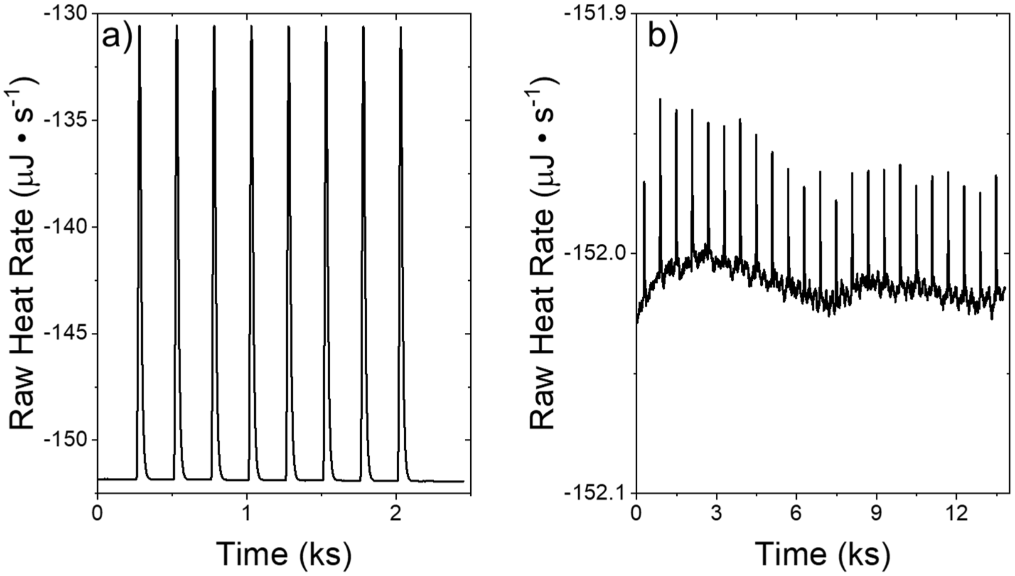

Figure S27. a) ITC thermogram from the electrical calibration of the NanoITC. b) ITC thermogram from the water-water adsorption to check the cleanliness of the ITC cell.

The plots in Figure S27 were obtained during the cleaning and calibration procedures for isothermal titration calorimetry (ITC), as discussed in Section 2.5 of the main text. Figure S27a shows the heat released from the sequential pulses during the electrical calibration. The instrument gave a calibration factor after the calibration was completed, for which values greater than 0.98 were assumed to be satisfactory. Figure S27b shows the heat released during the water-water injection, during which each 1 μL injection led to a peak with an area between -3 and +3 μJ.

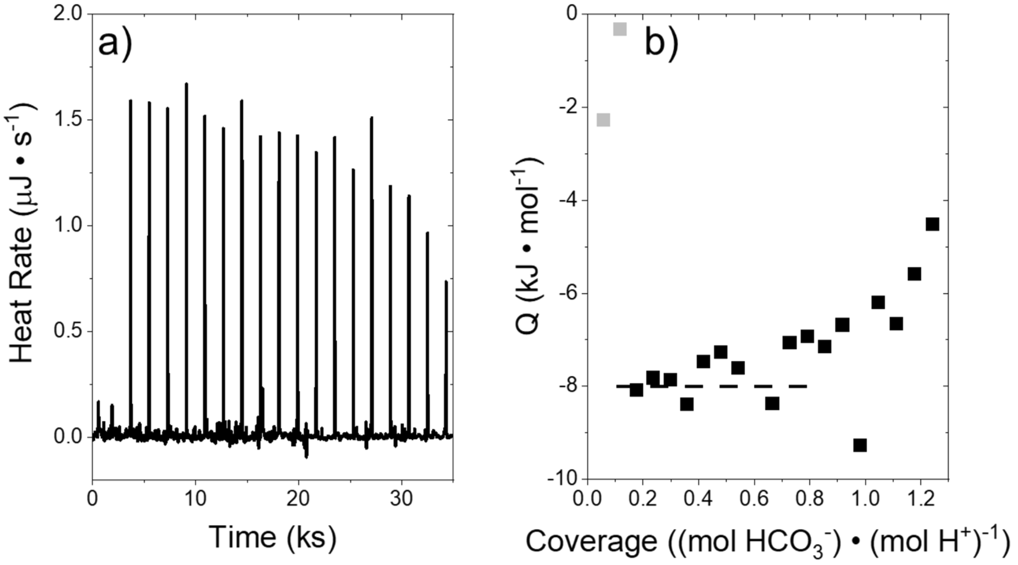

Figure S28. a) ITC thermogram from the acid-base titration of HNO_3 (0.0005 M in 0.1 M NaCl) with $NaHCO_3$ (0.0052 M in 0.1 M NaCl), b) the corresponding heats released as a function of bicarbonate ion coverage.

Figure S28 shows an acid-base titration of $NaHCO_3$ into HNO_3, which was used as a standard to verify the results obtained from the NanoITC. The adsorption enthalpy calculated from Figure S28b was -8.1 ± 1.2 kJ mol^{-1}, which agrees well with the reported enthalpy of -9.1 kJ mol^{-1}.[73-74]

ΔH_{ads} values for all ITC experiments shown here were calculated by averaging the non-opaqued points in the heat vs. coverage plot, from a regime in which the integrated heat remains approximately constant (see Figure 6 in main text). The ΔH_{ads} values are illustrated by the dashed line intersecting these points. The uncertainties were calculated from a single standard deviation among the points used to calculate ΔH_{ads}.

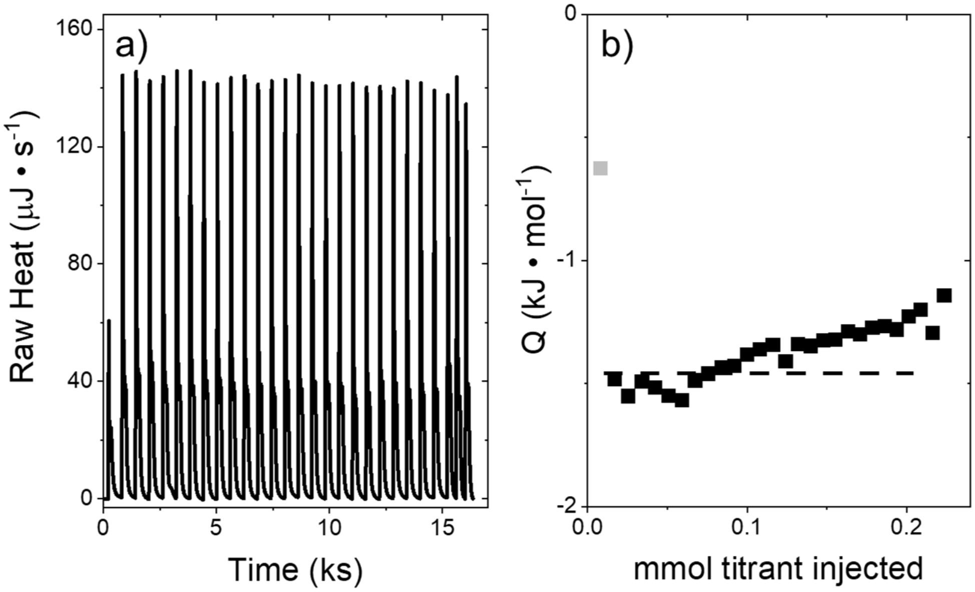

Figure S29. a) ITC thermogram from the titration of CH_3OH with neat 1M2B (8.7 M 1M2B, 308 K, 1 μL per injection), b) the corresponding heats released as a function of titrant injected.

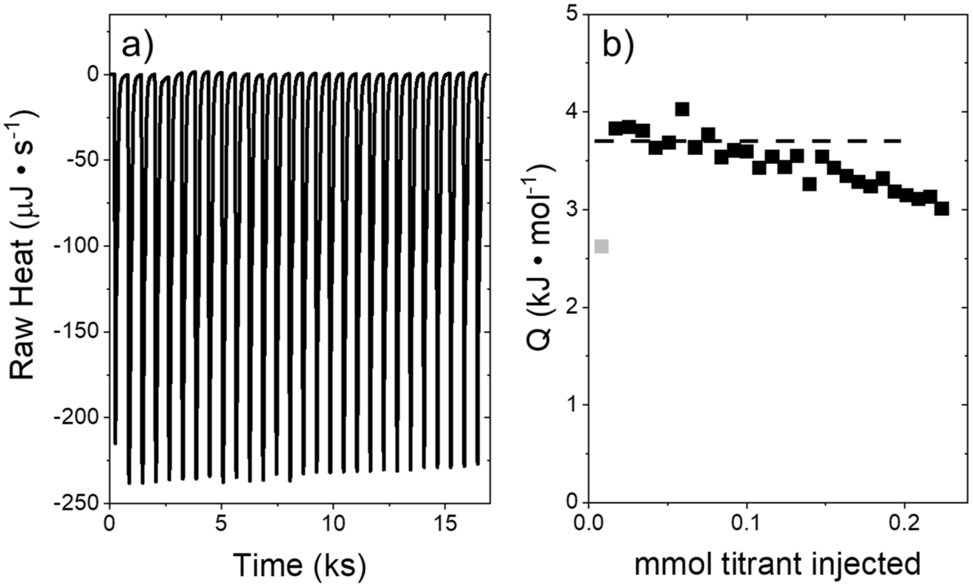

Figure S30. a) ITC thermogram from the titration of CH_3CN with neat 1M2B (8.7 M 1M2B, 308 K, 1 μL per injection), b) the corresponding heats released as a function of titrant injected.

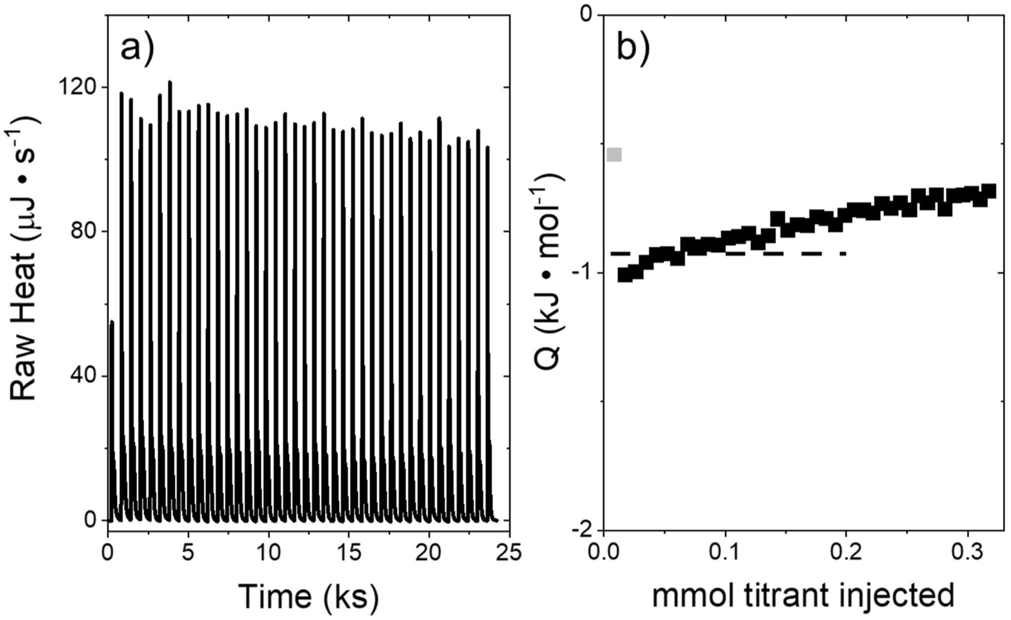

Figure S31. a) ITC thermogram from the titration of CH_3OH with neat 2M1B (8.9 M 2M1B, 308 K, 1 μL per injection), b) the corresponding heats released as a function of titrant injected.

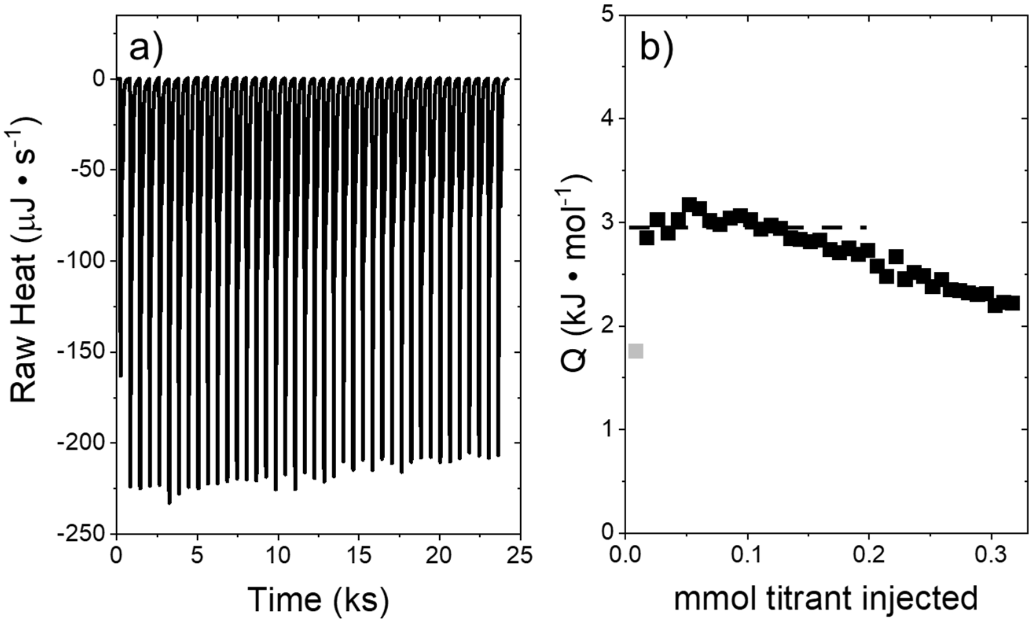

Figure S32. a) ITC thermogram from the titration of CH_3CN with neat 2M1B (8.9 M 2M1B, 308 K, 1 μL per injection), b) the corresponding heats released as a function of titrant injected.

Figures S29 to S32 report the heat changes from the injection of the neat ring-opening products into CH_3OH or CH_3CN. Each product adsorbs exothermically into CH_3OH and endothermically into CH_3CN. 1M2B shows a more exothermic enthalpy of mixing into CH_3OH than 2M1B, but 2M1B shows a less endothermic enthalpy of mixing into CH_3CN than 1M2B. While these measurements provide insight into the stability of the products in CH_3OH and CH_3CN-rich solvents, they do not correlate with the trends of β values or $\Delta H_{ads,prod.}$ values into each solvent, indicating that the active metal and solvating environment within the pores of M-BEA govern these differences.

Figure S33 presents an ITC thermogram and heat release plot from the adsorption of C_4H_8O into Al-BEA at high coverages, to test if lateral interactions between nearby active sites play a role in the adsorption enthalpy of C_4H_8O. The integrated heat begins to decrease sharply near an epoxide coverage of 1, which supports that the Brønsted acid sites bind 1 epoxide molecule on average. Furthermore, the integrated heats change negligibly as the epoxide coverage approaches 1, which suggests that lateral interactions do not play a strong role between proximal active sites. The absence of these effects in the material with Al-BEA, which shows the highest density of active sites (Si:M ~70), also indicates that lateral interactions will not play a role in Ti-, Sn-, or Zr-BEA (Si:M > 90).

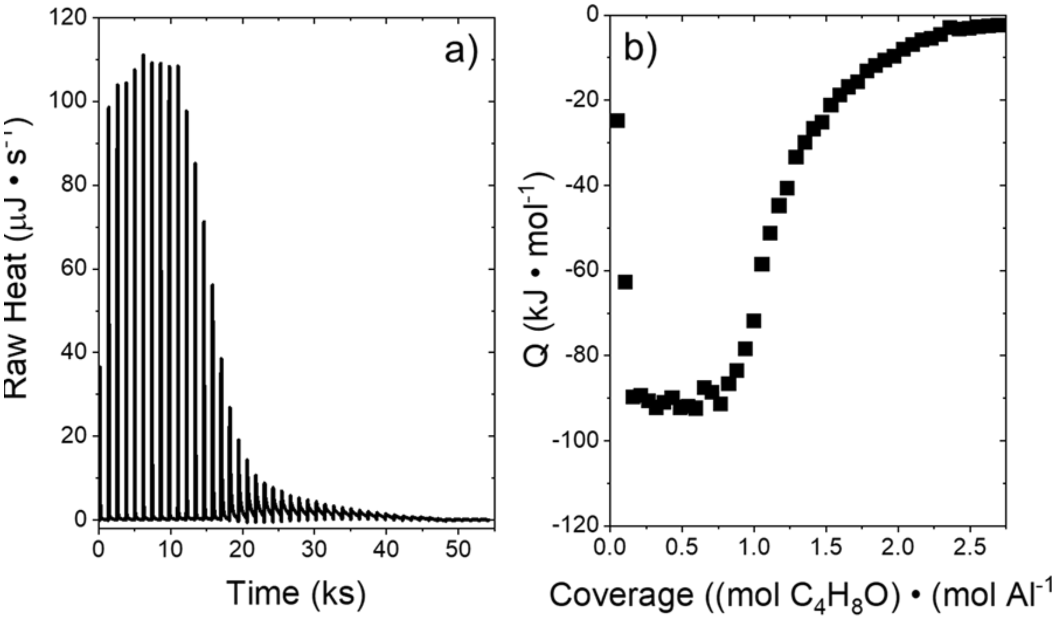

Figure S33. a) ITC thermogram from the titration of Al-BEA-OH with C_4H_8O (0.05 M C_4H_8O in CH_3OH, 308 K, 1 μL per injection), b) the corresponding heats released as a function of titrant injected.

The ITC thermogram and heat release plots for all ITC experiments used to calculate the adsorption enthalpies of C_4H_8O into M-BEA, which were run at low coverages as compared to Figure S33, are shown below. ITC measurements for CH_3OH adsorption into M-BEA are also shown below, although these were performed at higher titrant concentrations (and thus higher coverages) because CH_3OH adsorbs weakly and higher concentrations are needed to observe sufficiently large peaks in the thermogram.

As discussed in Section 3.3, early injections often show lower heat released than expected due to the evaporation of liquid from the syringe needle. Although less common, some of the experiments below show outlier points at later injections, which may result from an inconsistent volume or epoxide concentration in those injections.

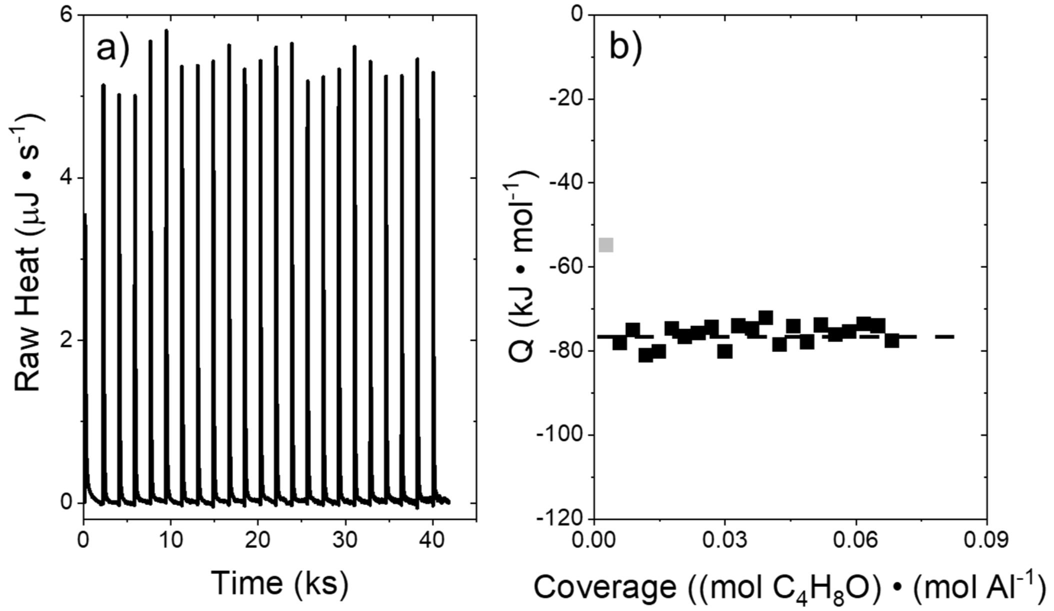

Figure S34. a) ITC thermogram from the titration of Al-BEA-OH with C_4H_8O (0.005 M C_4H_8O in CH_3OH, 308 K, 1 μL per injection), b) the corresponding heats released as a function of titrant injected.

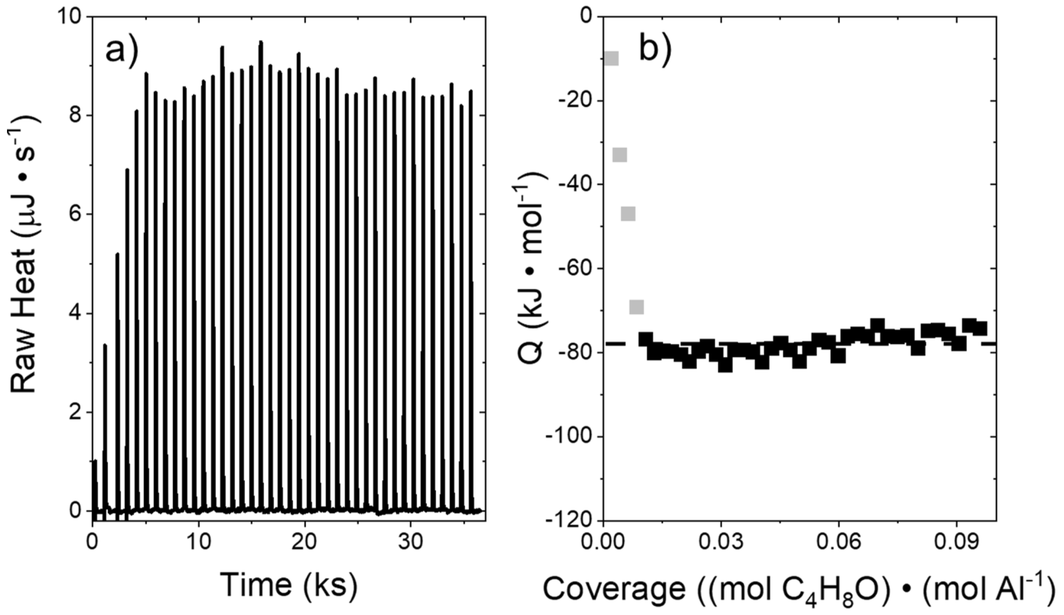

Figure S35. a) ITC thermogram from the titration of Al-BEA-OH with C_4H_8O (0.005 M C_4H_8O in CH_3OH, 308 K, 1 μL per injection), b) the corresponding heats released as a function of titrant injected. This point was repeated to ensure accuracy and reproducibility of measurements.

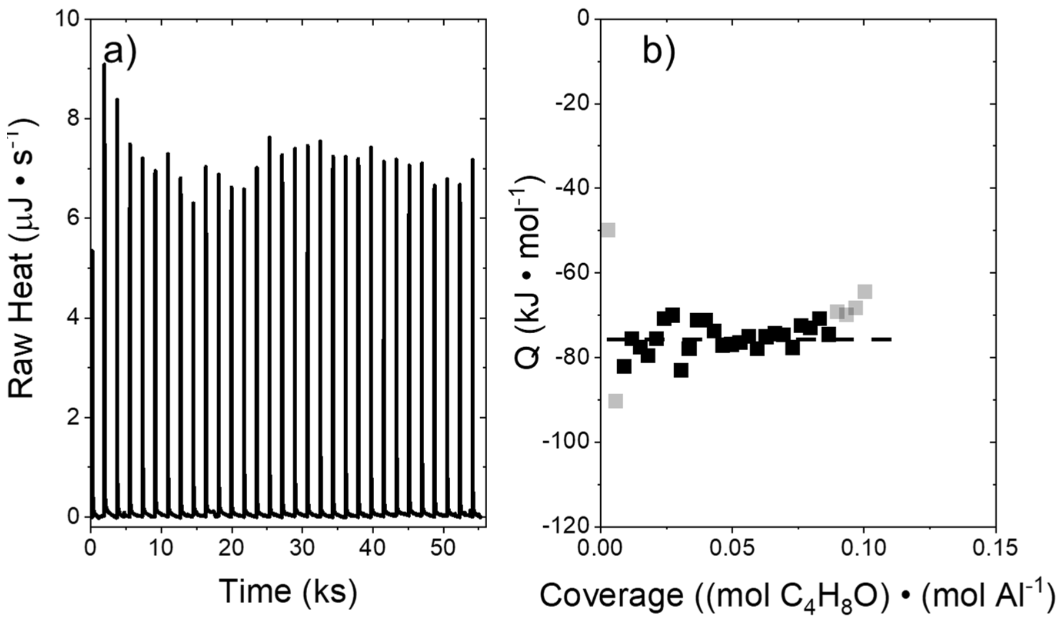

Figure S36. a) ITC thermogram from the titration of Al-BEA-F with C_4H_8O (0.005 M C_4H_8O in CH_3OH, 308 K, 1 μL per injection), b) the corresponding heats released as a function of titrant injected.

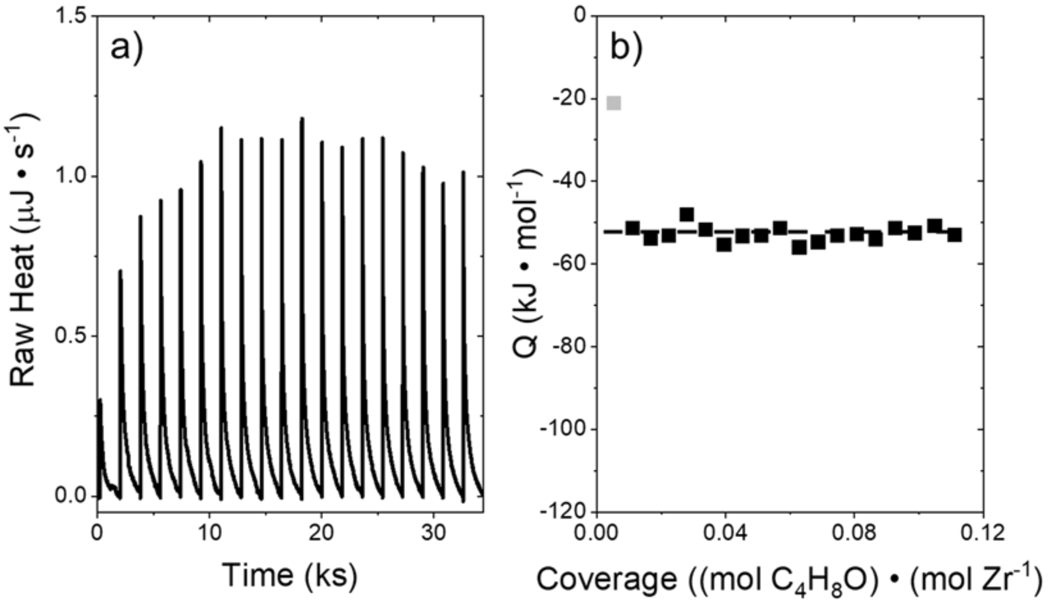

Figure S37. a) ITC thermogram from the titration of Zr-BEA-OH with C_4H_8O (0.005 M C_4H_8O in CH_3OH, 308 K, 1 μL per injection), b) the corresponding heats released as a function of titrant injected.

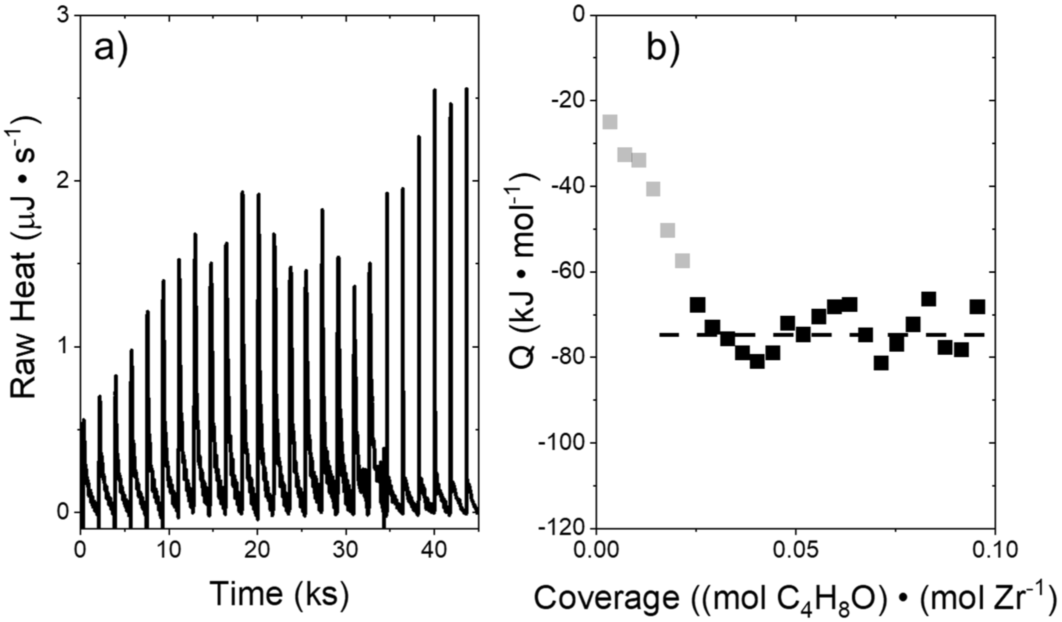

Figure S38. a) ITC thermogram from the titration of Zr-BEA-F with C_4H_8O (0.005 M C_4H_8O in CH_3OH, 308 K, 1 μL per injection), b) the corresponding heats released as a function of titrant injected.

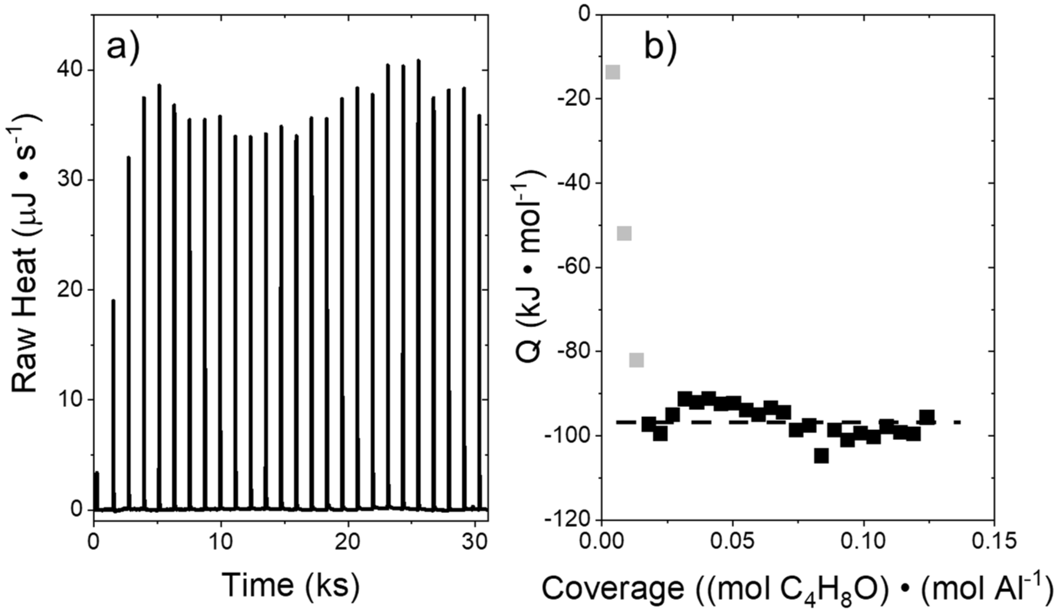

Figure S39. a) ITC thermogram from the titration of Al-BEA-OH with C_4H_8O (0.005 M C_4H_8O in CH_3CN, 308 K, 1 μL per injection), b) the corresponding heats released as a function of titrant injected.

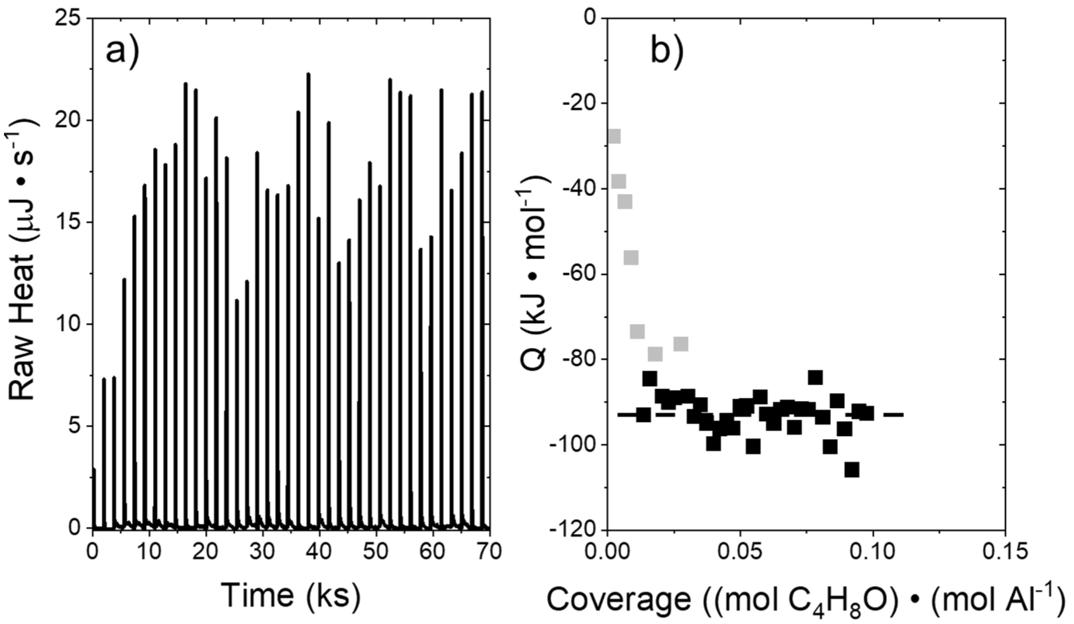

Figure S40. a) ITC thermogram from the titration of Al-BEA-F with C_4H_8O (0.005 M C_4H_8O in CH_3CN, 308 K, 1 μL per injection), b) the corresponding heats released as a function of titrant injected.

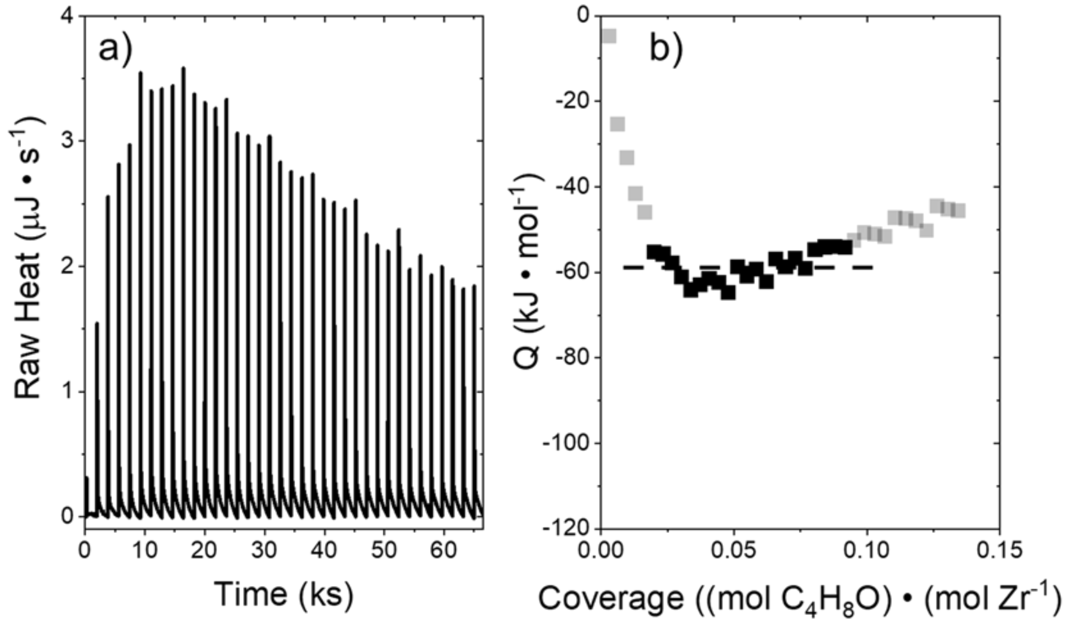

Figure S41. a) ITC thermogram from the titration of Zr-BEA-OH with C_4H_8O (0.005 M C_4H_8O in CH_3CN, 308 K, 1 μL per injection), b) the corresponding heats released as a function of titrant injected.

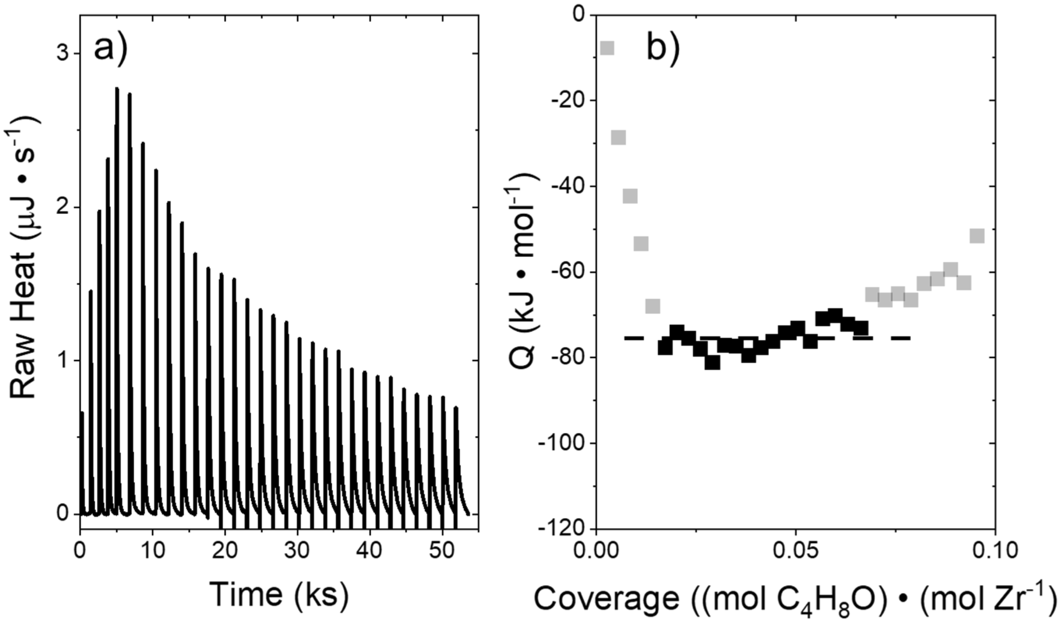

Figure S42. a) ITC thermogram from the titration of Zr-BEA-F with C_4H_8O (0.005 M C_4H_8O in CH_3CN, 308 K, 1 μL per injection), b) the corresponding heats released as a function of titrant injected.

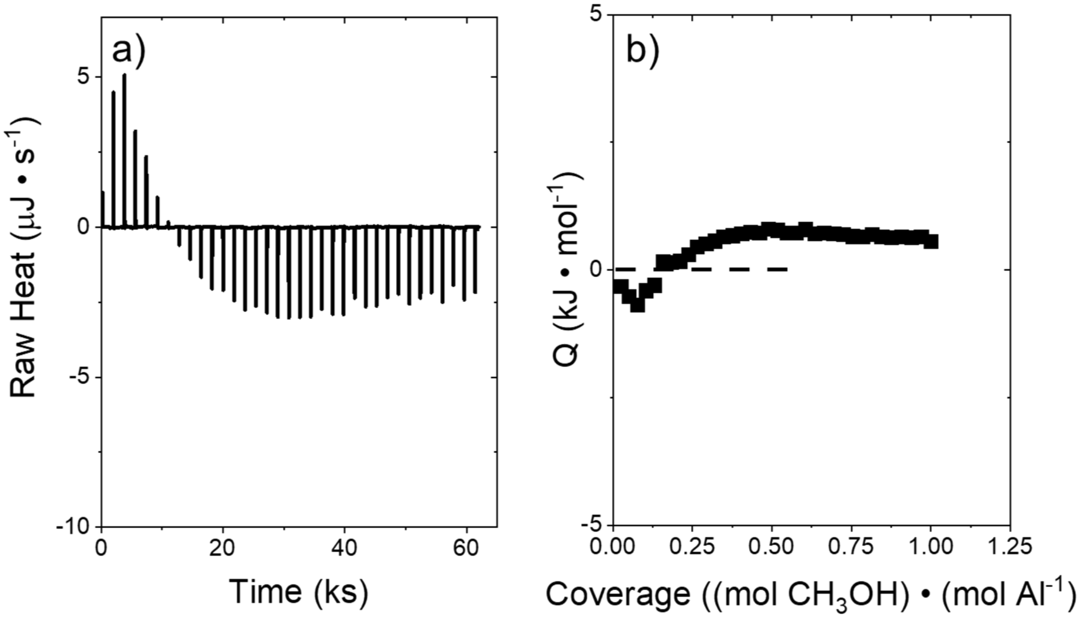

Figure S43. a) ITC thermogram from the titration of Al-BEA-OH with CH_3OH (0.1 M CH_3OH in CH_3CN, 308 K, 1 μL per injection), b) the corresponding heats released as a function of titrant injected.

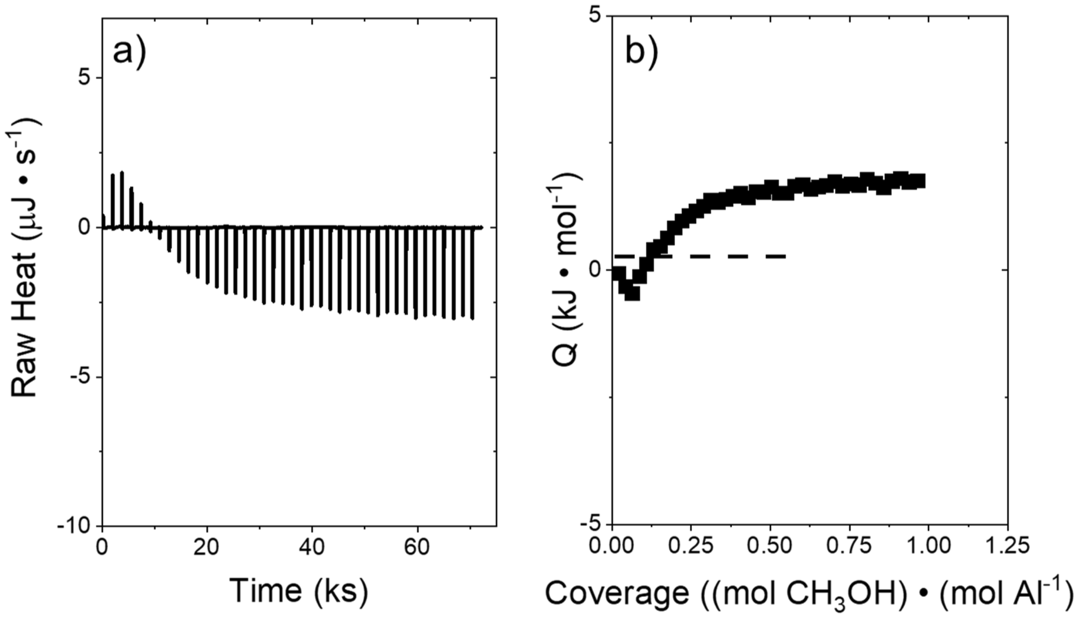

Figure S44. a) ITC thermogram from the titration of Al-BEA-F with CH_3OH (0.1 M CH_3OH in CH_3CN, 308 K, 1 μL per injection), b) the corresponding heats released as a function of titrant injected.

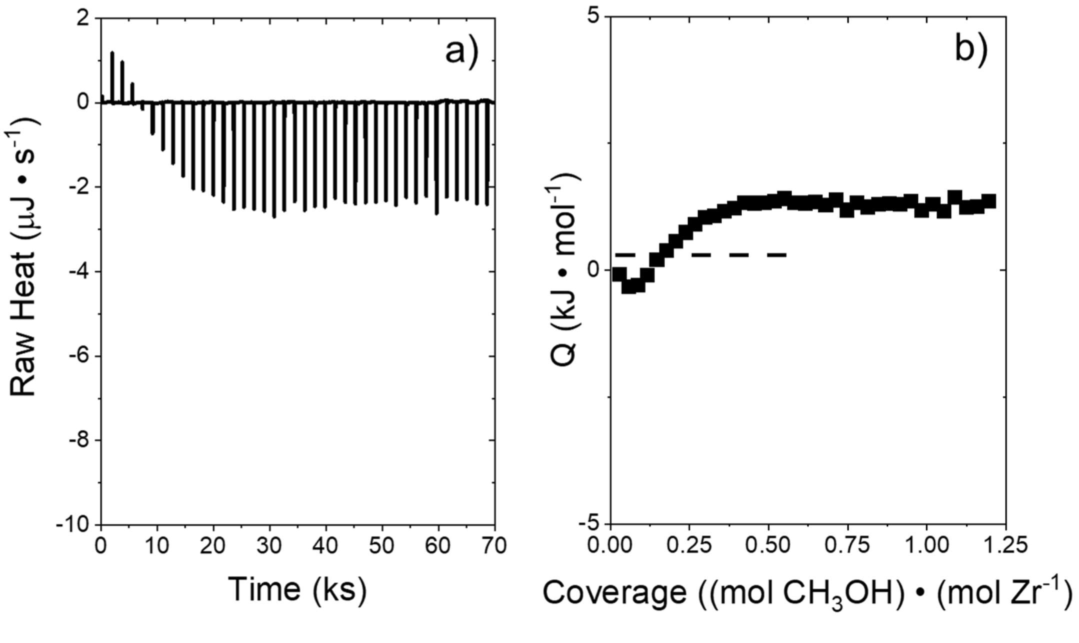

Figure S45. a) ITC thermogram from the titration of Zr-BEA-OH with CH_3OH (0.1 M CH_3OH in CH_3CN, 308 K, 1 μL per injection), b) the corresponding heats released as a function of titrant injected.

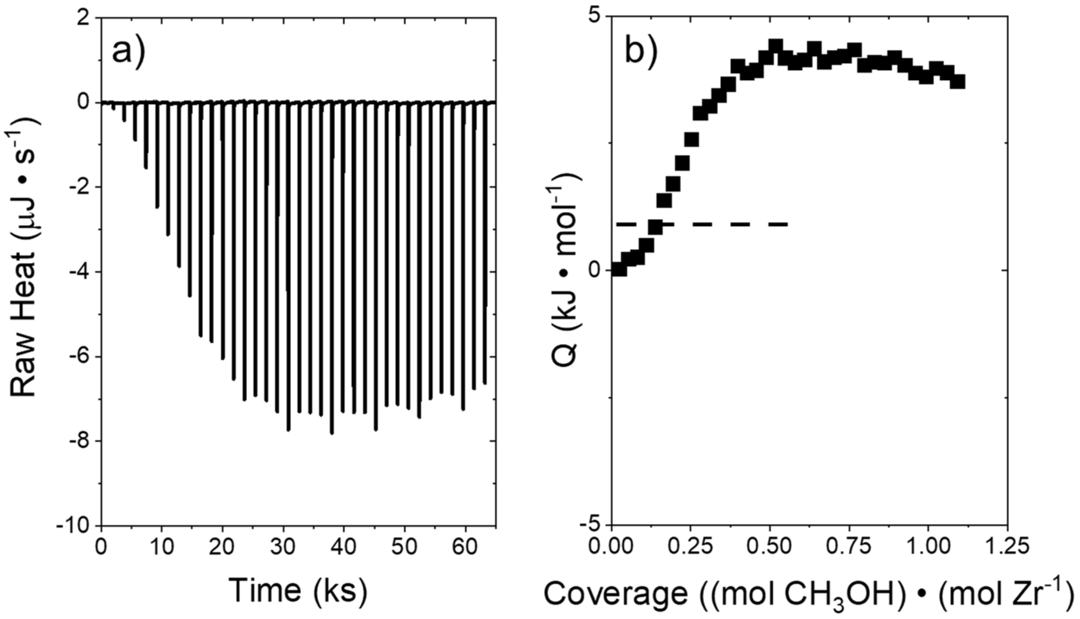

Figure S46. a) ITC thermogram from the titration of Zr-BEA-F with CH_3OH (0.1 M CH_3OH in CH_3CN, 308 K, 1 μL per injection), b) the corresponding heats released as a function of titrant injected.

S12. Accounting for Reaction Enthalpy Contribution in $\Delta H^{\ddagger}$ - $\Delta H_{ads,C_4H_8O}$ Correlation

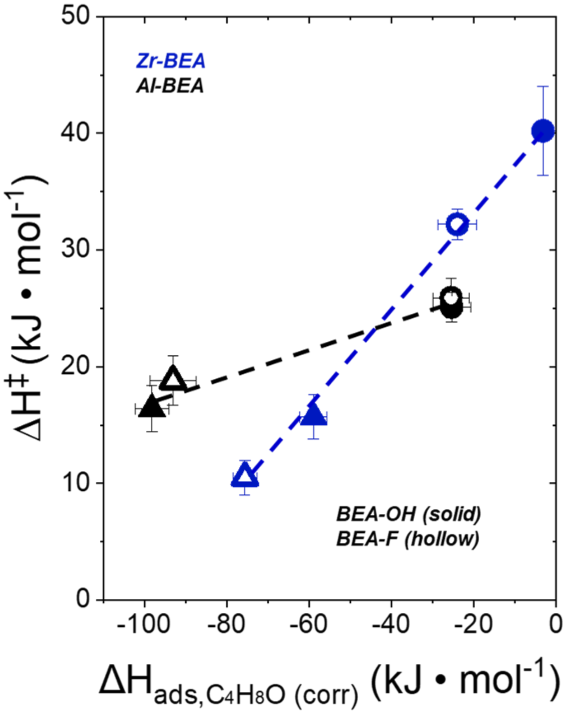

Figure S47. $\Delta H^{\ddagger}$ values for C_4H_8O ring-opening (from Figure 3, 0.025 M CH_3OH in CH_3CN (triangles) or 24.7 M CH_3OH (circles)) as a function of the reaction-corrected adsorption enthalpy of C_4H_8O ($\Delta H_{ads,C_4H_8O(corr)}$) over hydrophilic (solid) and hydrophilic (hollow) Al- (black) and Zr-BEA (blue) materials. Assumed contribution from enthalpy of reaction between C_4H_8O and CH_3OH are subtracted from $\Delta H_{ads,C_4H_8O}$ (reported in Figure 7) to get $\Delta H_{ads,C_4H_8O\,(corr)}$.

The enthalpy of reaction was estimated from the gas-phase formation enthalpies for the products and reactants:

$$\Delta H_{rxn} = \Delta H_{f,C_5H_{12}O_2} - \Delta H_{f,C_4H_8O} - \Delta H_{f,CH_3OH} \tag{S25}$$

The formation enthalpies of C_4H_8O (-185.1 kJ mol^{-1}) and CH_3OH (-201.3 kJ mol^{-1}) are tabulated[75] but the values for the products are not ($C_5H_{12}O_2$ – 1M2B and 2M1B). These values were estimated using the Joback group-contribution method,[76] which yielded equal values for both products (-436.3 kJ mol^{-1}). These values give an estimated ΔH_{rxn} value of -49.9 kJ mol^{-1}.

The corrected adsorption enthalpy in Figure S47 assumes that all epoxide that adsorbs at 24.7 M CH_3OH reacts over the course of the injection period (1800 – 2400 s), and subtracts out a contribution equivalent to the estimated ΔH_{rxn} value:

$$\Delta H_{ads,C_4H_8O(corr)} = \Delta H_{ads,C_4H_8O} - \Delta H_{rxn} \tag{S26}$$

The correction shifts all adsorption enthalpies to more endothermic values. All points for Zr-BEA fall on a line after this correction, in contrast to the discontinuity in Figure 7 of the main text. The points for Al-BEA still scale linearly as in Figure 7, but with a much shallower slope.

The reaction-corrected adsorption enthalpy can be expressed as follows:

$$\Delta H_{ads,C_4H_8O(corr)} = H_{C_4H_8O}{}^{*} - H_{C_4H_8O} - H_{*} \tag{S27}$$

where H_* represents the enthalpy of the Lewis and Brønsted acid sites within Zr- and Al-BEA, respectively. The solvation of the proton by CH_3OH may decrease H_* values, leading to less exothermic H_* values and the shallower slope for the Al-BEA correlation compared to Zr-BEA.

While the reaction-corrected adsorption enthalpies do show linear correlations, they rely on the assumption that all epoxide molecules react within the span of each injection into the ITC cell. The rate and extent of reaction cannot be monitored over time in the ITC, making it impossible to test the validity of the assumption. However, the conclusions discussed in Section 3.4 irrespective of the extent of reaction during C_4H_8O adsorption into CH_3OH. The correlations between $\Delta H^{\ddagger}$ and $\Delta H_{ads,C_4H_8O}$ at a given solvent composition demonstrate that the adsorption of C_4H_8O and the formation of the C_4H_8O ring-opening transition state reorganize the surrounding solvent structure in similar ways, which leads to differences in both the kinetics and thermodynamics of epoxide ring-opening.

S13. Adsorption Enthalpies of C_4H_8O and CH_3OH to M-BEA in CH_3CN

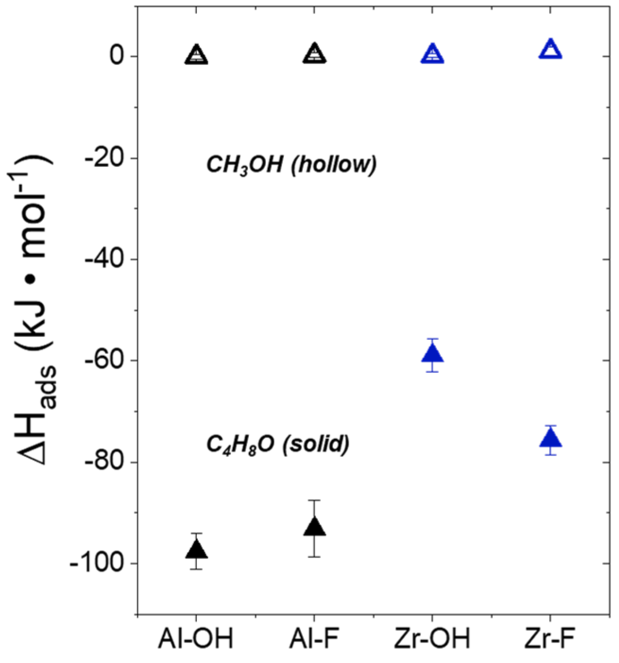

Figure S48. Adsorption enthalpies of C_4H_8O (solid points, 0.005 M C_4H_8O, 308 K) and CH_3OH (hollow points, 0.1 M CH_3OH, 308 K) as a function of active metal in CH_3CN over Zr- (blue) and Al-BEA (black) materials.

The adsorption enthalpies of C_4H_8O and CH_3OH shown in Figure S48 were obtained with isothermal titration calorimetry (see Section S11 for thermograms). For all M-BEA, C_4H_8O shows an adsorption enthalpy at least 50 kJ mol^{-1} more exothermic than CH_3OH. These values suggest that C_4H_8O adsorbs more readily to the active sites in M-BEA and likely explains why CH_3OH only saturates active sites under large excesses of CH_3OH ([CH_3OH]:[C_4H_8O] > 500, see Section 3.1 in the main text).

Figure S49 displays the heat released from CH_3OH adsorption over M-BEA as a function of CH_3OH coverage. Notably, Zr-BEA-OH and each Al-BEA material show similar trends with CH_3OH coverage. CH_3OH adsorbs exothermically at a CH_3OH coverage below 0.25, after which the adsorption heat gradually increases to reach a nearly constant and endothermic value. In contrast, Zr-BEA-F shows an endothermic heat at all CH_3OH coverages, with the most endothermic heats of all M-BEA at high coverages. These trends suggest that CH_3OH adsorbs more favorably over Zr-BEA-OH and each Al-BEA than Zr-BEA-F, aligning with the trends of CH_3OH uptake from binary mixtures with CH_3CN (Figure 4). Furthermore, the curves of Al-BEA-OH and Al-BEA-F show more similar trends than Zr-BEA-OH and Zr-BEA-F, providing further support that the intrapore environment within Brønsted acid zeolite materials depends less strongly on $(SiOH)_x$ density than Lewis acid zeolites.

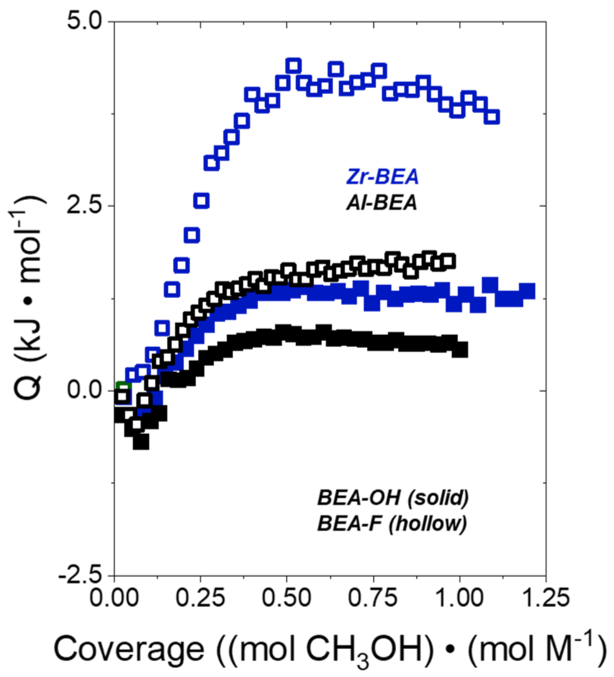

Figure S49. Adsorption enthalpies of C_4H_8O (solid points, 0.005 M C_4H_8O, 308 K) and CH_3OH (hollow points, 0.1 M CH_3OH, 308 K) as a function of active metal in CH_3CN over Zr- (blue) and Al-BEA (black) materials.

References

1. Newsam, J. M.; Treacy, M. M. J.; Koetsier, W. T.; Gruyter, C. B. D., Structural characterization of zeolite beta. *Proceedings of the Royal Society of London. A. Mathematical and Physical Sciences* **1988,** *420* (1859), 375-405.
2. Camblor, M. A.; Corma, A.; Valencia, S., Synthesis in fluoride media and characterisation of aluminosilicate zeolite beta. *Journal of Materials Chemistry* **1998,** *8* (9), 2137-2145.
3. Vega-Vila, J. C.; Harris, J. W.; Gounder, R., Controlled insertion of tin atoms into zeolite framework vacancies and consequences for glucose isomerization catalysis. *J Catal* **2016,** *344*, 108-120.
4. Filatova, E. O.; Konashuk, A. S., Interpretation of the changing the band gap of Al2O3 depending on its crystalline form: connection with different local symmetries. *The Journal of Physical Chemistry C* **2015,** *119* (35), 20755-20761.
5. Mo, S. D.; Xu, Y. N.; Ching, W. Y., Electronic and Structural Properties of Bulk γ-Al2O3. *Journal of the American Ceramic Society* **1997,** *80* (5), 1193-1197.
6. Mihailova, B.; Valtchev, V.; Mintova, S.; Faust, A. C.; Petkov, N.; Bein, T., Interlayer stacking disorder in zeolite beta family: a Raman spectroscopic study. *Physical Chemistry Chemical Physics* **2005,** *7* (14), 2756.
7. Tosheva, L.; Mihailova, B.; Valtchev, V.; Sterte, J., Zeolite beta spheres. *Microporous and Mesoporous Materials* **2001,** *48* (1-3), 31-37.
8. Majano, G.; Mintova, S.; Ovsitser, O.; Mihailova, B.; Bein, T., Zeolite Beta nanosized assemblies. *Microporous and Mesoporous Materials* **2005,** *80* (1-3), 227-235.
9. Inagaki, S.; Nakatsuyama, K.; Saka, Y.; Kikuchi, E.; Kohara, S.; Matsukata, M., Elucidation of Medium-Range Structure in a Dry Gel-Forming *BEA-Type Zeolite. *The Journal of Physical Chemistry C* **2007,** *111* (28), 10285-10293.
10. Yu, Y.; Xiong, G.; Li, C.; Xiao, F.-S., Characterization of aluminosilicate zeolites by UV Raman spectroscopy. *Microporous and Mesoporous Materials* **2001,** *46* (1), 23-34.
11. Knops-Gerrits, P.-P.; De Vos, D. E.; Feijen, E. J.; Jacobs, P. A., Raman spectroscopy on zeolites. *Microporous Materials* **1997,** *8* (1-2), 3-17.
12. Aminzadeh, A., Excitation frequency dependence and fluorescence in the Raman spectra of Al2O3. *Applied spectroscopy* **1997,** *51* (6), 817-819.
13. Porto, S.; Krishnan, R., Raman effect of corundum. *The Journal of Chemical Physics* **1967,** *47* (3), 1009-1012.
14. Aminzadeh, A.; Sarikhani-Fard, H., Raman spectroscopic study of Ni/Al2O3 catalyst. *Spectrochimica Acta Part A: Molecular and Biomolecular Spectroscopy* **1999,** *55* (7-8), 1421-1425.
15. Purohit, R.; Saha, S.; Tyagi, A., Combustion synthesis of nanocrystalline ZrO2 powder: XRD, Raman spectroscopy and TEM studies. *Materials Science and Engineering: B* **2006,** *130* (1-3), 57-60.
16. Yi, M.; Zhang, Y.; Xu, J.; Deng, D.; Mao, Z.; Meng, X.; Shi, X.; Zhao, B., Surface-Enhanced Raman Scattering Activity of ZrO2 Nanoparticles: Effect of Tetragonal and Monoclinic Phases. *Nanomaterials* **2021,** *11* (09), 2162.
17. Kim, D. J.; Jung, H. J.; Yang, I. S., Raman spectroscopy of tetragonal zirconia solid solutions. *Journal of the American Ceramic Society* **1993,** *76* (8), 2106-2108.
18. Scarano, D.; Zecchina, A.; Bordiga, S.; Geobaldo, F.; Spoto, G.; Petrini, G.; Leofanti, G.; Padovan, M.; Tozzola, G., Fourier-transform infrared and Raman spectra of pure and Al-, B-, Ti- and Fe-substituted silicalites: stretching-mode region. *Journal of the Chemical Society, Faraday Transactions* **1993,** *89* (22), 4123.
19. Blasco, T.; Camblor, M. A.; Corma, A.; Esteve, P.; Guil, J. M.; Martinez, A.; Perdigon-Melon, J. A.; Valencia, S., Direct synthesis and characterization of hydrophobic aluminum-free Ti-beta zeolite. *Journal of Physical Chemistry B* **1998,** *102,* 75-88.
20. Mihailova, B.; Valtchev, V.; Mintova, S.; Faust, A.-C.; Petkov, N.; Bein, T., Interlayer stacking disorder in zeolite beta family: a Raman spectroscopic study. *Physical chemistry chemical physics* **2005,** *7* (14), 2756-2763.

21. Ricchiardi, G.; Damin, A.; Bordiga, S.; Lamberti, C.; Spanò, G.; Rivetti, F.; Zecchina, A., Vibrational Structure of Titanium Silicate Catalysts. A Spectroscopic and Theoretical Study. *Journal of the American Chemical Society* **2001,** *123* (46), 11409-11419.
22. Bordiga, S.; Damin, A.; Bonino, F.; Ricchiardi, G.; Zecchina, A.; Tagliapietra, R.; Lamberti, C., Resonance Raman effects in TS-1: the structure of Ti(iv) species and reactivity towards H2O, NH3 and H2O2: an in situ study. *Physical Chemistry Chemical Physics* **2003,** *5* (20), 4390.
23. Signorile, M.; Crocellà, V.; Damin, A.; Rossi, B.; Lamberti, C.; Bonino, F.; Bordiga, S., Effect of Ti Speciation on Catalytic Performance of TS-1 in the Hydrogen Peroxide to Propylene Oxide Reaction. *The Journal of Physical Chemistry C* **2018,** *122* (16), 9021-9034.
24. Li, C.; Xiong, G.; Liu, J.; Ying, P.; Xin, Q.; Feng, Z., Identifying Framework Titanium in TS-1 Zeolite by UV Resonance Raman Spectroscopy. *The Journal of Physical Chemistry B* **2001,** *105* (15), 2993-2997.
25. Sushkevich, V. L.; Vimont, A.; Travert, A.; Ivanova, I. I., Spectroscopic evidence for open and closed Lewis acid sites in ZrBEA zeolites. *The Journal of Physical Chemistry C* **2015,** *119* (31), 17633-17639.
26. Kurmach, M. M.; Larina, O. V.; Kyriienko, P. I.; Yaremov, P. S.; Trachevsky, V. V.; Shvets, O. V.; Soloviev, S. O., Hierarchical Zr-MTW Zeolites Doped with Copper as Catalysts of Ethanol Conversion into 1, 3-Butadiene. *ChemistrySelect* **2018,** *3* (29), 8539-8546.
27. Wichterlová, B.; Tvarůžková, Z.; Sobalık, Z.; Sarv, P., Determination and properties of acid sites in H-ferrierite: A comparison of ferrierite and MFI structures. *Microporous and mesoporous materials* **1998,** *24* (4-6), 223-233.
28. Penzien, J.; Abraham, A.; van Bokhoven, J. A.; Jentys, A.; Müller, T. E.; Sievers, C.; Lercher, J. A., Generation and characterization of well-defined Zn2+ Lewis acid sites in ion exchanged zeolite BEA. *The Journal of Physical Chemistry B* **2004,** *108* (13), 4116-4126.
29. Courtney, T. D.; Chang, C.-C.; Gorte, R. J.; Lobo, R. F.; Fan, W.; Nikolakis, V., Effect of water treatment on Sn-BEA zeolite: Origin of 960 cm− 1 FTIR peak. *Microporous and Mesoporous Materials* **2015,** *210*, 69-76.
30. Sushkevich, V. L.; Kots, P. A.; Kolyagin, Y. G.; Yakimov, A. V.; Marikutsa, A. V.; Ivanova, I. I., Origin of water-induced Brønsted acid sites in Sn-BEA zeolites. *The Journal of Physical Chemistry C* **2019,** *123* (9), 5540-5548.
31. Montejo-Valencia, B. D.; Salcedo-Pérez, J. L.; Curet-Arana, M. C., DFT study of closed and open sites of BEA, FAU, MFI, and BEC zeolites substituted with tin and titanium. *The Journal of Physical Chemistry C* **2016,** *120* (4), 2176-2186.
32. Bukowski, B. C.; Bates, J. S.; Gounder, R.; Greeley, J., First principles, microkinetic, and experimental analysis of Lewis acid site speciation during ethanol dehydration on Sn-Beta zeolites. *J Catal* **2018,** *365*, 261-276.
33. Bukowski, B. C.; Greeley, J., Scaling relationships for molecular adsorption and dissociation in Lewis acid zeolites. *The Journal of Physical Chemistry C* **2016,** *120* (12), 6714-6722.
34. Pelmenschikov, A.; Van Santen, R.; Janchen, J.; Meijer, E., Acetonitrile-d3 as a probe of Lewis and Broensted acidity of zeolites. *The Journal of Physical Chemistry* **1993,** *97* (42), 11071-11074.
35. Ward, J. W., A spectroscopic study of the surface of zeolite Y: the adsorption of pyridine. *Journal of Colloid and Interface Science* **1968,** *28* (2), 269-278.
36. Cannings, F. R., Acidic sites on mordenite: an infrared study of adsorbed pyridine. *The Journal of Physical Chemistry* **1968,** *72* (13), 4691-4693.
37. Kataoka, T.; Dumesic, J., Acidity of unsupported and silica-supported vanadia, molybdena, and titania as studied by pyridine adsorption. *J Catal* **1988,** *112* (1), 66-79.
38. Parry, E., An infrared study of pyridine adsorbed on acidic solids. Characterization of surface acidity. *J Catal* **1963,** *2* (5), 371-379.
39. Lefrancois, M.; Malbois, G., The nature of the acidic sites on mordenite: Characterization of adsorbed pyridine and water by infrared study. *J Catal* **1971,** *20* (3), 350-358.
40. Barzetti, T.; Selli, E.; Moscotti, D.; Forni, L., Pyridine and ammonia as probes for FTIR analysis of solid acid catalysts. *Journal of the Chemical Society, Faraday Transactions* **1996,** *92* (8), 1401-1407.

41. Sushkevich, V. L.; Ivanova, I. I.; Yakimov, A. V., Revisiting acidity of SnBEA catalysts by combined application of FTIR spectroscopy of different probe molecules. *The Journal of Physical Chemistry C* **2017,** *121* (21), 11437-11447.
42. Palomino, G. T.; Pascual, J. J. C.; Delgado, M. R.; Parra, J. B.; Areán, C. O., FT-IR studies on the acidity of gallium-substituted mesoporous MCM-41 silica. *Materials chemistry and physics* **2004,** *85* (1), 145-150.
43. Murphy, B. M.; Letterio, M. P.; Xu, B., Selectivity Control in the Catalytic Dehydration of Methyl Lactate: The Effect of Pyridine. *ACS Catalysis* **2016,** *6* (8), 5117-5131.
44. Tang, B.; Dai, W.; Sun, X.; Wu, G.; Guan, N.; Hunger, M.; Li, L., Mesoporous Zr-Beta zeolites prepared by a post-synthetic strategy as a robust Lewis acid catalyst for the ring-opening aminolysis of epoxides. *Green Chemistry* **2015,** *17* (3), 1744-1755.
45. Zhang, H.; Quek, Z. J.; Jaenicke, S.; Chuah, G.-K., Hydrophobicity and co-solvent effects on Meerwein-Ponndorf-Verley reduction/dehydration cascade reactions over Zr-zeolite catalysts. *J Catal* **2021,** *400*, 50-61.
46. Zhao, Z.; Xu, S.; Hu, M. Y.; Bao, X.; Peden, C. H.; Hu, J., Investigation of aluminum site changes of dehydrated zeolite H-beta during a rehydration process by high-field solid-state NMR. *The Journal of Physical Chemistry C* **2015,** *119* (3), 1410-1417.
47. van Bokhoven, J. A.; Van der Eerden, A.; Koningsberger, D., Flexible aluminium coordination of zeolites as function of temperature and water content, an in-situ method to determine aluminium coordinations. In *Studies in surface science and catalysis*, Elsevier: 2002; Vol. 142, pp 1885-1890.
48. Wouters, B.; Chen, T.-H.; Grobet, P., Reversible tetrahedral− octahedral framework aluminum transformation in zeolite Y. *Journal of the American Chemical Society* **1998,** *120* (44), 11419-11425.
49. Jiao, J.; Wang, W.; Sulikowski, B.; Weitkamp, J.; Hunger, M., 29Si and 27Al MAS NMR characterization of non-hydrated zeolites Y upon adsorption of ammonia. *Microporous and mesoporous materials* **2006,** *90* (1-3), 246-250.
50. Ravi, M.; Sushkevich, V. L.; van Bokhoven, J. A., On the location of Lewis acidic aluminum in zeolite mordenite and the role of framework-associated aluminum in mediating the switch between Brønsted and Lewis acidity. *Chemical Science* **2021,** *12* (11), 4094-4103.
51. Bregante, D. T.; Potts, D. S.; Kwon, O.; Ayla, E. Z.; Tan, J. Z.; Flaherty, D. W., Effects of Hydrofluoric Acid Concentration on the Density of Silanol Groups and Water Adsorption in Hydrothermally Synthesized Transition-Metal-Substituted Silicalite-1. *Chemistry of Materials* **2020,** *32* (17), 7425-7437.
52. Torres, C.; Potts, D. S.; Flaherty, D. W., Solvent Mediated Interactions on Alkene Epoxidations in Ti-MFI: Effects of Solvent Identity and Silanol Density. *ACS Catalysis* **2023,** *13* (13), 8925-8942.
53. Simon-Masseron, A.; Marques, J.; Lopes, J. M.; Ribeiro, F. R.; Gener, I.; Guisnet, M., Influence of the Si/Al ratio and crystal size on the acidity and activity of HBEA zeolites. *Applied Catalysis A: General* **2007,** *316* (1), 75-82.
54. Louis, B.; Vicente, A.; Fernandez, C.; Valtchev, V., Crystal size–acid sites relationship study of nano-and micrometer-sized zeolite crystals. *The Journal of Physical Chemistry C* **2011,** *115* (38), 18603-18610.
55. Prodinger, S.; Shi, H.; Eckstein, S.; Hu, J. Z.; Olarte, M. V.; Camaioni, D. M.; Derewinski, M. A.; Lercher, J. A., Stability of zeolites in aqueous phase reactions. *Chemistry of Materials* **2017,** *29* (17), 7255-7262.
56. Prodinger, S.; Shi, H.; Wang, H.; Derewinski, M. A.; Lercher, J. A., Impact of structural defects and hydronium ion concentration on the stability of zeolite BEA in aqueous phase. *Applied Catalysis B: Environmental* **2018,** *237*, 996-1002.
57. Bregante, D. T.; Tan, J. Z.; Schultz, R. L.; Ayla, E. Z.; Potts, D. S.; Torres, C.; Flaherty, D. W., Catalytic Consequences of Oxidant, Alkene, and Pore Structures on Alkene Epoxidations within Titanium Silicates. *ACS Catalysis* **2020,** *10* (17), 10169-10184.
58. Tang, B.; Dai, W.; Wu, G.; Guan, N.; Li, L.; Hunger, M., Improved Postsynthesis Strategy to Sn-Beta Zeolites as Lewis Acid Catalysts for the Ring-Opening Hydration of Epoxides. *ACS Catalysis* **2014,** *4* (8), 2801-2810.
59. Ardagh, M. A.; Bregante, D. T.; Flaherty, D. W.; Notestein, J. M., Controlled Deposition of Silica on Titania-Silica to Alter the Active Site Surroundings on Epoxidation Catalysts. *ACS Catalysis* **2020,** *10* (21), 13008-13018.

60. Tan, J. Z.; Bregante, D. T.; Torres, C.; Flaherty, D. W., Transition state stabilization depends on solvent identity, pore size, and hydrophilicity for epoxidations in zeolites. *J Catal* **2022,** *405*, 91-104.
61. Vega-Vila, J. C.; Gounder, R., Quantification of Intraporous Hydrophilic Binding Sites in Lewis Acid Zeolites and Consequences for Sugar Isomerization Catalysis. *ACS Catalysis* **2020,** *10* (20), 12197-12211.
62. Cordon, M. J.; Harris, J. W.; Vega-Vila, J. C.; Bates, J. S.; Kaur, S.; Gupta, M.; Witzke, M. E.; Wegener, E. C.; Miller, J. T.; Flaherty, D. W.; Hibbitts, D. D.; Gounder, R., Dominant Role of Entropy in Stabilizing Sugar Isomerization Transition States within Hydrophobic Zeolite Pores. *Journal of the American Chemical Society* **2018,** *140* (43), 14244-14266.
63. Di Iorio, J. R.; Johnson, B. A.; Román-Leshkov, Y., Ordered Hydrogen-Bonded Alcohol Networks Confined in Lewis Acid Zeolites Accelerate Transfer Hydrogenation Turnover Rates. *Journal of the American Chemical Society* **2020,** *142* (45), 19379-19392.
64. Bregante, D. T.; Thornburg, N. E.; Notestein, J. M.; Flaherty, D. W., Consequences of Confinement for Alkene Epoxidation with Hydrogen Peroxide on Highly Dispersed Group 4 and 5 Metal Oxide Catalysts. *ACS Catalysis* **2018,** *8* (4), 2995-3010.
65. Ayla, E. Z.; Potts, D. S.; Bregante, D. T.; Flaherty, D. W., Alkene Epoxidations with H_2O_2 over Groups 4–6 Metal-Substituted BEA Zeolites: Reactive Intermediates, Reaction Pathways, and Linear Free-Energy Relationships. *ACS Catalysis* **2021,** *11* (1), 139-154.
66. Eaton, T. R.; Boston, A. M.; Thompson, A. B.; Gray, K. A.; Notestein, J. M., Counting Active Sites on Titanium Oxide–Silica Catalysts for Hydrogen Peroxide Activation through In Situ Poisoning with Phenylphosphonic Acid. *ChemCatChem* **2014,** *6* (11), 3215-3222.
67. Eaton, T. R.; Campos, M. P.; Gray, K. A.; Notestein, J. M., Quantifying accessible sites and reactivity on titania–silica (photo) catalysts: Refining TOF calculations. *J Catal* **2014,** *309*, 156-165.
68. Yin, J.; Xu, H.; Wang, B.; Tian, W.; Yin, J.; Jiang, J.; Wu, P., Highly selective 1-pentene epoxidation over Ti-MWW with modified microenvironment of Ti active sites. *Catalysis Science & Technology* **2020,** *10* (17), 6050-6064.
69. Wu, P.; Tatsumi, T., A novel titanosilicate with MWW structure: III. Highly efficient and selective production of glycidol through epoxidation of allyl alcohol with H2O2. *J Catal* **2003,** *214* (2), 317-326.
70. Biaglow, A.; Parrillo, D.; Gorte, R., Characterization of H, Na-Y using amine desorption. *J Catal* **1993,** *144* (1), 193-201.
71. Berdugo-Díaz, C. E.; Manetsch, M. T.; Yun, Y. S.; Lee, J.; Luo, J.; Chen, X.; Flaherty, D., Ester Reduction with H2 on Bifunctional Metal-Acid Catalysts: Implications of Metal Identity on Rates and Selectivities. *Angewandte Chemie International Edition* **2023,** *62* (14), e202216165.
72. Potts, D. S.; Jeyaraj, V. S.; Kwon, O.; Ghosh, R.; Mironenko, A. V.; Flaherty, D. W., Effect of Interactions between Alkyl Chains and Solvent Structures on Lewis Acid Catalyzed Epoxidations. *ACS Catalysis* **2022,** *12* (21), 13372-13393.
73. Goldberg, R. N.; Kishore, N.; Lennen, R. M., Thermodynamic Quantities for the Ionization Reactions of Buffers. *Journal of Physical and Chemical Reference Data* **2002,** *31* (2), 231-370.
74. Berg, R. L.; Vanderzee, C. E., Enthalpies of dilution of sodium carbonate and sodium hydrogen carbonate solutions, and the standard enthalpies of ionization of aqueous carbonic acid, at 298.15 K. *The Journal of Chemical Thermodynamics* **1978,** *10* (11), 1049-1075.
75. Yaws, C. L., *Chemical properties handbook*. McGraw-Hill Education: 1999.
76. Joback, K. G.; Reid, R. C., Estimation of pure-component properties from group-contributions. *Chemical Engineering Communications* **1987,** *57* (1-6), 233-243.
